# Thioacetalation and Multi-Component Thiomethylative Friedel-Crafts Arylation Using BF_3_SMe_2_

**DOI:** 10.1021/acsomega.2c07608

**Published:** 2023-01-19

**Authors:** Marcus Söderström, Christof Matt, Luke R. Odell

**Affiliations:** Department of Medicinal Chemistry, Uppsala University, Uppsala, Biomedical Center, P. O. Box 574, 75123 Uppsala, Sweden

## Abstract

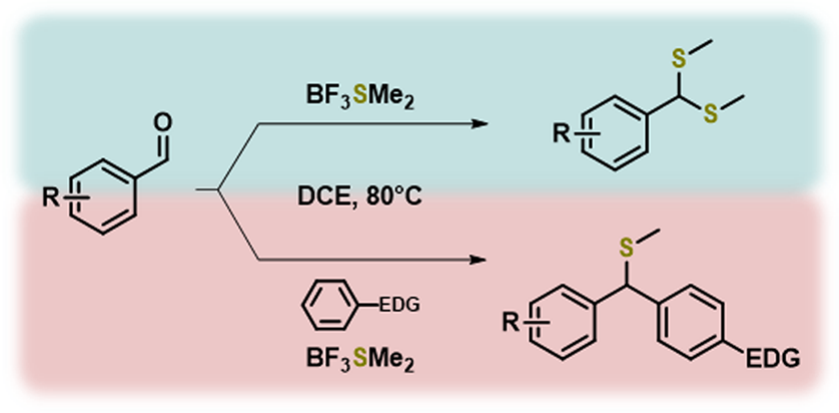

Herein, a method for thioacetalation using BF_3_SMe_2_ is presented. The method allows for convenient and odor-free transformation of aldehydes to methyl-dithioacetals, a simple but sparsely reported structural moiety, in good yields with a diverse set of aromatic aldehydes. In addition, a thiomethylative Friedel-Crafts reaction was discovered, affording thiomethylated diarylmethanes in good to excellent yields. The resulting diarylmethane core is of interest as it is found in many biologically active compounds, and its utility is further demonstrated as a novel precursor to unsymmetrical triarylmethanes. This work also highlights the usefulness and the synthetic capabilities of the readily available reagent BF_3_SMe_2_ beyond its reactivity profile as a dealkylation reagent.

## Introduction

Boron trifluoride etherate is a commonly employed reagent in organic synthesis due to its combination of powerful Lewis acidity and ease of handling.^1−5^ In contrast, the related sulfur analogue boron trifluoride dimethyl sulfide (BF_3_SMe_2_) has received considerably less attention. Perhaps the most well-known and earliest reported use of this system is benzyl group cleavage^6^ using a combination of boron trifluoride etherate and dimethyl sulfide. The combined boron trifluoride complex has since been used almost exclusively for ether dealkylations.^7−18^ Some developments toward uses of the complex have nonetheless been accomplished, such as selective cleavage of di-*tert-*butylsilylene ethers,^19^ selective methoxy cleavage,^20^ and as a borylation reagent.^21^ Recently, we reported BF_3_SMe_2_ as a convenient thiomethylating reagent and also showed that it could reduce nitro groups in certain substrates.^22^ During our investigation, we also found that BF_3_SMe_2_ could produce methyl-dithioacetals from aldehydes albeit in a single example and in low yield.

Thioacetals are the sulfur analogue of the acetal group and are often employed as protecting groups to mask the electrophilic carbon of aldehydes and ketones as they offer increased acid stability.^23^ In addition to carbonyl protection, the thioacetal group can also be employed as a synthetic handle for other transformations and the most well-known example is the Corey–Seebach umpolung reaction.^24,25^ Generally, thioacetals are prepared via the Lewis acid (ZnCl_2_,^26^ BF_3_ etherate,^27^ AlCl_3_,^28^ and TiCl_4_^29^) catalyzed addition of thiols to aldehydes and ketones. Thioacetals can also be accessed from other functional groups such as olefins,^30^ acetals,^31,32^ or propargylic carbons.^33^ Due to the odorous nature of thiols, there have been numerous efforts toward alleviation of this issue. Methods using solid-supported thioacetalation reagents have been reported^34,35^ as well as diacetyl ketene dithioacetals^36−38^ and derivatives thereof^39−41^ for achieving thioacetalation in odorless conditions. However, these methods are limited to cyclic thioacetals and require tedious preparation of the thioacetalating agents.

**Scheme 1:**
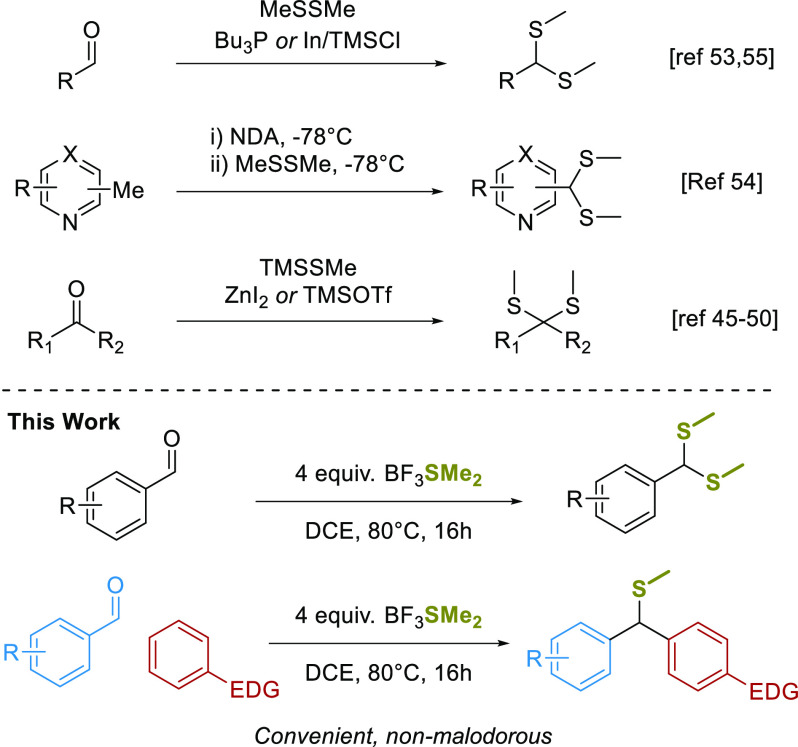
Overview of Synthetic Strategies To Access Methyl-dithioacetals

Advances have also been made in the field of thioacetalation, and there have been mild catalytic methods developed.^31,42−52^ However, despite being the structurally simplest thioacetal derivative, methyl dithioacetals are not found in these examples, most likely due to the highly odorous nature of methanethiol. Besides methanethiol, there are also methods utilizing dimethyldislufide^53−55^ to achieve methyl dithioacetals, although an unpleasant odor is associated with this reagent as well. There are examples that avoid this problem by using methylthiotrimethylsilane,^56−61^ although additional additives are often required and the reagent is rather expensive (Scheme 1).

Due to the usefulness of dithioacetals as a synthetic moiety in organic chemistry and the lack of convenient methods to access methyl dithioacetals, we set out to investigate the use of BF_3_SMe_2_ as a methyl-dithioacetalation reagent. We envisaged a convenient, odor-free method that would further broaden the utility of BF_3_SMe_2_ as a reagent in organic chemistry.

## Results and Discussion

We began our investigation by screening reaction conditions using 2-napthaldehyde (**1a**) as the model substrate (Table 1). Initial attempts found that using 2 equiv of BF_3_SMe_2_ (2 mmol) at 60 °C resulted in 37% of the target dithioacetal **1b**. It was also noted that concentration was important, as increasing the volume of solvent from 1 mL to 5 mL reduced the yield significantly to 10% (entry 2). Next, the effects of temperature were investigated, and we found that 80 °C increased the yield to 44% while increasing it further to 100 °C was detrimental and only traces of the product could be detected (LCMS analysis). With the optimal temperature found, we continued by increasing the amount of BF_3_SMe_2_, and gratifyingly 3 equiv increased the yield to 60%. Increasing the amount further to 4 equiv increased the yield to 68%, while 5 equiv only gave a slight increase of 71%, so 4 equiv was selected for further investigation. Lastly, increasing the reaction time did not give any further improvement, and control experiments removing the Lewis acid (entry 9) or using catalytic amounts of BF_3_OEt_2_ (entry 10) gave no observed reaction.

**Table 1:**
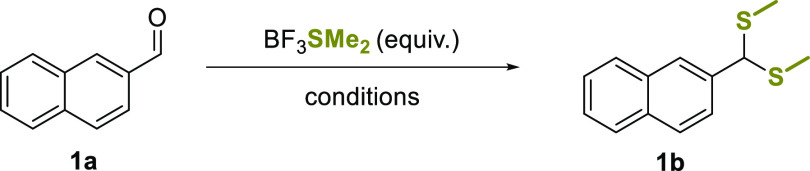
Optimization of the Thioacetalation Reaction^a^

entry	solvent	equiv	time	temp.	yield^b^^a^
1	DCM	2	16 h	60 °C	37%
2	DCM^c^	2	16 h	60 °C	10%
3	DCE	2	16 h	80 °C	44%
4	DCE	2	16 h	100 °C	d
5	DCE	3	16 h	80 °C	60%
**6**	**DCE**	**4**	**16 h**	**80 °C**	**68%**
7	DCE	5	16 h	80 °C	71%
8	DCE	4	24 h	80 °C	68%
9	DCE	4^e^	16 h	80 °C	f
10	DCE	4^g^	16 h	80 °C	f

a Reactions performed at 0.5 mmol scale using 1 mL of solvent.

b Isolated yield.

c 5 mL.

d Not isolated.

e In the absence of BF_3_.

f No reaction.

g Using 4 equiv SMe_2_ with 30 mol % catalytic loading of BF_3_OEt_2_.

With suitable conditions in hand, the scope and limitations of the method were investigated (Table 2). Benzaldehyde and 4-phenylbenzaldehyde gave thioacetals **2b** and **3b** in moderate to good yield as did the halogenated substrates 4-chloro- and 4-fluorobenzaldehyde. Interestingly, 4-bromobenzaldehyde afforded the thiomethyl substituted product **6b**, while 3-bromobenzaldehyde returned the expected thioacetal product **7b** in good yield. This observation might be explained by aldehyde activation by BF_3_, followed by a Br abstraction by DMS (soft nucleophile/soft base),^62^ and attack on the formed DMS-Br complex (Scheme S1, the SI). Electron-poor aromatics quinoline-3-carboxaldehyde and 2-pyridinecarboxaldehyde reacted smoothly to give the desired thioacetals in good yields. More electron-rich aldehydes resulted in low to moderate yields, where 4-(*p*-tolyloxy)benzaldehyde formed a stable dimethylsulfonium derivative as an additional product (**12ba,** SI). We believe that this transformation occurs via a DMS-mediated reduction,^22^ followed by a nucleophilic attack from an additional DMS molecule (Scheme S2, SI). Furthermore, 4-phenoxybenzaldehyde also afforded additional di- and trimeric products presumably resulting from substitution of a thiomethyl group at the 4-position of the electron-rich phenoxyether moiety. Notably, *N*-(4-formylphenyl)acetamide and 4-formylbenzoic acid were compatible with the method, although **14b** resulted in lower yield due to troublesome purification. Finally, 2-phenylbenzaldehyde underwent concomitant thiomethylation and cyclization to afford the thiomethyl fluorene derivative **15b** in 79% yield. Attempts to extend the method to aliphatic aldehydes and ketones were unfortunately unsuccessful.

**Table 2:**
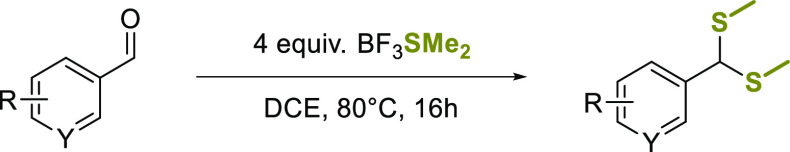

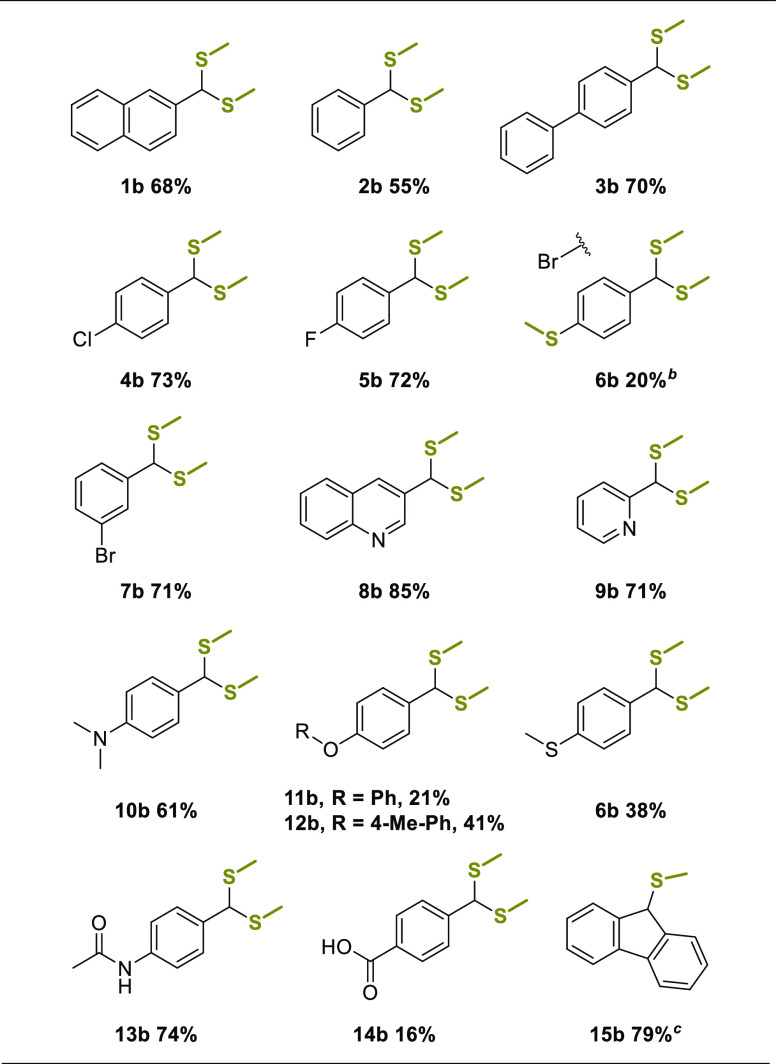
Scope of the Thioacetalation Reaction^a^

a Isolated yield.

b From 4-bromobenzaldehyde.

c From biphenyl-2-carboxaldehyde.

Intrigued by the cyclization of 2-biphenylaldehyde, and the apparent polymerization of 4-phenoxybenzaldehyde, we decided to investigate if the developed conditions could be utilized for cascade thiomethylation/arylation reactions. A literature survey revealed that, indeed, similar reactions have been described before.^63,64^ However, these methods proceed via the use of odorous ethanethiol, and to the best of our knowledge, the use of a thiomethyl source in this context is unprecedented.

**Figure 1:**
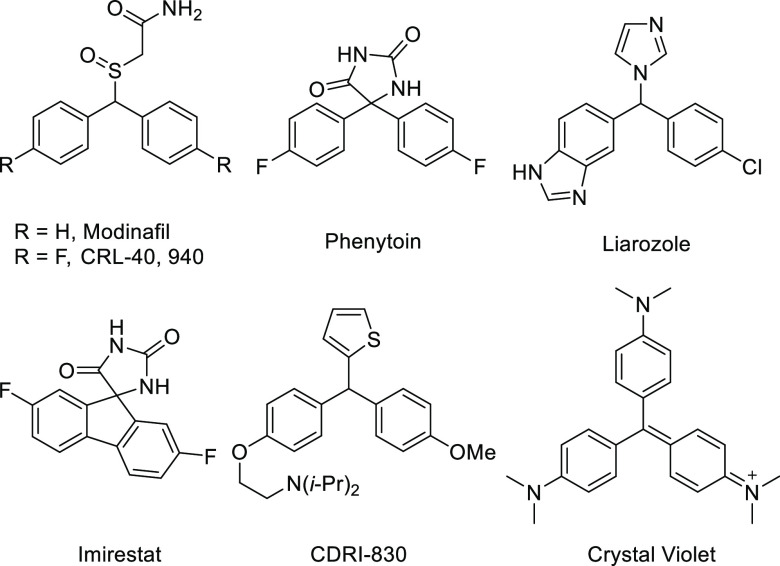
Structures containing the arylmethane motif.

Arylmethanes are of considerable medicinal interest (Figure 1) and can for example be found in central nervous system stimulants modafinil and CRL-40,940,^65,66^ anti-seizure medication phenytoin,^67^ liarozole,^68^ a retinoic acid metabolism-blocking agent, the anti-diabetic imirestat,^69^ and CDRI-830, with anti-tubercular activity.^70^ The structures are also useful for the synthesis of dyes, here exemplified with crystal violet, a compound that is utilized for staining of bacteria,^71^ DNA,^72^ proteins,^73^ and also exhibits antibacterial properties.^74^

With the aim of developing a metal-free cascade thiomethylation/arylation sequence, we set out to explore the possibility of using BF_3_SMe_2_ as a dual-function Lewis acid and non-odorous sulfur source.

**Figure 2:**
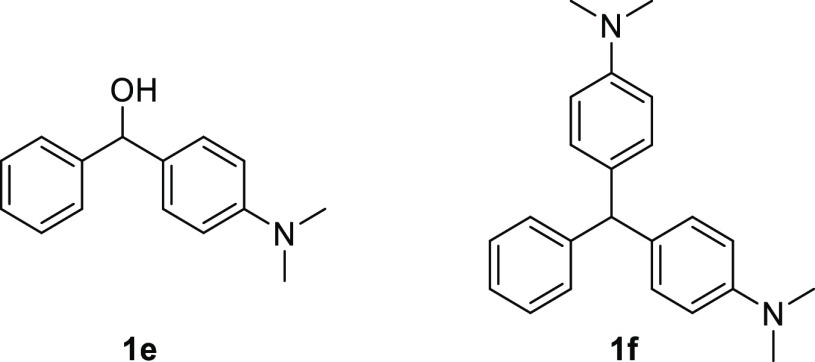
Observed side products during the optimization of the thiomethylative Friedel-Crafts arylation.

As a model substrate, *N,N*-dimethylaniline (**1c**) was chosen as the nucleophile, with benzaldehyde **(2a)** as the electrophile (Table 3). First, we explored the optimal amount of BF_3_SMe_2_ and, to our delight, 2 equivalents at 80 °C gave 79% of the desired product. Increasing the equivalents to 4 resulted in an improved 89% yield, while increasing it further was not beneficial and decreased the yield slightly (entries 1–3). We also found that increasing the amount of aniline or running the reaction in neat conditions gave no increase in yield (entries 4–5). Finally, reducing the reaction temperature to 60 °C resulted in a low yield of **1d** (entry 6) along with alcohol **1e** (27%) and triarylmethane **1f** (22%) side products (Figure 2).

**Table 3:**
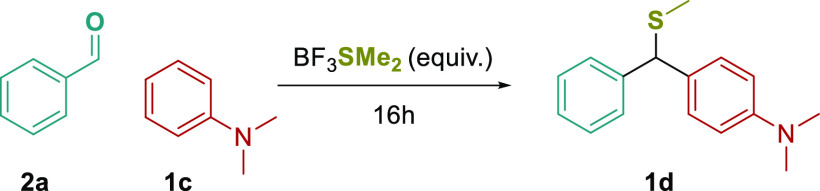
Optimization of the Thiomethylative Friedel-Crafts Arylation Reaction^a^

entry	equiv	**1c** (equiv)	solvent	temp.	yield^b^
1	2	1.1	DCE	80 °C	79%
**2**	**4**	**1.1**	**DCE**	**80 °C**	**89%**
3	6	1.1	DCE	80 °C	86%
4	4	1.3	DCE	80 °C	87%
5	4	1.1		80 °C	86%
6	4	1.1	DCM	60 °C	22%

a Reactions performed at 0.5 mmol scale.

b Isolated yields.

With suitable conditions in hand, we continued exploring the scope of the reaction (Table 4). The substituted anilines *N,N*-diethylaniline and *N*-methyl-4-phenylpiperazine reacted smoothly resulting in products **2d** and **3d** in good yield. Halogenated anilines were also well tolerated, affording products **4-6d** in good to excellent yields. 2-Bromo-*N,N*-dimethylaniline was found to be less reactive, but this could be overcome by switching to solvent-free conditions. Methyl phenyl sulfide also reacted smoothly, although a longer 48 h reaction time was required to reach completion. The conditions were also compatible with diphenyl ether, which gave the *bis*-ether product 8d, in 32% yield. Our attempts to expand the scope to indoles revealed that these nucleophiles were too reactive and, instead of the target thiomethylated product, resulted in the exclusive formation of bis(indolyl)methanes. This was observed despite efforts to reduce reaction temperature and time.

**Table 4:**
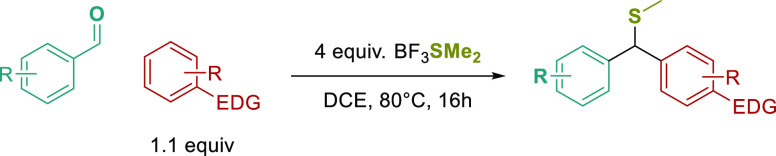

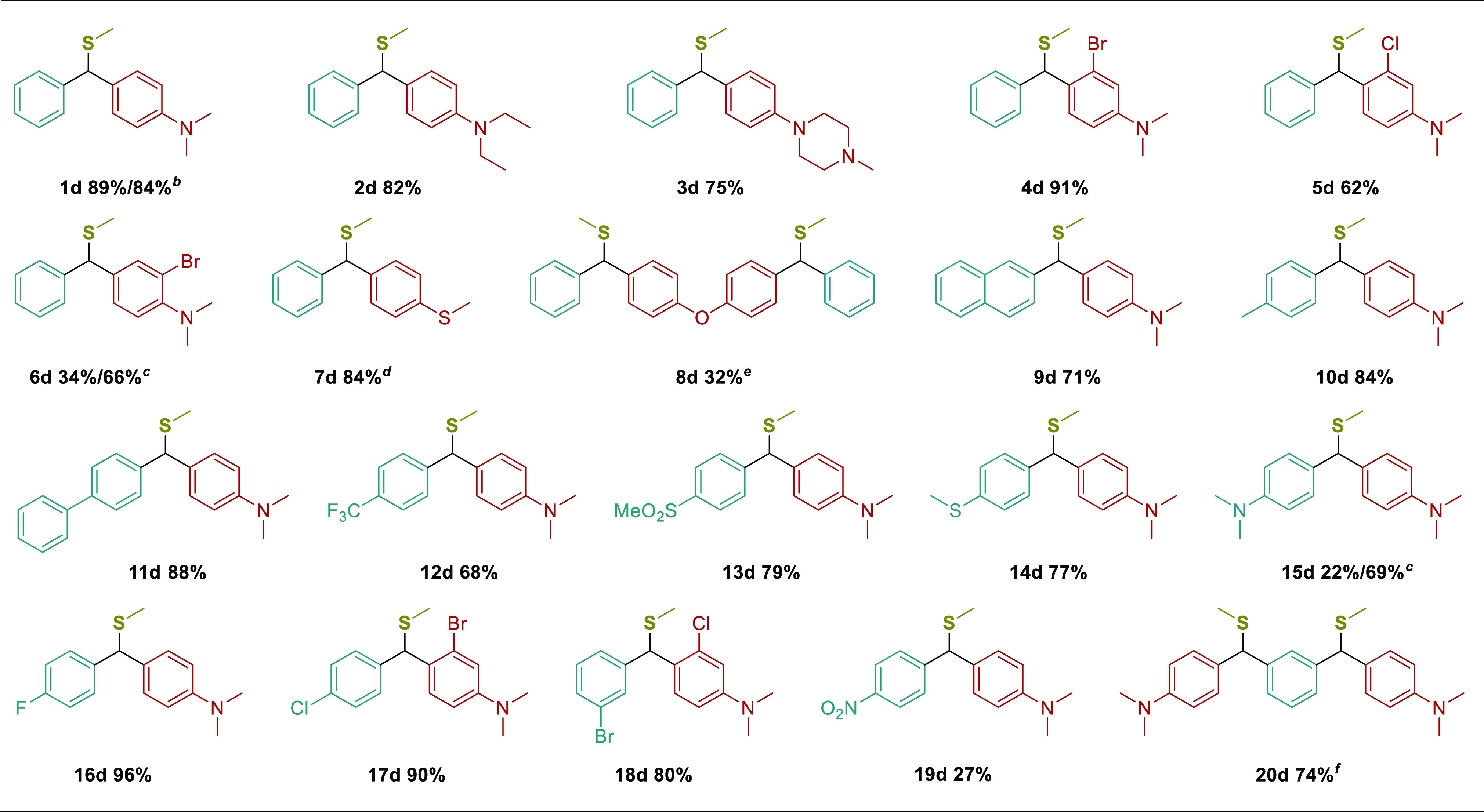
Scope of the Thiomethylative Friedel-Crafts Arylation Reaction^a^

a Isolated yield.

b 5 mmol reaction

c Neat reaction.

d 48 h reaction.

e 0.5 mmol diphenyl ether, 2.2 equiv aldehyde, 8 equiv BF_3_SMe_2_, yield calculated with diphenyl ether as the limiting reagent.

f 2.2 equiv aniline and 8 equiv BF_3_SMe_2_.

Next, we turned our attention to exploring the aldehyde component and 2-napthaldehyde, 4-methylbenzaldehyde, and 4-phenylbenzaldehyde afforded the desired products **9-11d** in good yields. Electron-withdrawing groups such as 4-(trifluoromethyl) and 4-(methylsulfonyl) were well tolerated, as were the electron donating 4-(methylthio) and 4-(dimethylamino) groups. In the case of 4-(dimethylamino)benzaldehyde, lower reactivity was observed, and again, neat conditions could be used to increase the yield. Halogen groups were also well tolerated as can be seen in the formation of compounds **16-18d**. Finally, 1,3-dibenzaldehyde returned the *bis*-diarylmethane product **20d** in good yield. The reaction was also scalable, where performing a 5 mmol reaction of benzaldehyde and *N,N*-dimethylaniline resulted only in a slightly reduced yield of **1d** (84%).

**Scheme 2:**
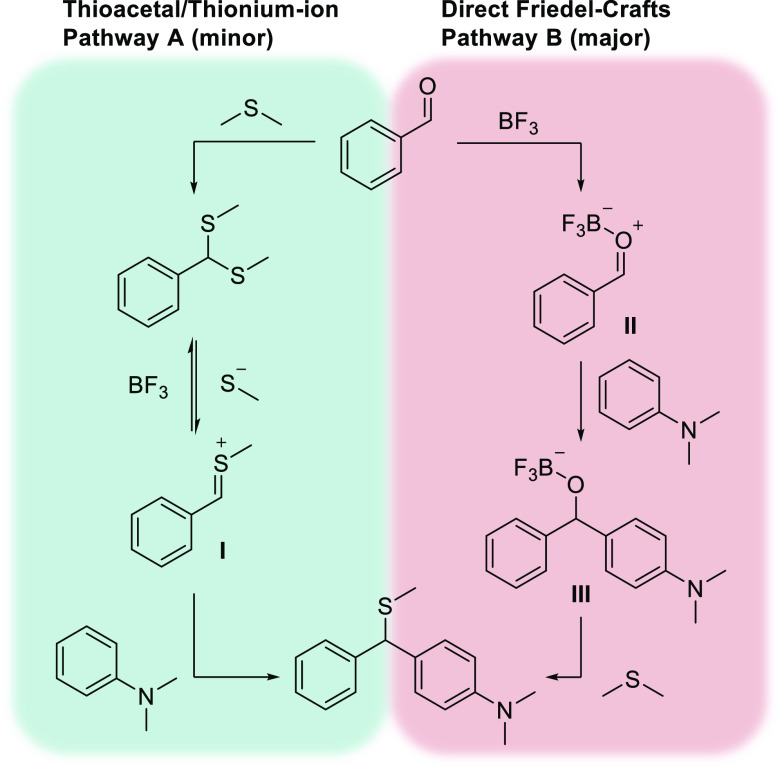
Proposed Pathways for the Thiomethylative Friedel-Crafts Arylation Reaction

We then set out to clarify the reaction mechanism for the transformation, and two suggested pathways are presented in Scheme 2. In path A, the dithioacetal is formed first, followed by the formation of the thionium ion(**I**), and a final Friedel-Crafts type arylation leads to the product. In the second pathway (B), the order of events is reversed with an initial Lewis acid promoted Friedel-Crafts reaction followed by substitution of the activated alcohol(**III**) and a final demethylation, possibly by the eliminated oxygen^75^ or an additional SMe_2_,^6^ to afford the product.

To determine which pathway was more plausible, a set of control reactions were performed (Table 5). First, we reacted dithioacetal **2b** with dimethylaniline in the presence of 4 equiv of BF_3_OEt_2_ at 80 °C. This reaction was rather sluggish, and only 30% of the desired product was isolated (entry 1). We then went on to react benzaldehyde in the absence of a sulfur source, and this resulted in the triarylmethane product (**1f**) in 42% yield. When the amount of aniline was increased to 3 equivalents, the triarylmethane product was isolated in 92% yield (entries 2–3). Next, when using the putative alcohol intermediate **1e** as a substrate, the desired product was isolated in 88% yield (entry 4). Taken together, these findings support the direct Friedel-Crafts pathway (Path B), where attack of aniline is favored over thioacetal formation and is most likely the first step in the reaction. The thioacetal pathway cannot be ruled out as the reaction still proceeds via this intermediate, albeit to a lesser extent.

**Table 5:**
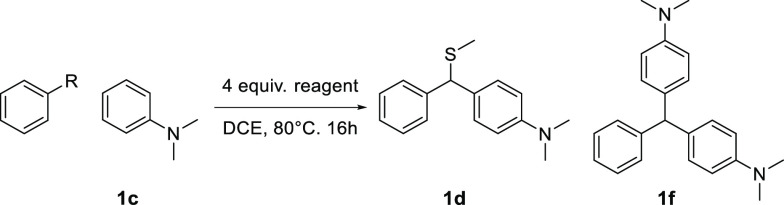

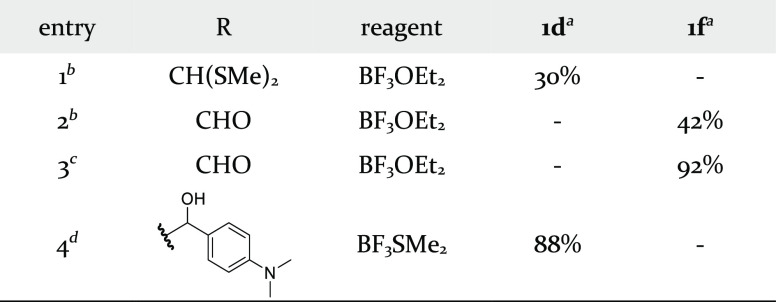
Mechanistic Investigation

a Isolated yield.

b 1.1 equiv **1c**

c 3 equiv **1c**

d In the absence of **1c**.

**Scheme 3:**
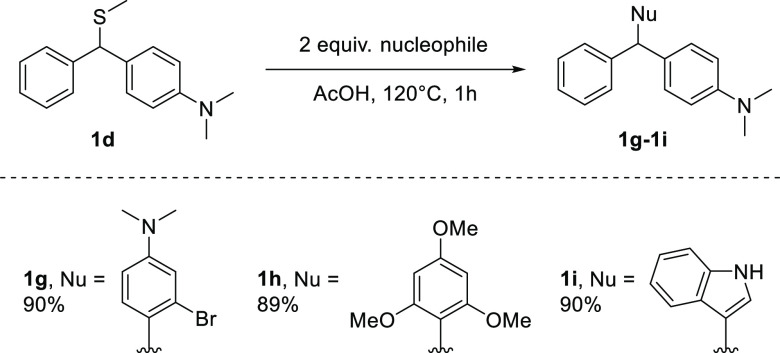
Utility of the Thiomethylated Friedel-Crafts Compounds

During our mechanistic investigations, we noted that displacement of the thiomethyl group occurred upon exposure of **1d** to nucleophiles in certain conditions. We, therefore, reasoned that it could be utilized as a latent handle for further synthesis. After some experimentation, we found that the thiomethyl group was readily substituted under acidic conditions (Scheme 3) in the presence of arene nucleophiles. Thus, treatment of **1d** with 2-bromo-*N,N*-dimethylaniline, 1,3,5-trimethoxybenzene, or 1*H*-indole in AcOH afforded unsymmetrical triarylmethanes **1 g**-**1i** in excellent yields. This represents a valuable new synthetic route to unsymmetrical triarylmethanes that are otherwise difficult to prepare using conventional Friedel-Crafts chemistry^76−80^ due to selectivity issues.

## Conclusions

In summary, our efforts to explore BF_3_SMe_2_ as a versatile and underutilized reagent have resulted in the development of two new thiomethylation reactions. First, the synthesis of methyl dithioacetals from aldehydes has been demonstrated, avoiding odorous methanethiol and expanding the accessibility of this scarcely reported moiety. Furthermore, a three-component cascade thiomethylative Friedel-Crafts reaction was discovered using BF_3_SMe_2_ as a dual Lewis acid and thiomethyl source to afford interesting diarylmethane derivatives. Finally, the synthetic utility of these compounds was demonstrated through the strategic use of the thiomethyl moiety as a latent leaving group in the synthesis of challenging unsymmetrical triarylmethanes. The work described herein serves to further highlight the utility of BF_3_SMe_2_ as a convenient and multifaceted reagent/reactant with applications reaching beyond standard ether deprotections.

## Experimental Section

### General Information

Analytical thin-layer chromatography was performed on silica gel 60 F-254 plates and visualized with UV light. Flash column chromatography was performed on silica gel 60 (40–63 μm). ^1^H and ^13^C spectra were recorded at 400 and 100 MHz, respectively. The chemical shifts for ^1^H NMR and ^13^C NMR are referenced to Tetramethylsilane via residual solvent signals (1H: CDCl_3_ at 7.26 ppm and DMSO-d_6_ at 2.50 ppm; ^13^C: CDCl_3_ at 77.16 ppm and DMSO-d_6_ at 39.52 ppm). Analytical high-performance liquid chromatography/electrospray ionization (ESI)-mass spectrometry was performed using ESI and a C18 column (50 × 3.0 mm^2^, 2.6 μm particle size, 100 Å pore size) with CH3CN/H2O in 0.05% aqueous HCOOH as mobile phase at a flow rate of 1.5 mL/min. LC purity analyses were run using a gradient of 5–100% CH_3_CN/H_2_O in 0.05% aqueous HCOOH as mobile phase at a flow rate of 1.5 mL/min for 5 min unless otherwise stated on a C18 column. High-resolution molecular masses (HRMSs) were determined on a mass spectrometer equipped with an ESI source and time-of-flight unless otherwise stated.

### General Procedure A for Synthesis of Compounds **1b**–**15b** and **12ab**

An oven-dried vial was charged with 0.5 mmol aldehyde. 1,2-Dichloroethane (DCE) (1 mL) was added, followed by 4 equiv (4 mmol, 0.42 mL) BF_3_SMe_2_. The vial was sealed, and the mixture was heated to 80 °C. After 16 h, the mixture was cooled to 0 °C and carefully quenched with 0.5 mL MeOH. The mixture was taken up in 20 mL DCM and poured into a separatory funnel. 10 mL Sat. Na_2_CO_3_ was added and the phases were separated. The aqueous phase was extracted with an additional 2 × 20 mL DCM. The organics were pooled, dried over Na_2_SO_4_, filtered, and concentrated under reduced pressure. The resulting crude material was purified over silica.

### General Procedure B for Synthesis of Compounds **1d**–**20d**

A vial was charged with 0.5 mmol aldehyde and 1.1 equiv (0.55 mmol) nucleophile. DCE (1 mL) was added, followed by 4 equiv (2 mmol, 0.21 mL) BF_3_SMe_2._ The vial was sealed and the mixture was heated to 80 °C. After 16 h, the mixture was cooled to 0 °C and the reaction was quenched with 0.2 mL water. The mixture was poured into 10 mL sat. Na_2_CO_3_ and extracted with 3 × 20 mL DCM. The organics were pooled, dried over Na_2_SO_4_, filtered, and concentrated under reduced pressure. The resulting crude material was purified over silica.

### General Procedure C for Synthesis of Compounds **1g**–**1i**

A vial was charged with 77.2 mg (0.3 mmol) **1d** and 2 equiv (0.6 mmol) nucleophile. AcOH (1 mL) was added, and the mixture was heated to 120 °C for 1 h with the vial open to the atmosphere. The mixture was cooled to ambient temperature and poured into 10 mL sat. Na_2_CO_3_ and extracted with 3 × 20 mL DCM. The organics were pooled, dried over Na_2_SO_4_, filtered, and concentrated under reduced pressure. The resulting crude material was purified over silica.

### (Naphthalen-2-ylmethylene)*bis*(methylsulfane) (**1b**)

Synthesized according to procedure A from 2-napthaldehyde. Purified over silica using 20% DCM in *i*-hexane. Isolated as a white solid (79.9 mg, 68%). ^1^H NMR (400 MHz, CDCl_3_) δ 7.87–7.79 (m, 4H), 7.59 (dd, *J* = 8.5, 1.9 Hz, 1H), 7.55–7.43 (m, 2H), 4.94 (d, *J* = 0.5 Hz, 1H), 2.13 (s, 6H). ^13^C{^1^H} NMR (101 MHz, CDCl_3_) δ 137.0, 133.2, 133.1, 128.7, 128.1, 127.8, 126.5, 126.4, 126.3, 125.9, 56.8, 15.2. HRMS *m/z*: [M – ^–^SMe]^+^ calcd for C_12_H_11_S^+^ 187,0477; found 187.0575.

### (Phenylmethylene)*bis*(methylsulfane) (**2b**)

Synthesized according to procedure A from benzaldehyde. Purified over silica using 2% EtOAc in *i*-hexane. Isolated as a colorless oil (51.0 mg, 55%). ^1^H NMR (400 MHz, CDCl_3_) δ 7.44–7.39 (m, 2H), 7.37–7.31 (m, 2H), 7.30–7.24 (m, 1H), 4.79 (s, 1H), 2.11 (s, 6H). ^13^C{^1^H} NMR (101 MHz, CDCl_3_) δ 139.8, 128.6, 128.0, 127.7, 56.6, 15.1.^57^

### ([1,1′-Biphenyl]-4-ylmethylene)*bis*(methylsulfane) (**3b**)

Synthesized according to procedure A from biphenyl-4-carboxaldehyde. Purified over silica using 20% DCM in *i*-hexane. Isolated as a white solid (90.7 mg, 70%). ^1^H NMR (400 MHz, CDCl_3_) δ 7.62–7.55 (m, 4H), 7.52–7.47 (m, 2H), 7.47–7.41 (m, 2H), 7.38–7.32 (m, 1H), 4.84 (s, 1H), 2.15 (s, 6H). ^13^C{^1^H} NMR (101 MHz, CDCl_3_) δ 140.9, 140.7, 138.8, 128.9, 128.2, 127.5, 127.4, 127.2, 56.4, 15.1. HRMS *m/z*: [M – ^–^SMe]^+^ calcd for C14H13S+ 213.0738; found 213.0732.

### ((4-Chlorophenyl)methylene)*bis*(methylsulfane) (**4b**)

Synthesized according to procedure A from 4-chlorobenzaldehyde. Purified over silica using 10% DCM in *i*-hexane. Isolated as a pale yellow oil (80.6 mg, 73%).^81^^1^H NMR (400 MHz, CDCl_3_) δ 7.39–7.34 (m, 2H), 7.33–7.29 (m, 2H), 4.75 (s, 1H), 2.09 (s, 6H). ^13^C{^1^H} NMR (101 MHz, CDCl_3_) δ 138.4, 133.6, 129.1, 128.8, 55.9, 15.1.

### ((4-Fluorophenyl)methylene)*bis*(methylsulfane) (**5b**)

Synthesized according to procedure A from 4-fluorobenzaldehyde. Purified over silica using 10% DCM in *i*-hexane. Isolated as a clear oil (72.8 mg, 72%). ^1^H NMR (400 MHz, CDCl_3_) δ 7.44–7.36 (m, 2H), 7.07–6.98 (m, 2H), 4.77 (s, 1H), 2.10 (s, 6H). ^13^C{^1^H} NMR (101 MHz, CDCl_3_) δ 162.3 (d, *J* = 246.8 Hz), 135.7 (d, *J* = 3.2 Hz), 129.4 (d, *J* = 8.3 Hz), 115.5 (d, *J* = 21.8 Hz), 55.8, 15.1.^55^

### ((*4*-(Methylthio)phenyl)methylene)*bis*(methylsulfane) (**6b**)

Synthesized according to procedure A from either 4-bromobenzaldehyde or 4-(methylthio)benzaldehyde. From 4-bromobenzaldehyde: Purified over silica using 20–50% DCM in *i*-hexane. Isolated as a pale yellow oil (23.0 mg, 20%). From 4-(methylthio)benzaldehyde: Purified over silica using 20–50% DCM in *i*-hexane. 43.5 mg (38%) isolated as a pale yellow oil. ^1^H NMR (400 MHz, CDCl_3_) δ 7.36–7.31 (m, 2H), 7.24–7.19 (m, 2H), 4.75 (s, 1H), 2.48 (s, 3H), 2.09 (s, 6H). ^13^C{^1^H} NMR (101 MHz, CDCl_3_) δ 138.2, 136.6, 128.2, 126.6, 56.1, 15.8, 15.1. HRMS *m/z*: [M – ^–^SMe]^+^ calcd for C_9_H_11_S_2_^+^ 183.0302; found 183.0297.

### ((3-Bromophenyl)methylene)*bis*(methylsulfane) (**7b**)

Synthesized according to procedure A from 3-bromobenzaldehyde. Purified over silica using 10% DCM in *i*-hexane. Isolated as a white solid (93.0 mg, 71%). ^1^H NMR (400 MHz, CDCl_3_) δ 7.58 (t, *J* = 1.9 Hz, 1H), 7.41 (dt, *J* = 7.9, 1.9, 1.1 Hz, 1H), 7.36 (dt, *J* = 7.9, 1.5 Hz, 1H), 7.21 (t, *J* = 7.9 Hz, 1H), 4.72 (s, 1H), 2.11 (s, 6H). ^13^C{^1^H} NMR (101 MHz, CDCl_3_) δ 142.2, 131.1, 130.8, 130.2, 126.4, 122.7, 55.9, 15.1. HRMS *m/z*: [M – ^–^SMe]^+^ calcd for C_8_H_8_BrS^+^ 214.9525; found 214.9532.

### 3-(*Bis*(Methylthio)methyl)quinolone (**8b**)

Synthesized according to procedure A from 3-quinolinecarboxaldehyde. Purified over silica using 10–20% EtOAc in toluene. Isolated as a clear oil (100.3 mg, 85%). ^1^H NMR (400 MHz, CDCl_3_) δ 8.96 (d, *J* = 2.3 Hz, 1H), 8.16 (d, *J* = 2.3 Hz, 1H), 8.13–8.06 (m, 1H), 7.82 (dd, *J* = 8.2, 1.4 Hz, 1H), 7.71 (ddd, *J* = 8.2, 6.9, 1.4 Hz, 1H), 7.55 (ddd, *J* = 8.2, 6.9, 1.4 Hz, 1H), 4.99 (s, 1H), 2.14 (s, 6H). ^13^C{^1^H} NMR (101 MHz, CDCl_3_) δ 150.8, 147.7, 133.9, 132.6, 129.7, 129.4, 128.0, 127.5, 127.2, 54.1, 14.9.^22^

### 2-(*Bis*(methylthio)methyl)pyridine (**9b**)

Synthesized according to procedure A from 2-pyridinecarboxaldehyde. Purified over silica using 10–25% EtOAc in *i*-hexane. Isolated as a pale yellow oil (65.6 mg, 71%). ^1^H NMR (400 MHz, CDCl_3_) δ 8.53 (dt, *J* = 4.9, 1.8, 1.1 Hz, 1H), 7.68 (td, *J* = 7.7, 1.8 Hz, 1H), 7.47 (dt, *J* = 7.9, 1.1 Hz, 1H), 7.18 (ddd, *J* = 7.5, 4.9, 1.2 Hz, 1H), 4.92 (s, 1H), 2.13 (s, 6H). ^13^C{^1^H} NMR (101 MHz, CDCl_3_) δ 159.4, 149.0, 137.0, 122.6, 122.0, 58.3, 14.5.^54^

### 4-(*Bis*(methylthio)methyl)-*N,N*-dimethylaniline (**10b**)

Synthesized according to procedure A from 4-(dimethylamino)benzaldehyde. Purified over silica using 50% DCM in *i*-hexane. Isolated as a pale yellow oil (69.8 mg, 61%). ^1^H NMR (400 MHz, CDCl_3_) δ 7.33–7.25 (m, 2H), 6.73–6.64 (m, 2H), 4.75 (s, 1H), 2.95 (s, 6H), 2.09 (s, 6H). ^13^C{^1^H} NMR (101 MHz, CDCl_3_) δ 150.3, 128.6, 127.2, 112.3, 56.3, 40.7, 15.2.

HRMS *m/z*: [M + H]^+^ calcd for C_11_H_18_NS_2_ 228.0881; found 228.0880.

### ((4-Phenoxyphenyl)methylene)*bis*(methylsulfane) (**11b**)

Synthesized according to procedure A from 4-phenoxybenzaldehyde. Purified over silica using 20% DCM in *i*-hexane. Isolated as an off-white solid (28.8 mg, 21%). ^1^H NMR (400 MHz, CDCl_3_) δ 7.41–7.31 (m, 4H), 7.15–7.09 (m, 1H), 7.05–7.00 (m, 2H), 6.99–6.94 (m, 2H), 4.78 (s, 1H), 2.12 (s, 6H). ^13^C{^1^H} NMR (101 MHz, CDCl_3_) δ 157.1, 157.0, 134.5, 129.9, 129.1, 123.6, 119.3, 118.7, 56.1, 15.2. HRMS *m/z*: [M – ^–^SMe]^+^ calcd for C_14_H_13_OS^+^ 229.0687; found 229.0681.

### ((4-(*p*-Tolyloxy)phenyl)methylene)*bis*(methylsulfane) (**12b**)

Synthesized according to procedure A from 4-(4-methylphenoxy)benzaldehyde. Purified over silica using 20% DCM in *i*-hexane. Isolated as an off-white solid (60.0 mg, 41%). ^1^H NMR (400 MHz, CDCl_3_) δ 7.40–7.32 (m, 2H), 7.19–7.11 (m, 2H), 6.97–6.89 (m, 4H), 4.77 (s, 1H), 2.34 (s, 3H), 2.11 (s, 6H). ^13^C{^1^H} NMR (101 MHz, CDCl_3_) δ 157.6, 154.5, 134.0, 133.3, 130.4, 129.0, 119.5, 118.1, 56.1, 20.9, 15.2. HRMS *m/z*: [M – ^–^SMe]^+^ calcd for C15H15OS+ 243.0844; found 243.0837.

### *N*-(4-(*Bis*(methylthio)methyl)phenyl)acetamide (**13b**)

Synthesized according to procedure A from 4-acetamidobenzaldehyde. Purified over silica using 25–75% EtOAc in *i*-hexane. Isolated as an off-white solid (89.0 mg, 74%). ^1^H NMR (400 MHz, DMSO-*d*_6_) δ 9.97 (s, 1H), 7.58–7.47 (m, 2H), 7.34–7.26 (m, 2H), 4.99 (s, 1H), 2.04 (s, 6H), 2.03 (s, 2H). ^13^C{^1^H} NMR (101 MHz, DMSO-*d*_6_) δ 168.3, 138.7, 134.4, 127.7, 118.8, 54.8, 24.0, 14.5, 14.3. HRMS *m/z*: [M + H]^+^ calcd for C_11_H_16_NOS_2_ 242.0673; found 242.0684.

### 4-(*Bis*(methylthio)methyl)benzoic Acid (**14b**)

Synthesized according to procedure A from 4-formylbenzoic acid. Purified over silica using 10–50% EtOAc in *i*-hexane. Isolated as a white solid (18.6 mg, 16%). ^1^H NMR (400 MHz, CDCl_3_) δ 8.13–8.06 (m, 2H), 7.57–7.48 (m, 2H), 4.83 (s, 1H), 2.12 (s, 6H). ^13^C{^1^H} NMR (101 MHz, CDCl_3_) δ 171.6, 146.0, 130.7, 128.9, 127.9, 56.2, 15.0. HRMS *m/z*: [M – ^–^SMe]^+^ calcd for C_9_H_9_O_2_S^+^ 181.0323; found 181.0331.

### (9H-Fluoren-9-yl)(methyl)sulfane (**15b**)

Synthesized according to procedure A from biphenyl-2-carboxaldehyde. Purified over silica using 20% DCM in *i*-hexane. Isolated as a white solid (84.0 mg, 79%). ^1^H NMR (400 MHz, CDCl_3_) δ 7.77–7.65 (m, 4H), 7.45–7.32 (m, 4H), 4.88 (s, 1H), 1.41 (s, 3H). ^13^C{^1^H} NMR (101 MHz, CDCl_3_) δ 144.4, 141.0, 128.0, 127.7, 125.4, 119.8, 49.0, 9.7.^82^

### Dimethyl(4-(*p*-tolyloxy)benzyl)sulfonium (**12ba**)

Synthesized according to procedure A from 4-(4-methylphenoxy)benzaldehyde. Purified over silica using 10% MeOH in DCM. Isolated as an off-white solid (42.5 mg, 25%). ^1^H NMR (400 MHz, CDCl_3_) δ 7.40–7.32 (m, 2H), 7.19–7.11 (m, 2H), 6.98–6.86 (m, 4H), 4.58 (s, 2H), 2.82 (s, 6H), 2.34 (s, 3H). ^13^C{^1^H} NMR (101 MHz, CDCl_3_) δ 160.1, 153.3, 134.3, 132.6, 130.7, 120.1, 120.0, 118.6, 46.6, 23.6, 20.9. HRMS *m/z*: [M]^+^ calcd for C_16_H_19_OS+ 259.1157; found 259.1157.

### *N,N*-Dimethyl-4-((methylthio)(phenyl)methyl)aniline (**1d**)

Synthesized according to procedure B from benzaldehyde and *N,N*-dimethylaniline. Purified over silica using 5% EtOAc in *i*-hexane. Isolated as a white solid (114.0 mg, 89%). ^1^H NMR (400 MHz, CDCl_3_) δ 7.47–7.40 (m, 2H), 7.36–7.25 (m, 4H), 7.25–7.18 (m, 1H), 6.73–6.65 (m, 2H), 5.02 (s, 1H), 2.93 (s, 6H), 1.98 (s, 3H). ^13^C{^1^H} NMR (101 MHz, CDCl_3_) δ 149.8, 142.1, 129.1, 129.0, 128.5, 128.4, 127.0, 112.6, 55.7, 40.7, 16.0. HRMS *m/z*: [M + H]^+^ calcd for C_16_H_20_NS 258.1316; found 258.1311.

### *N,N*-Diethyl-4-((methylthio)(phenyl)methyl)aniline (**2d**)

Synthesized according to procedure B from benzaldehyde and *N,N*-diethylaniline. Purified over silica using 5% EtOAc in *i*-hexane. Isolated as a brown liquid (117.3 mg, 82%). ^1^H NMR (400 MHz, CDCl_3_) δ 7.51–7.42 (m, 2H), 7.37–7.29 (m, 2H), 7.29–7.20 (m, 3H), 6.74–6.50 (m, 2H), 5.01 (s, 1H), 3.34 (q, *J* = 7.0 Hz, 4H), 2.00 (s, 3H), 1.16 (t, *J* = 7.0 Hz, 6H). ^13^C{^1^H} NMR (101 MHz, CDCl_3_) δ 147.0, 142.1, 129.3, 128.5, 128.4, 127.7, 126.9, 111.7, 55.7, 44.4, 16.0, 12.7. HRMS *m/z*: [M + H]^+^ calcd for C_18_H_24_NS 286.1629; found 286.1628.

### 1-Methyl-4-(4-((methylthio)(phenyl)methyl)phenyl)piperazine (**3d**)

Synthesized according to procedure B from benzaldehyde and 1-methyl-4-phenylpiperazine. Purified over silica using 2% MeOH and 1% TEA in DCM. Isolated as a brown oil (116.8 mg, 75%). ^1^H NMR (400 MHz, CDCl_3_) δ 7.45–7.38 (m, 2H), 7.34–7.26 (m, 4H), 7.25–7.18 (m, 1H), 6.91–6.83 (m, 2H), 5.01 (s, 1H), 3.25–3.15 (m, 4H), 2.62–2.52 (m, 4H), 2.35 (s, 3H), 1.97 (s, 3H). ^13^C{^1^H} NMR (101 MHz, CDCl_3_) δ 150.2, 141.7, 132.2, 129.1, 128.5, 128.3, 127.0, 115.9, 55.5, 55.1, 48.9, 46.2, 15.9. HRMS *m/z*: [M + H]^+^ calcd for C_19_H_25_N_2_S [M + H]^+^ 313.1738; found 313.1739.

### 3-Bromo-*N,N*-dimethyl-4-((methylthio)(phenyl)methyl)aniline (**4d**)

Synthesized according to procedure B from benzaldehyde and 3-bromo-*N,N*-dimethylaniline. Purified over silica using 5% EtOAc in *i*-hexane. Isolated as a white solid (152.9 mg, 91%). ^1^H NMR (400 MHz, CDCl_3_) δ 7.48 (d, *J* = 8.8 Hz, 1H), 7.46–7.40 (m, 2H), 7.32–7.27 (m, 2H), 7.24–7.17 (m, 1H), 6.85 (d, *J* = 2.7 Hz, 1H), 6.67 (dd, *J* = 8.8, 2.7 Hz, 1H), 5.52 (s, 1H), 2.92 (s, 6H), 2.01 (s, 3H). ^13^C{^1^H} NMR (101 MHz, CDCl_3_) δ 150.4, 141.2, 130.3, 128.5, 128.5, 127.4, 127.1, 125.6, 115.9, 112.1, 53.9, 40.5, 16.1. HRMS *m/z*: [M + H]^+^ calcd for C_16_H_19_BrNS 336.0422; found 336.0413.

### 3-Chloro-*N,N*-dimethyl-4-((methylthio)(phenyl)methyl)aniline (**5d**)

Synthesized according to procedure B from benzaldehyde and 3-chloro-*N,N*-dimethylaniline. Purified over silica using 5% EtOAc in *i*-hexane. Isolated as a yellow oil (90.3 mg, 62%). ^1^H NMR (400 MHz, CDCl_3_) δ 7.52 (d, *J* = 8.7 Hz, 1H), 7.48–7.41 (m, 2H), 7.36–7.28 (m, 2H), 7.25–7.19 (m, 1H), 6.69 (d, *J* = 2.7 Hz, 1H), 6.65 (dd, *J* = 8.7, 2.7 Hz, 1H), 5.55 (s, 1H), 2.93 (s, 6H), 2.03 (s, 3H). ^13^C{^1^H} NMR (101 MHz, CDCl_3_) δ 150.2, 141.2, 134.6, 130.1, 128.5, 128.4, 127.0, 125.8, 112.7, 111.5, 51.3, 40.4, 16.0. HRMS *m/z*: [M + H]^+^ calcd for C_16_H_19_ClNS 292.0927; found 292.0922.

### 2-Bromo-*N,N*-dimethyl-4-((methylthio)(phenyl)methyl)aniline (**6d**)

Synthesized according to modified procedure B from benzaldehyde and 2-bromo-*N,N*-dimethylaniline in neat conditions. Purified over silica using 25–50% DCM in *i*-hexane. Isolated as a yellow oil (110.7 mg, 66%). ^1^H NMR (400 MHz, CDCl_3_) δ 7.61 (dd, *J* = 2.1, 0.5 Hz, 1H), 7.44–7.36 (m, 2H), 7.36–7.27 (m, 3H), 7.28–7.21 (m, 1H), 7.02 (d, *J* = 8.3 Hz, 1H), 4.96 (s, 1H), 2.78 (s, 6H), 1.99 (s, 3H). ^13^C{^1^H} NMR (101 MHz, CDCl_3_) δ 150.9, 141.0, 137.1, 133.7, 128.8, 128.3, 128.0, 127.4, 120.5, 119.2, 55.2, 44.3, 16.1. HRMS *m/z*: [M + H]^+^ calcd for C_16_H_19_BrNS 336.0422; found 336.0427.

### Methyl(4-((methylthio)(phenyl)methyl)phenyl)sulfane (**7d**)

Synthesized according to procedure B from benzaldehyde and thioanisole. 48 h reaction time. Purified over silica using 5% EtOAc in *i*-hexane. Isolated as a yellow oil (108.7 mg, 84%). ^1^H NMR (400 MHz, CDCl_3_) δ 7.48–7.39 (m, 2H), 7.38–7.29 (m, 4H), 7.28–7.19 (m, 3H), 5.03 (s, 1H), 2.47 (s, 3H), 2.00 (s, 3H). ^13^C{^1^H} NMR (101 MHz, CDCl_3_) δ 141.2, 138.3, 137.3, 128.9, 128.7, 128.4, 127.3, 126.8, 55.8, 16.0, 16.0. HRMS (EI^+^) *m/z*: [M]^+^ calcd for C_15_H_16_S_2_ 260.0688; found 260.0693.

### ((Oxybis(4,1-phenylene))*bis*(phenylmethylene))*bis*(methylsulfane) (**8d**)

Synthesized according to a modified procedure B from 0.5 mmol diphenylether and 1.1 mmol (2.2 equiv) benzaldehyde. Purified over silica using 10–20% DCM in *i*-hexane. Isolated as a clear oil (70.5 mg, 32%). ^1^H NMR (400 MHz, CDCl_3_) δ 7.46–7.40 (m, 4H), 7.39–7.30 (m, 8H), 7.28–7.22 (m, 2H), 6.99–6.90 (m, 4H), 5.05 (s, 2H), 2.00 (s, 6H). ^13^C{^1^H} NMR (101 MHz, CDCl_3_) δ 156.3, 141.3, 136.3, 129.7, 128.7, 128.4, 127.3, 118.9, 55.6, 16.0. HRMS *m/z*: [M – ^–^SMe]^+^ calcd for C_27_H_23_OS^+^ 395.1470; found 395.1471.

### *N,N*-Dimethyl-4-((methylthio)(naphthalen-2-yl)methyl)aniline (**9d**)

Synthesized according to procedure B from 2-naphtaldehyde and *N,N*-dimethylaniline. Purified over silica using 5% EtOAc in *i*-hexane. Isolated as a white solid (109.0 mg, 71%). ^1^H NMR (400 MHz, CDCl_3_) δ 7.93–7.88 (m, 1H), 7.88–7.79 (m, 3H), 7.61 (dd, *J* = 8.5, 1.8 Hz, 1H), 7.56–7.40 (m, 2H), 7.39–7.30 (m, 2H), 6.80–6.68 (m, 2H), 5.21 (s, 1H), 2.95 (s, 6H), 2.05 (s, 3H). ^13^C{^1^H} NMR (101 MHz, CDCl_3_) δ 149.8, 139.4, 133.4, 132.7, 129.2, 128.8, 128.3, 128.0, 127.7, 126.8, 126.1, 112.6, 55.8, 40.6, 16.0. HRMS *m/z*: [M + H]^+^ calcd for C_20_H_22_NS 308.1473; found 308.1479.

### *N,N*-Dimethyl-4-((methylthio)(*p*-tolyl)methyl)aniline (**10d**)

Synthesized according to procedure B from *p*-tolualdehyde and *N,N*-dimethylaniline. Purified over silica using 5% EtOAc in *i*-hexane. Isolated as yellow oil (113.7 mg, 84%). ^1^H NMR (400 MHz, CDCl_3_) δ 7.39–7.33 (m, 2H), 7.33–7.26 (m, 2H), 7.19–7.10 (m, 2H), 6.77–6.67 (m, 2H), 5.02 (s, 1H), 2.95 (s, 6H), 2.35 (s, 3H), 2.01 (s, 3H). ^13^C{^1^H} NMR (101 MHz, CDCl_3_) δ 149.7, 139.0, 136.5, 129.3, 129.2, 129.1, 128.2, 112.6, 55.3, 40.7, 21.1, 16.0. HRMS *m/z*: [M + H]^+^ calcd for C_17_H_22_NS 272.1473; found 272.1461.

### 4-([1,1′-Biphenyl]-4-yl(methylthio)methyl)-*N,N*-dimethylaniline (**11d**)

Synthesized according to procedure B from biphenyl-4-caroxaldehyde and *N,N*-dimethylaniline. Purified over silica using 5% EtOAc in *i*-hexane. Isolated as a brown solid (147.2 mg, 88%). ^1^H NMR (400 MHz, CDCl_3_) δ 7.64–7.52 (m, 6H), 7.48–7.42 (m, 2H), 7.39–7.32 (m, 3H), 6.76–6.72 (m, 2H), 5.09 (s, 1H), 2.96 (s, 6H), 2.05 (s, 3H). ^13^C{^1^H} NMR (101 MHz, CDCl_3_) δ 149.8, 141.2, 141.0, 139.8, 129.1, 128.9, 128.8, 128.8, 127.3, 127.1, 112.6, 55.4, 40.7, 16.0. HRMS *m/z*: [M + H]^+^ calcd for C_22_H_24_NS 334.1629; found 334.1627.

### *N,N*-Dimethyl-4-((methylthio)(4-(trifluoromethyl)phenyl)methyl)aniline (**12d**)

Synthesized according to procedure B from 4-(trifluoromethyl)benzaldehyde and *N,N*-dimethylaniline. Purified over silica using 5% EtOAc in *i*-hexane. Isolated as a red oil (111.0 mg, 68%). ^1^H NMR (400 MHz, CDCl_3_) δ 7.62–7.49 (m, 4H), 7.27–7.20 (m, 2H), 6.74–6.62 (m, 2H), 5.04 (s, 1H), 2.93 (s, 6H), 1.99 (s, 3H). ^13^C{^1^H} NMR (101 MHz, CDCl3) δ 150.0, 146.3, 129.9-129.0 (q, *J* = 32.3 Hz), 129.1, 128.7, 127.9, 125.6–125.5 (q, *J* = 3.7 Hz), 123.0, 123.0, 112.6, 55.3, 40.6, 16.0. ^19^F NMR (376 MHz, CDCl_3_) δ −62.4. HRMS *m/z*: [M + H]^+^ calcd for C_17_H_19_F_3_NS 326.1190; found 326.1189.

### *N,N*-Dimethyl-4-((4-(methylsulfonyl)phenyl)(methylthio)methyl)aniline (**13d**)

Synthesized according to procedure B from 4-(methylsulfonyl)benzaldehyde and *N,N*-dimethylaniline. Purified over silica using 20% EtOAc in *i*-hexane. Isolated as a green oil (132.4 mg, 79%). ^1^H NMR (400 MHz, CDCl_3_) δ 7.87 (d, *J* = 8.3 Hz, 2H), 7.63 (d, *J* = 8.3 Hz, 2H), 7.23 (d, *J* = 8.7 Hz, 2H), 6.68 (d, *J* = 8.7 Hz, 2H), 5.05 (s, 1H), 3.02 (s, 3H), 2.93 (s, 6H), 1.98 (s, 3H). ^13^C{^1^H} NMR (101 MHz, CDCl_3_) δ 149.9, 148.7, 138.9, 129.3, 129.0, 127.7, 127.2, 112.5, 55.2, 44.6, 40.5, 15.9. HRMS *m/z*: [M + H]^+^ calcd for C_17_H_22_NO_2_S_2_ 336.1092; found 336.1082.

### *N,N*-Dimethyl-4-((methylthio)(4-(methylthio)phenyl)methyl)aniline (**14d**)

Synthesized according to procedure B from 4-(methylthio)benzaldehyde and *N,N*-dimethylaniline. Purified over silica using 2–5% EtOAc in *i*-hexane. Isolated as a yellow oil (117.0 mg, 77%). ^1^H NMR (400 MHz, CDCl_3_) δ 7.39–7.30 (m, 2H), 7.30–7.21 (m, 2H), 7.25–7.17 (m, 2H), 6.73–6.64 (m, 2H), 4.98 (s, 1H), 2.93 (s, 6H), 2.46 (s, 3H), 1.98 (s, 3H). ^13^C{^1^H} NMR (101 MHz, CDCl_3_) δ 149.8, 139.1, 136.8, 129.1, 128.9, 128.8, 126.9, 112.6, 55.2, 40.7, 16.1, 16.0. HRMS *m/z*: [M + H]^+^ calcd for C_17_H_22_NS_2_ 304.1194; found 304.1201.

### 4,4′-((Methylthio)methylene)*bis*(*N,N*-dimethylaniline) (**15d**)

Synthesized according to modified procedure B from 4-(dimethylamino)benzaldehyde and *N,N*-dimethylaniline in neat conditions. Purified over silica using 5–10% EtOAc in *i*-hexane. Isolated as an off-white solid (103.9 mg, 69%). ^1^H NMR (400 MHz, CDCl_3_) δ 7.32–7.24 (m, 4H), 6.72–6.65 (m, 4H), 4.96 (s, 1H), 2.92 (s, 12H), 1.97 (s, 3H). ^13^C{^1^H} NMR (101 MHz, CDCl_3_) δ 149.7, 129.9, 129.1, 112.7, 55.1, 40.8, 16.0. HRMS *m/z*: [M + H]^+^ calcd for C_18_H_25_N_2_S 301.1733; found 301.1734.

### 4-((4-Fluorophenyl)(methylthio)methyl)-*N,N*-dimethylaniline (**16d**)

Synthesized according to procedure B from 4-fluorobenzaldehyde and *N,N*-dimethylaniline. Purified over silica using 5% EtOAc in *i*-Hexane. Isolated as a white solid (132.4 mg, 96%). ^1^H NMR (400 MHz, CDCl_3_) δ 7.45–7.38 (m, 2H), 7.30–7.24 (m, 2H), 7.05–6.98 (m, 2H), 6.74–6.68 (m, 2H), 5.01 (s, 1H), 2.95 (s, 6H), 1.99 (s, 3H). ^13^C{^1^H} NMR (101 MHz, CDCl_3_) δ 161.8 (d, *J* = 245.3 Hz), 149.8, 137.9 (d, *J* = 3.1 Hz), 129.9 (d, *J* = 8.0 Hz), 129.0, 128.7, 115.3 (d, *J* = 21.4 Hz), 112.6, 54.9, 40.6, 16.0. ^19^F NMR (376 MHz, CDCl_3_) δ −115.9, −116.0 (m). HRMS *m/z*: [M + H]^+^ calcd for C_16_H_19_FNS 276.1222; found 276.1212.

### 3-Bromo-4-((4-chlorophenyl)(methylthio)methyl)-*N,N*-dimethylaniline (**17d**)

Synthesized according to procedure B from 4-chlorobenzaldehyde and 3-bromo-*N,N*-dimethylaniline. Purified over silica using 10–20% DCM in *i*-hexane. Isolated as a clear oil (167.3 mg, 90%). ^1^H NMR (400 MHz, CDCl_3_) δ 7.45 (d, *J* = 8.8 Hz, 1H), 7.40–7.32 (m, 2H), 7.30–7.22 (m, 2H), 6.86 (d, *J* = 2.7 Hz, 1H), 6.68 (dd, *J* = 8.8, 2.7 Hz, 1H), 5.48 (s, 1H), 2.93 (s, 6H), 2.01 (s, 3H). ^13^C{^1^H} NMR (101 MHz, CDCl_3_) δ 150.5, 139.8, 132.8, 130.1, 129.8, 128.7, 126.7, 125.6, 115.9, 112.1, 53.3, 40.4, 16.1. HRMS *m/z*: [M + H]^+^ calcd for C_16_H_18_BrClNS [M + H]^+^ 370.0032; found 370.0038.

### 4-((3-Bromophenyl)(methylthio)methyl)-3-chloro-*N,N*-dimethylaniline (**18d**)

Synthesized according to procedure B from 3-bromobenzaldehyde and 3-chloro-*N,N*-dimethylaniline. Purified over silica using 10–20% DCM in *i*-hexane. Isolated as a clear oil (148.7 mg, 80%). ^1^H NMR (400 MHz, CDCl_3_) δ 7.60–7.54 (m, 1H), 7.45 (d, *J* = 8.7 Hz, 1H), 7.39–7.30 (m, 2H), 7.16 (t, *J* = 7.8 Hz, 1H), 6.67 (d, *J* = 2.7 Hz, 1H), 6.63 (dd, *J* = 8.7, 2.7 Hz, 1H), 5.47 (s, 1H), 2.94 (s, 6H), 2.02 (s, 3H). ^13^C{^1^H} NMR (101 MHz, CDCl_3_) δ 150.4, 143.6, 134.6, 131.4, 130.2, 130.1, 130.0, 127.2, 124.9, 122.6, 112.7, 111.5, 50.9, 40.4, 16.1. HRMS *m/z*: [M + H]^+^ calcd for C_16_H_18_BrClNS 370.0032; found 370.0026.

### *N,N*-Dimethyl-4-((methylthio)(4-nitrophenyl)methyl)aniline (**19d**)

Synthesized according to procedure B from 4-nitrobenzaldehyde and *N,N*-dimethylaniline. Purified over silica using 20% EtOAc in *i*-hexane. Isolated as an orange solid (40.9 mg, 27%). ^1^H NMR (400 MHz, CDCl_3_) δ 8.16 (d, *J* = 8.8 Hz, 2H), 7.59 (d, *J* = 8.8 Hz, 2H), 7.22 (d, *J* = 8.8 Hz, 2H), 6.68 (d, *J* = 8.8 Hz, 2H), 5.06 (s, 1H), 2.94 (s, 6H), 1.99 (s, 3H). ^13^C{^1^H} NMR (101 MHz, CDCl_3_) δ 150.1, 149.8, 146.9, 129.2, 129.1, 127.1, 123.9, 112.6, 55.2, 40.6, 16.0. HRMS *m/z*: [M + H]^+^ calcd for C_16_H_19_N_2_O_2_S 303.1167; found 303.1162.

### 4,4′-(1,3-Phenylenebis((methylthio)methylene))*bis*(*N,N*-dimethylaniline) (**20d**)

Synthesized according to modified procedure B from 0.5 mmol isophtalaldehyde and 1.1 mmol (2.2 equiv) *N,N*-dimethylaniline. Purified over silica using 5–20% DCM in *i*-hexane. Isolated as an off-white solid (160.5 mg, 74%). ^1^H NMR (400 MHz, CDCl_3_) δ 7.56 (td, *J* = 1.8, 0.7 Hz, 1H), 7.31–7.22 (m, 7H), 6.71–6.65 (m, 4H), 5.00 (s, 2H), 2.93 (s, 12H), 1.96 (s, 6H). ^13^C{^1^H} NMR (101 MHz, CDCl_3_) δ 149.7, 142.0, 142.0, 129.2, 129.1, 129.0, 128.7, 128.6, 128.5, 127.0, 112.6, 55.6, 40.7, 16.0. HRMS *m/z*: [M + H]^+^ calcd for C_26_H_33_N_2_S_2_ [M + H]^+^ 437.2085; found 437.2071.

### 4,4′-(Phenylmethylene)*bis*(*N,N*-dimethylaniline) (**1f**)

A vial was charged with 51.3 mg (0.5 mmol) benzaldehyde and 3 equiv (1.5 mmol, 185 mg) *N,N*-dimethylaniline. DCE (1 mL) was added, followed by 4 equiv (2 mmol, 0.21 mL) BF_3_OEt_2._ The vial was sealed, and the mixture was heated to 80 °C. After 16 h, the mixture was cooled to 0 °C, and 0.2 mL water was added. The mixture was poured into 10 mL Sat. Na_2_CO_3_ and extracted with 3 × 20 mL DCM. The organics were pooled, dried over Na_2_SO_4_, filtered, and concentrated under reduced pressure. The resulting crude material was purified over silica using 5–10% EtOAc in *i*-hexane. Isolated as a brown oil (165.0 mg, 92%). ^1^H NMR (400 MHz, CDCl_3_) δ 7.32–7.21 (m, 2H), 7.21–7.16 (m, 1H), 7.16–7.10 (m, 2H), 7.04–6.95 (m, 4H), 6.73–6.63 (m, 4H), 5.39 (s, 1H), 2.92 (s, 12H). ^13^C{^1^H} NMR (101 MHz, CDCl_3_) δ 149.1, 145.6, 133.0, 130.1, 129.5, 128.2, 125.9, 112.7, 55.1, 40.9.^83^

### 3-Bromo-4-((4-(dimethylamino)phenyl)(phenyl)methyl)-*N,N*-dimethylaniline (**1g**)

Synthesized according to procedure C with 3-bromo-*N,N*-dimethylaniline as a nucleophile. Purified over silica using 5–10% EtOAc in *i*-hexane. Isolated as a white solid (110.1 mg, 90%). ^1^H NMR (400 MHz, CDCl_3_) δ 7.31–7.23 (m, 2H), 7.23–7.15 (m, 1H), 7.13–7.06 (m, 2H), 6.99–6.90 (m, 3H), 6.80 (d, *J* = 8.7 Hz, 1H), 6.71–6.63 (m, 2H), 6.57 (dd, *J* = 8.7, 2.7 Hz, 1H), 5.76 (s, 1H), 2.92 (s, 6H), 2.91 (s, 6H). ^13^C{^1^H} NMR (101 MHz, CDCl_3_) δ 150.0, 149.1, 144.4, 131.7, 131.5, 131.4, 130.2, 129.6, 128.2, 126.3, 126.0, 116.5, 112.6, 111.6, 54.2, 40.8, 40.6. HRMS *m/z*: [M + H]^+^ calcd for C_23_H_26_BrN_2_ 409.1279; found 409.1284.

### *N,N*-Dimethyl-4-(phenyl(2,4,6-trimethoxyphenyl)methyl)aniline (**1h**)

Synthesized according to procedure C with 1,3,5-trimethoxybenzene as nucleophile. Purified over silica using 5–10% EtOAc in *i*-hexane. Isolated as a pink oil (101.0 mg, 89%). ^1^H NMR (400 MHz, CDCl_3_) δ 7.34–7.15 (m, 7H), 6.85–6.78 (m, 2H), 6.23 (s, 2H), 6.07 (s, 1H), 3.88 (s, 3H), 3.67 (s, 6H), 3.00 (s, 6H). ^13^C{^1^H} NMR (101 MHz, CDCl_3_) δ 159.9, 159.2, 145.2, 130.1, 129.0, 127.5, 125.1, 114.2, 113.0, 91.8, 55.8, 55.3, 44.3, 41.4. HRMS *m/z*: [M + H]^+^ calcd for C_24_H_28_NO_3_ 378.2069; found 378.2063.

### 4-((1*H*-Indol-3-yl)(phenyl)methyl)-*N,N*-dimethylaniline (**1i**)

Synthesized according to procedure C with 1*H*-indole as nucleophile. Purified over silica using 2% EtOAc in toluene. Isolated as a white solid (88.0 mg, 90%). ^1^H NMR (400 MHz, CDCl_3_) δ 7.91 (s, 1H), 7.34 (dt, *J* = 8.2, 0.9 Hz, 1H), 7.32–7.17 (m, 6H), 7.19–7.13 (m, 1H), 7.13–7.08 (m, 2H), 6.99 (ddd, *J* = 8.0, 7.1, 1.0 Hz, 1H), 6.73–6.64 (m, 2H), 6.59 (dd, *J* = 2.4, 1.1 Hz, 1H), 5.59 (s, 1H), 2.92 (s, 6H). ^13^C{^1^H} NMR (101 MHz, CDCl_3_) δ 149.2, 144.9, 136.9, 132.3, 129.7, 129.1, 128.3, 127.3, 126.1, 124.1, 122.1, 120.8, 120.2, 119.4, 112.7, 111.1, 48.0, 40.9.^77^

## Supporting Information Available

NMR spectra (^1^H NMR and ^13^C NMR) of all compounds (PDF)
