# Supplementary material for: Thioacetalation and Multi-Component Thiomethylative Friedel-Crafts Arylation Using BF3SMe2
Source: ACS Omega. 2023 Jan 19;8(4):4320–30. doi: 10.1021/acsomega.2c07608 (PMC9893757; doi:10.1021/acsomega.2c07608)
Supplement: Supplementary file 1 — ao2c07608_si_001.pdf [file ao2c07608_si_001.pdf]

Thioacetalation and Multi-Component Thiomethylative Friedel-Crafts Arylation Using BF_3SMe_2

Marcus Söderström, Christof Matt, Luke R. Odell*

Department of Medicinal Chemistry, Uppsala University, Uppsala, Biomedical Center, P. O. Box 574, 75123 Uppsala, Sweden

Table of Contents

Proposed Mechanisms 5

Scheme S1. Proposed reaction mechanism for dehalogenation and substitution of 4-bromobenzaldehyde 5

Scheme S2. Proposed reaction mechanism for formation of dimethyl(4-(p-tolyloxy)benzyl)sulfonium (**12ba**) from 4-(4-methylphenoxy)benzaldehyde. 5

Additional experimental data 6

Experimental data and compound characterization of compounds **1e** and **1f**: Thiomethylatie Fridel-Crafts optimization reaction at 60°C. 6

Experimental Data for mechanistic investigation. 6

Synthesis of 1-methyl-4-phenylpiperazine (**3c**). 7

NMR Spectra 8

Figure S1. ^{1}H-NMR (400 MHz $CDCl_3$) of compound **1b.** 8

Figure S2. ^{13}C{^{1}H}-NMR (101 MHz, $CDCl_3$) of compound **1b**. 9

Figure S3. ^{1}H-NMR (400 MHz $CDCl_3$) of compound **2b.** 10

Figure S4. ^{13}C{^{1}H}-NMR (101 MHz, $CDCl_3$) of compound **2b**. 11

Figure S5. ^{1}H-NMR (400 MHz $CDCl_3$) of compound **3b.** 12

Figure S6. ^{13}C{^{1}H}-NMR (101 MHz, $CDCl_3$) of compound **3b**. 13

Figure S7. 1H-NMR (400 MHz $CDCl_3$) of compound **4b**. 14

Figure S8. ^{13}C{^{1}H}-NMR (101 MHz, $CDCl_3$) of compound **4b.** 15

Figure S9. ^{1}H-NMR (400 MHz $CDCl_3$) of compound **5b.** 16

Figure S10. ^{13}C{^{1}H}-NMR (101 MHz, $CDCl_3$) of compound **5b.** 17

Figure S11. ^{1}H-NMR (400 MHz $CDCl_3$) of compound **6b.** 18

Figure S12. ^{13}C{^{1}H}-NMR (101 MHz, $CDCl_3$) of compound **6b.** 19

Figure S13. ^{1}H-NMR (400 MHz $CDCl_3$) of compound **7b.** 20

Figure S14. ^{13}C{^{1}H}-NMR (101 MHz, $CDCl_3$) of compound **7b.** 21

Figure S15. ^{1}H-NMR (400 MHz $CDCl_3$) of compound **8b.** 22

Figure S16. ^{13}C{^{1}H}-NMR (101 MHz, $CDCl_3$) of compound **8b.** 23

Figure S17. ^{1}H-NMR (400 MHz $CDCl_3$) of compound **9b.** 24

Figure S18. ^{13}C{^{1}H}-NMR (101 MHz, $CDCl_3$) of compound **9b.** 25

Figure S19. ^{1}H-NMR (400 MHz $CDCl_3$) of compound **10b.** 26

Figure S20. ^{13}C{^{1}H}-NMR (101 MHz, $CDCl_3$) of compound **10b.** 27

Figure S21. ^{1}H-NMR (400 MHz $CDCl_3$) of compound **11b.** 28

Figure S22. ^{13}C{^{1}H}-NMR (101 MHz, $CDCl_3$) of compound **11b.** 29

Figure S23. ^{1}H-NMR (400 MHz $CDCl_3$) of compound **12b.** 30
Figure S24. ^{13}C{^{1}H}-NMR (101 MHz, $CDCl_3$) of compound **12b.** 31
Figure S25. ^{1}H-NMR (400 MHz DMSO-d_6) of compound **13b.** 32
Figure S26. ^{13}C{^{1}H}-NMR (101 MHz, DMSO-d_6) of compound **13b.** 33
Figure S27. ^{1}H-NMR (400 MHz $CDCl_3$) of compound **14b.** 34
Figure S28. ^{13}C{^{1}H}-NMR (101 MHz, $CDCl_3$) of compound **14b.** 35
Figure S29. ^{1}H-NMR (400 MHz $CDCl_3$) of compound **15b.** 36
Figure S30. ^{13}C{^{1}H}-NMR (101 MHz, $CDCl_3$) of compound 1**5b.** 37
Figure S31. ^{1}H-NMR (400 MHz $CDCl_3$) of compound **12ba.** 38
Figure S32. ^{13}C{^{1}H}-NMR (101 MHz, $CDCl_3$) of compound **12ba.** 39
Figure S33. ^{1}H-NMR (400 MHz $CDCl_3$) of compound **1d.** 40
Figure S34. ^{13}C{^{1}H}-NMR (101 MHz, $CDCl_3$) of compound **1d.** 41
Figure S35. ^{1}H-NMR (400 MHz CDCl3) of compound **2d**........ 42
Figure S36. ^{13}C{^{1}H}-NMR (101 MHz, $CDCl_3$) of compound **2d.** 43
Figure S37. ^{1}H-NMR (400 MHz $CDCl_3$) of compound **3d.** 44
Figure S38. ^{13}C{^{1}H}-NMR (101 MHz, $CDCl_3$) of compound **3d.** 45
Figure S39. ^{1}H-NMR (400 MHz $CDCl_3$) of compound **4d.** 46
Figure S40. ^{13}C{^{1}H}-NMR (101 MHz, $CDCl_3$) of compound **4d.** 47
Figure S41. ^{1}H-NMR (400 MHz $CDCl_3$) of compound **5d.**........ 48
Figure S42. ^{13}C{^{1}H}-NMR (101 MHz, $CDCl_3$) of compound **5d.**........ 49
Figure S43. ^{1}H-NMR (400 MHz $CDCl_3$) of compound **6d.** 50
Figure S44. ^{13}C{^{1}H}-NMR (101 MHz, $CDCl_3$) of compound **6d.** 51
Figure S45. ^{1}H-NMR (400 MHz $CDCl_3$) of compound **7d.** 52
Figure S46. ^{13}C{^{1}H}-NMR (101 MHz, $CDCl_3$) of compound **7d.** 53
Figure S47. ^{1}H-NMR (400 MHz $CDCl_3$) of compound **8d.** 54
Figure S48. ^{13}C{^{1}H}-NMR (101 MHz, $CDCl_3$) of compound **8d.** 55
Figure S49. ^{1}H-NMR (400 MHz $CDCl_3$) of compound **9d.**........ 56
Figure S50. ^{13}C{^{1}H}-NMR (101 MHz, $CDCl_3$) of compound **9d**. 57
Figure S51. ^{1}H-NMR (400 MHz $CDCl_3$) of compound **10d.** 58
Figure S52. ^{13}C{^{1}H}-NMR (101 MHz, $CDCl_3$) of compound **10d.** 59
Figure S53. ^{1}H-NMR (400 MHz $CDCl_3$) of compound **11d.**........ 60
Figure S54. ^{13}C{^{1}H}-NMR (101 MHz, $CDCl_3$) of compound **11d.** 61
Figure S55. ^{1}H-NMR (400 MHz $CDCl_3$) of compound **12d.**........ 62

Figure S56. $^{13}C\{^1H\}$-NMR (101 MHz, $CDCl_3$) of compound **12d.** 63
Figure S57. ^{19}F-NMR (376 MHz, $CDCl_3$) of compound **12d**........ 64
Figure S58. ^1H-NMR (400 MHz $CDCl_3$) of compound 1**3d.**........ 65
Figure S59. $^{13}C\{^1H\}$-NMR (101 MHz, $CDCl_3$) of compound **13d.** 66
Figure S60. ^1H-NMR (400 MHz $CDCl_3$) of compound **14d**. 67
Figure S61. $^{13}C\{^1H\}$-NMR (101 MHz, $CDCl_3$) of compound **14d.** 68
Figure S62. ^1H-NMR (400 MHz $CDCl_3$) of compound **15d.**........ 69
Figure S63. $^{13}C\{^1H\}$-NMR (101 MHz, $CDCl_3$) of compound **15d.** 70
Figure S64. ^1H-NMR (400 MHz $CDCl_3$) of compound **16d.** 71
Figure S65. $^{13}C\{^1H\}$-NMR (101 MHz, $CDCl_3$) of compound **16d.** 72
Figure S66. ^{19}F-NMR (376 MHz, $CDCl_3$) of compound **16d**........ 73
Figure S67. ^1H-NMR (400 MHz $CDCl_3$) of compound **17d.**........ 74
Figure S68. $^{13}C\{^1H\}$-NMR (101 MHz, $CDCl_3$) of compound **17d.** 75
Figure S69. ^1H-NMR (400 MHz $CDCl_3$) of compound **18d.**........ 76
Figure S70. $^{13}C\{^1H\}$-NMR (101 MHz, $CDCl_3$) of compound **18d.** 77
Figure S71. ^1H-NMR (400 MHz $CDCl_3$) of compound **19d.**........ 78
Figure S72. $^{13}C\{^1H\}$-NMR (101 MHz, $CDCl_3$) of compound **19d.** 79
Figure S73. ^1H-NMR (400 MHz $CDCl_3$) of compound **20d.**........ 80
Figure S74. $^{13}C\{^1H\}$-NMR (101 MHz, $CDCl_3$) of compound **20d.** 81
Figure S75. Effect of heating the $^{13}C\{^1H\}$-NMR (101 MHz, $CDCl_3$) sample of **20d**. 82
Figure S76. ^1H-NMR (400 MHz $CDCl_3$) of compound **1f.**........ 83
Figure S77. $^{13}C\{^1H\}$-NMR (101 MHz, $CDCl_3$) of compound **1f.** 84
Figure S78. ^1H-NMR (400 MHz $CDCl_3$) of compound **1g.**........ 85
Figure S79. $^{13}C\{^1H\}$-NMR (101 MHz, $CDCl_3$) of compound **1g.**........ 86
Figure S80. ^1H-NMR (400 MHz $CDCl_3$) of compound **1h.**........ 87
Figure S81. $^{13}C\{^1H\}$-NMR (101 MHz, $CDCl_3$) of compound **1h.** 88
Figure S82. ^1H-NMR (400 MHz $CDCl_3$) of compound **1i.**........ 89
Figure S83. $^{13}C\{^1H\}$-NMR (101 MHz, $CDCl_3$) of compound **1i.** 90
Figure S84. 1H-NMR (400 MHz CDCl3) of compound **1e**. 91
Figure S85. 13C{1H}-NMR (101 MHz, CDCl3) of compound **1e**. 92
Figure S86. 1H-NMR (400 MHz CDCl3) of compound **3c**. 93
Figure S87. 13C{1H}-NMR (101 MHz, CDCl3) of compound **3c**........ 94

Proposed Mechanisms

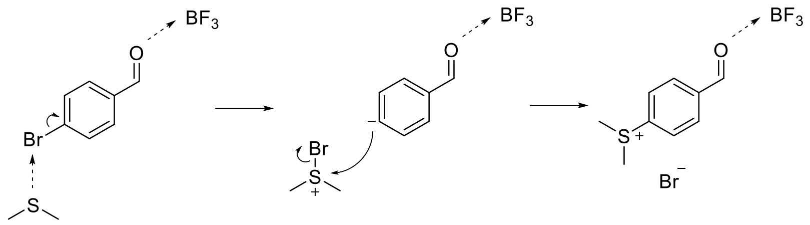

Scheme S1. Proposed reaction mechanism for dehalogenation and substitution of 4-bromobenzaldehyde

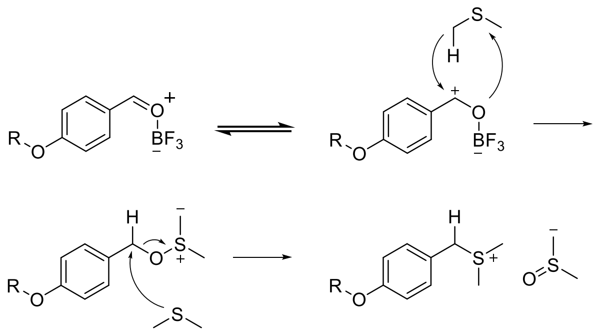

Scheme S2. Proposed reaction mechanism for formation of dimethyl(4-(p-tolyloxy)benzyl)sulfonium (**12ba)** from 4-(4-methylphenoxy)benzaldehyde.

Additional experimental data

Experimental data and compound characterization of compounds 1e and 1f: Thiomethylative Fridel-Crafts optimization reaction at 60ºC.

Table 3, Entry 6. Synthesis of (4-(dimethylamino)phenyl)(phenyl)methanol (**1e**) and 4,4'-(phenylmethylene)*bis*(*N,N*-dimethylaniline) (**1f**). Synthesized according to procedure B from benzaldehyde and *N,N*-dimethylaniline, at 60ºC in DCM. Purified over silica using 2-25% EtOAc in *i*-Hexane.

In order of elution on silica column:

1d isolated as a white solid (28.3 mg, 22%) ^{1}H NMR (400 MHz, $CDCl_3$) *δ* 7.47 – 7.40 (m, 2H), 7.36 – 7.25 (m, 4H), 7.25 – 7.18 (m, 1H), 6.73 – 6.65 (m, 2H), 5.02 (s, 1H), 2.93 (s, 6H), 1.98 (s, 3H). ^{13}C{^{1}H} NMR (101 MHz, $CDCl_3$) *δ* 149.8, 142.1, 129.1, 129.0, 128.5, 128.4, 127.0, 112.6, 55.7, 40.7, 16.0. HRMS *m/z*: $[M+H]^+$ calcd for $C_{16}H_{20}NS$ 258.1316; found 258.1311.

1f[1] isolated as an off-white waxy solid (36.3 mg, 22%). ^{1}H NMR (400 MHz, $CDCl_3$) *δ* 7.32 – 7.21 (m, 2H), 7.21 – 7.16 (m, 1H), 7.16 – 7.10 (m, 2H), 7.04 – 6.95 (m, 4H), 6.73 – 6.63 (m, 4H), 5.39 (s, 1H), 2.92 (s, 12H). ^{13}C{^{1}H} NMR (101 MHz, $CDCl_3$) *δ* 149.1, 145.6, 133.0, 130.1, 129.5, 128.2, 125.9, 112.7, 55.1, 40.9.

1e[2] Isolated as a light brown oil (31.2 mg, 27%). ^{1}H NMR (400 MHz, $CDCl_3$) δ 7.45 – 7.37 (m, 2H), 7.37 – 7.30 (m, 2H), 7.30 – 7.16 (m, 3H), 6.74 – 6.66 (m, 2H), 5.78 (s, 1H), 2.93 (s, 6H), 2.14 (s, 1H). ^{13}C{^{1}H} NMR (101 MHz, $CDCl_3$) δ 150.3, 144.4, 132.1, 128.4, 127.9, 127.3, 126.5, 112.6, 76.1, 40.7.

(4-(dimethylamino)phenyl)(phenyl)methanol **(1e)** was also synthesized according to literature procedure.[2] ^{1}H NMR (400 MHz, $CDCl_3$) δ 7.43 – 7.37 (m, 2H), 7.37 – 7.30 (m, 2H), 7.29 – 7.24 (m, 1H), 7.24 – 7.19 (m, 1H), 6.73 – 6.68 (m, 2H), 5.78 (d, *J* = 2.9 Hz, 1H), 2.94 (s, 6H), 2.18 (d, *J* = 3.4 Hz, 1H). ^{13}C{^{1}H} NMR (101 MHz, $CDCl_3$) δ 150.3, 144.4, 132.1, 128.4, 127.9, 127.3, 126.5, 112.6, 76.1, 40.7.

Experimental Data for mechanistic investigation.

Table 5, Entry 1. Synthesis of *N,N*-dimethyl-4-((methylthio)(phenyl)methyl)aniline **(1d)** from **2b.** A vial was charged with 92.2 mg (phenylmethylene)bis(methylsulfane) **(2b)** (0.5 mmol) and 66.6 mg *N,N*-dimethylaniline **(1c)** (0.55mmol). 1 ml DCE was added, followed by BF_3OEt_2 (2.0 mmol, 0.25 ml) The vial was sealed and the mixture was heated to 80ºC. After 16h the mixture was cooled to 0ºC and 0.2 ml water was added. The mixture was poured into 10 ml Sat. Na_2CO_3 and extracted with 3x20 ml DCM. The organics were pooled, dried over Na_2SO_4, filtered and concentrated under reduced pressure. The resulting crude was purified over silica using 2-5% EtOAc in *i*-Hexane. Isolated as white solid (38.7 mg, 30%). ^{1}H NMR (400 MHz, $CDCl_3$) *δ* 7.47 – 7.40 (m, 2H), 7.36 – 7.25 (m, 4H), 7.25 – 7.18 (m, 1H), 6.73 – 6.65 (m, 2H), 5.02 (s, 1H), 2.93 (s, 6H), 1.98 (s, 3H). ^{13}C{^{1}H} NMR (101 MHz, $CDCl_3$) *δ* 149.8, 142.1, 129.1, 129.0, 128.5, 128.4, 127.0, 112.6, 55.7, 40.7, 16.0. HRMS *m/z*: $[M+H]^+$ calcd for $C_{16}H_{20}NS$ 258.1316; found 258.1311

Table 5, Entry 4. Synthesis of *N,N*-diethyl-4-((methylthio)(phenyl)methyl)aniline **(2d)** from **1e.** A vial was charged with 114.0 mg (4-(dimethylamino)phenyl)(phenyl)methanol **(1e)** (0.5 mmol). 1 ml DCE was added, followed by 4 equiv. (2 mmol, 0.21 ml) BF_3SMe_2. The vial was sealed and the mixture was heated to 80ºC. After 16h the mixture was cooled to 0ºC and 0.2 ml water was added. The mixture was poured into 10 ml Sat. Na_2CO_3 and extracted with 3x20 ml DCM. The organics were pooled, dried over Na_2SO_4, filtered and concentrated under reduced pressure. The resulting crude was purified over silica using 2-5% EtOAc in *i*-Hexane. Isolated as a white solid (113.7 mg, 88%). ^{1}H NMR (400 MHz, $CDCl_3$) *δ* 7.47 – 7.40 (m, 2H), 7.36 – 7.25 (m, 4H), 7.25 – 7.18 (m, 1H), 6.73 – 6.65 (m, 2H), 5.02 (s, 1H), 2.93 (s, 6H), 1.98 (s, 3H). ^{13}C{^{1}H} NMR (101 MHz, $CDCl_3$) *δ* 149.8, 142.1, 129.1, 129.0, 128.5, 128.4, 127.0, 112.6, 55.7, 40.7, 16.0. HRMS *m/z*: $[M+H]^+$ calcd for $C_{16}H_{20}NS$ 258.1316; found 258.1311

Synthesis of 1-methyl-4-phenylpiperazine (3c).

1-methyl-4-phenylpiperazine **(3c)** was synthesized according to literature procedure.[3] ^{1}H NMR (400 MHz, $CDCl_3$) δ 7.31 – 7.22 (m, 2H), 6.98 – 6.90 (m, 2H), 6.86 (tt, *J* = 7.3, 1.1 Hz, 1H), 3.25 – 3.18 (m, 4H), 2.62 – 2.54 (m, 4H), 2.35 (s, 3H). $^{13}C\{^{1}H\}$ NMR (101 MHz, $CDCl_3$) δ 151.4, 129.2, 119.8, 116.2, 55.3, 49.2, 46.3.

NMR Spectra

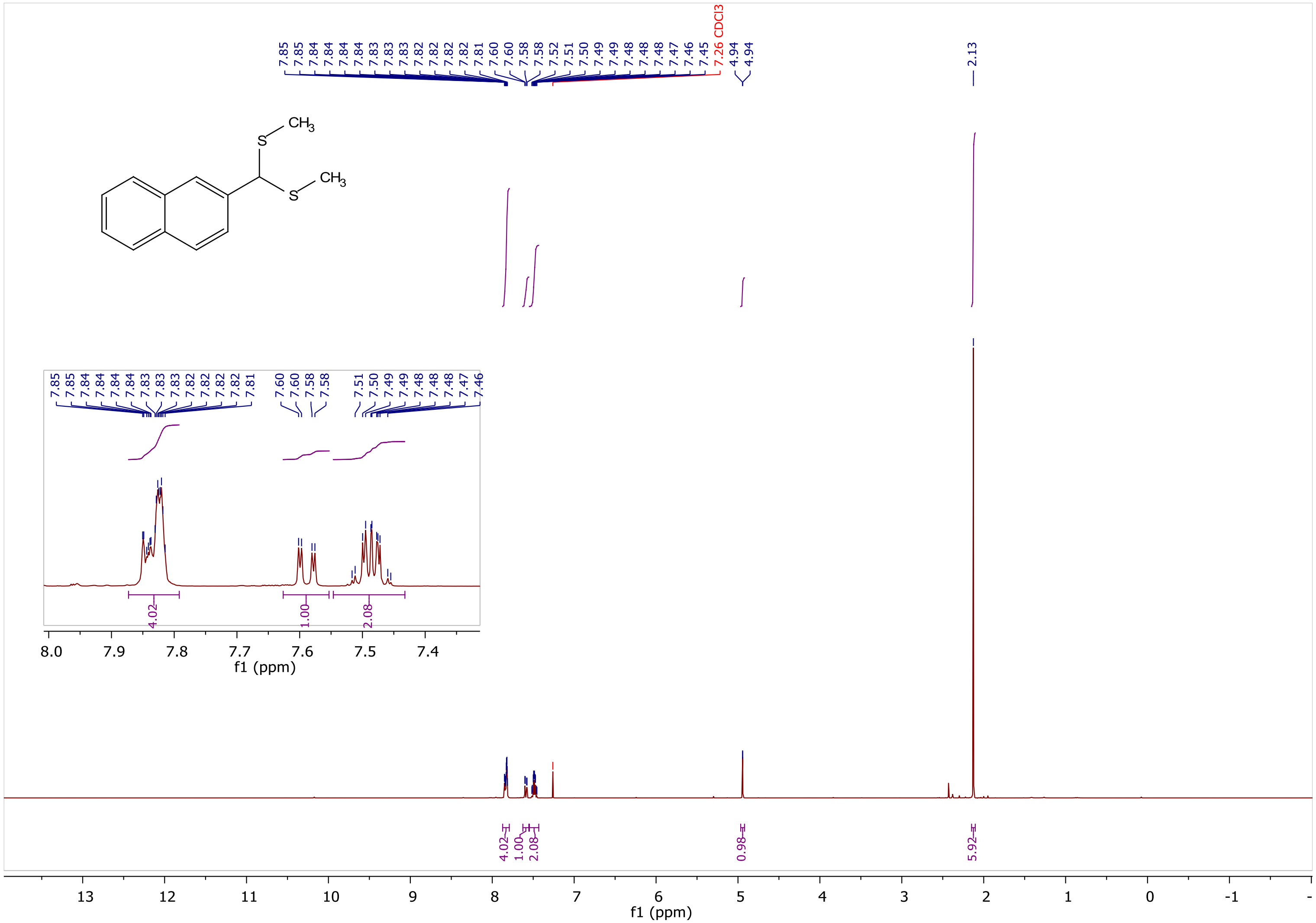

Figure S1. ^{1}H-NMR (400 MHz $CDCl_3$) of compound **1b.**

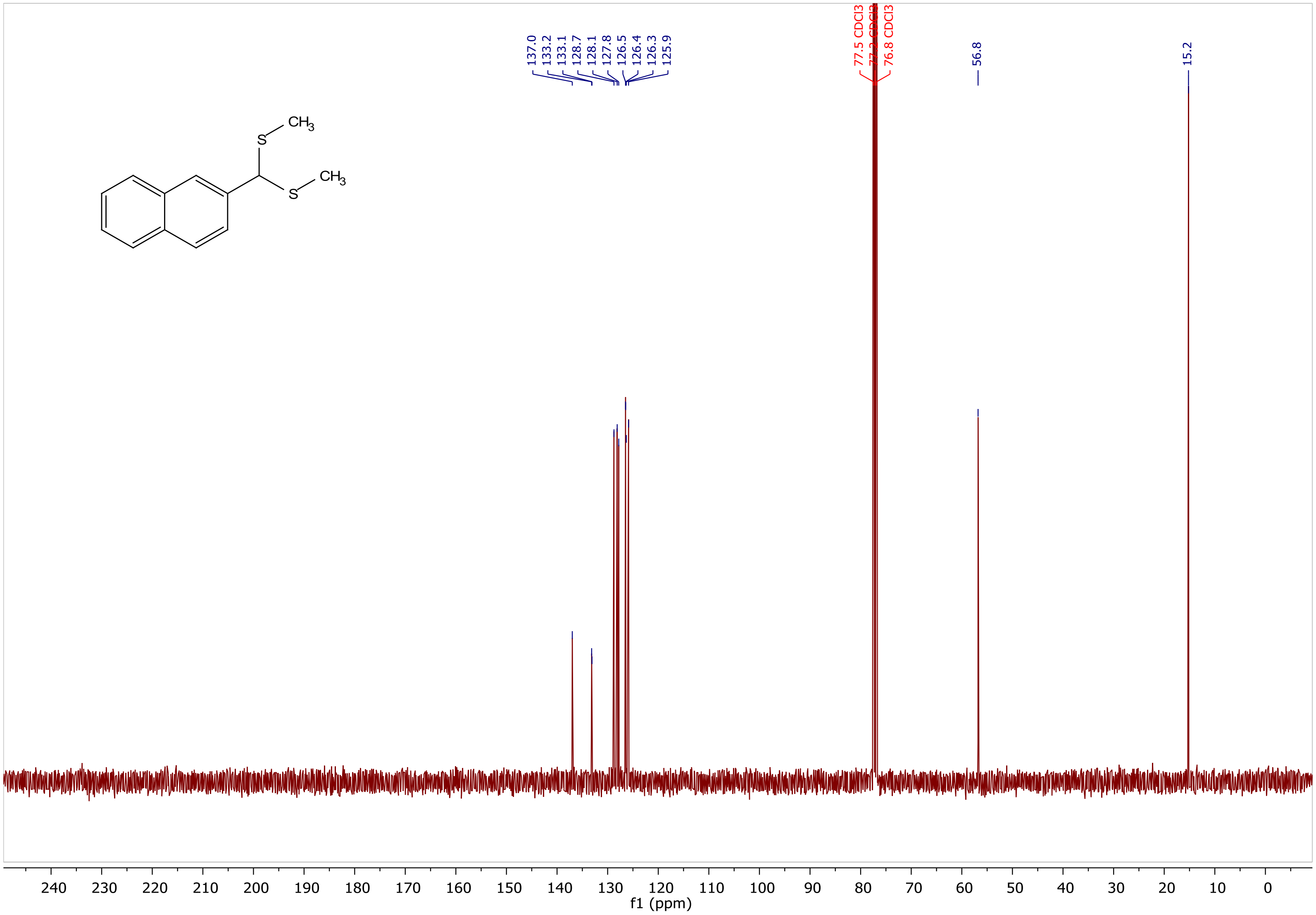

Figure S2. $^{13}C\{^{1}H\}$-NMR (101 MHz, $CDCl_3$) of compound **1b**.

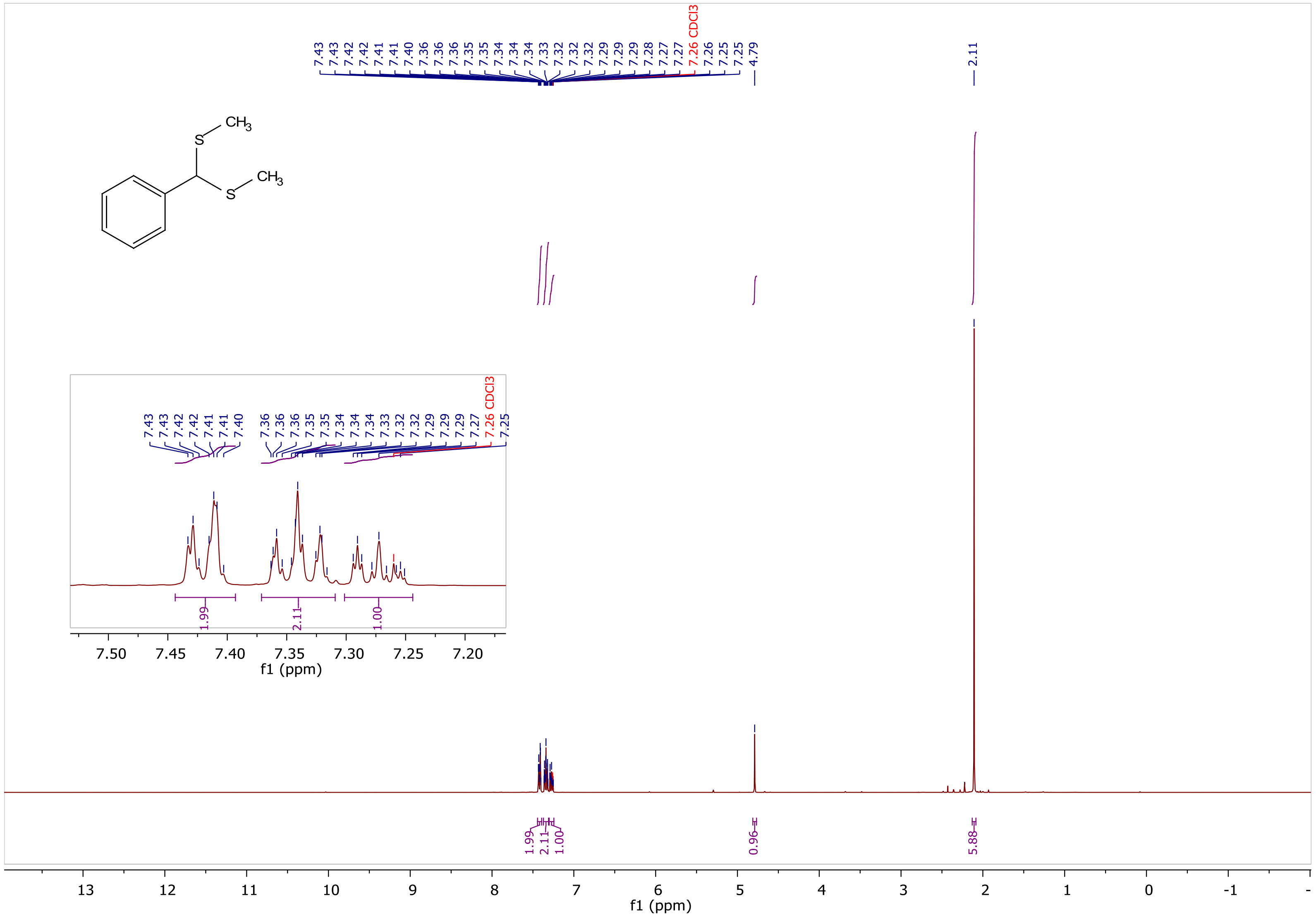

Figure S3. ^{1}H-NMR (400 MHz $CDCl_3$) of compound **2b.**

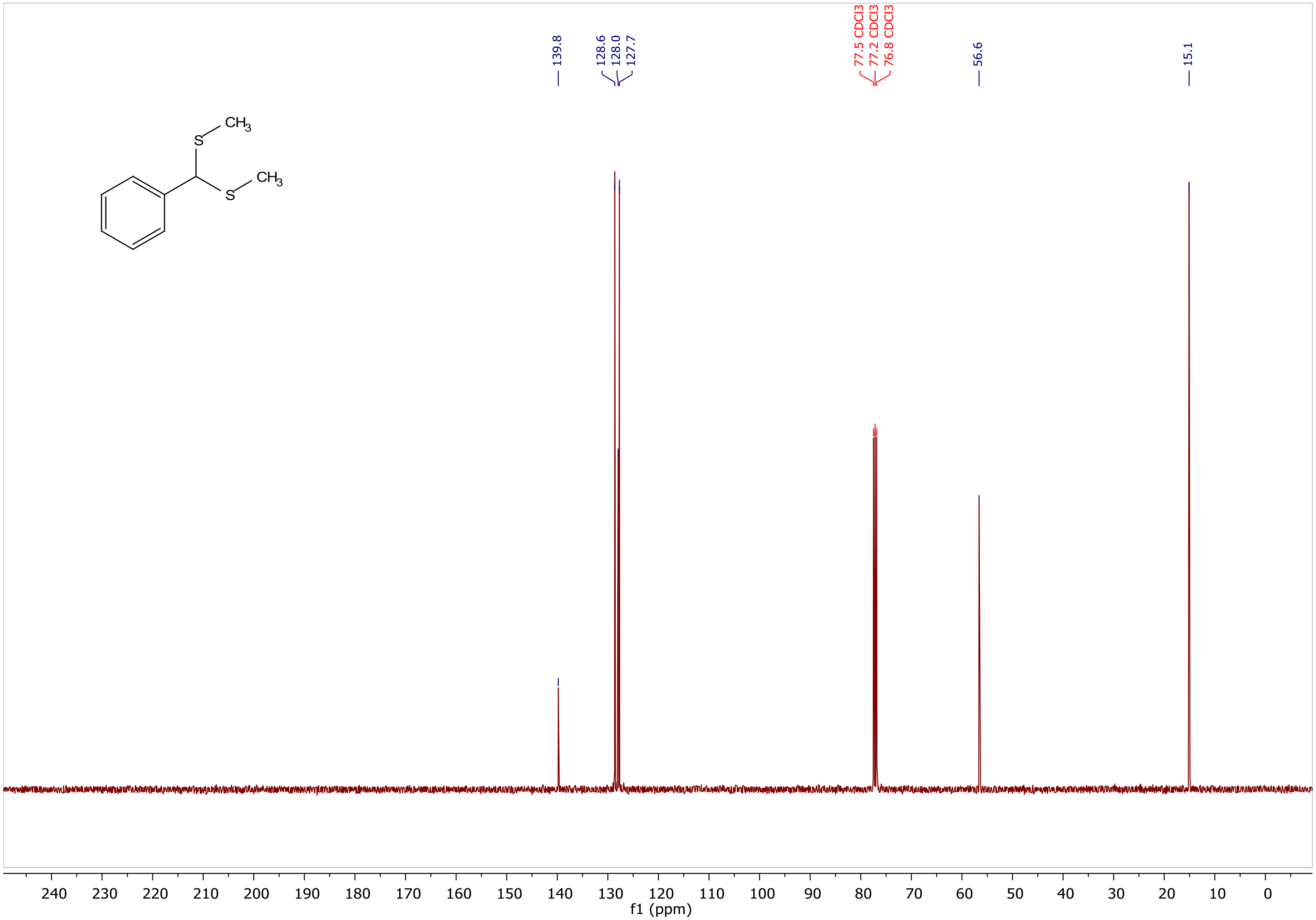

Figure S4. $^{13}C\{^{1}H\}$-NMR (101 MHz, $CDCl_3$) of compound **2b**.

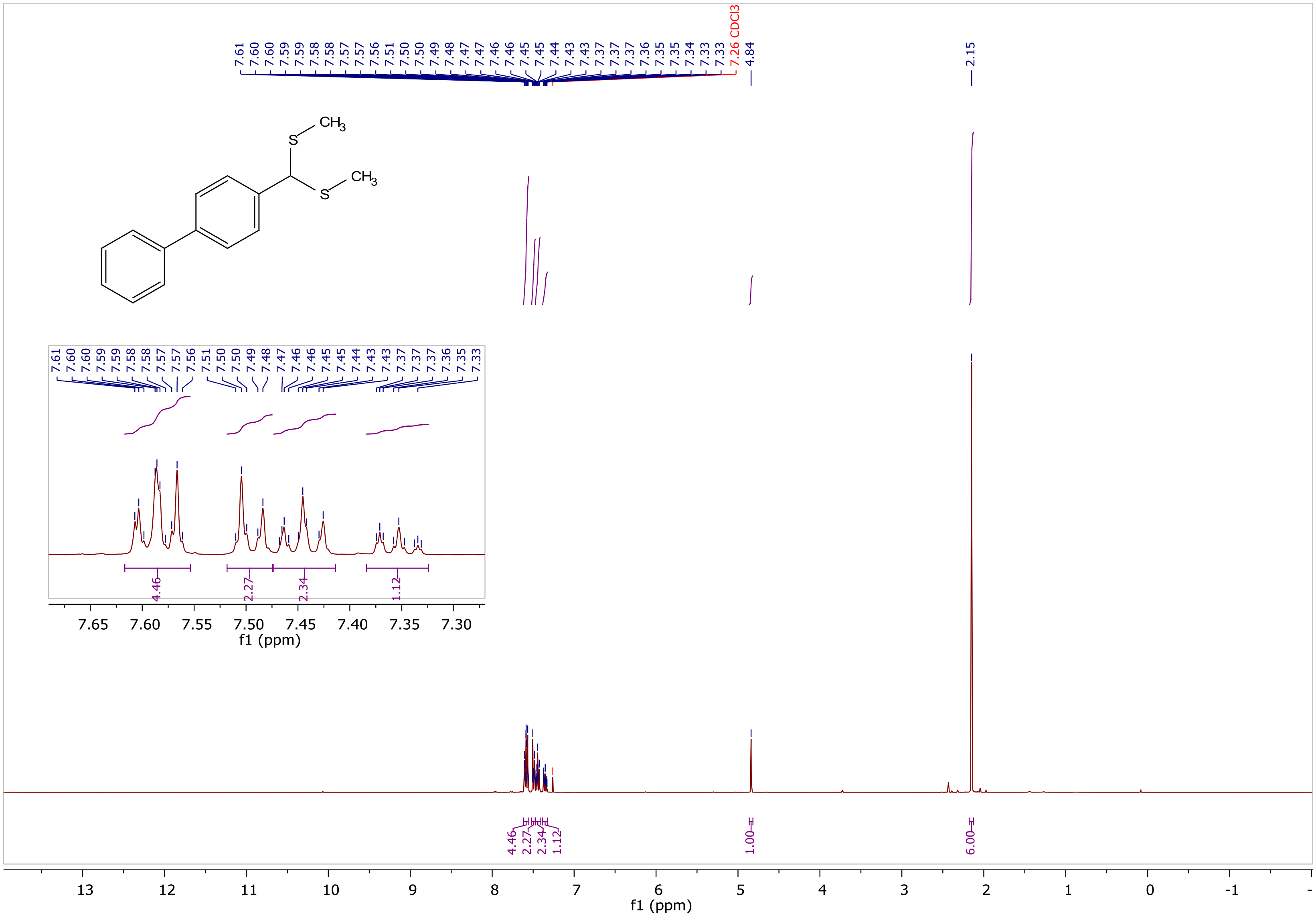

Figure S5. ^{1}H-NMR (400 MHz $CDCl_3$) of compound **3b.**

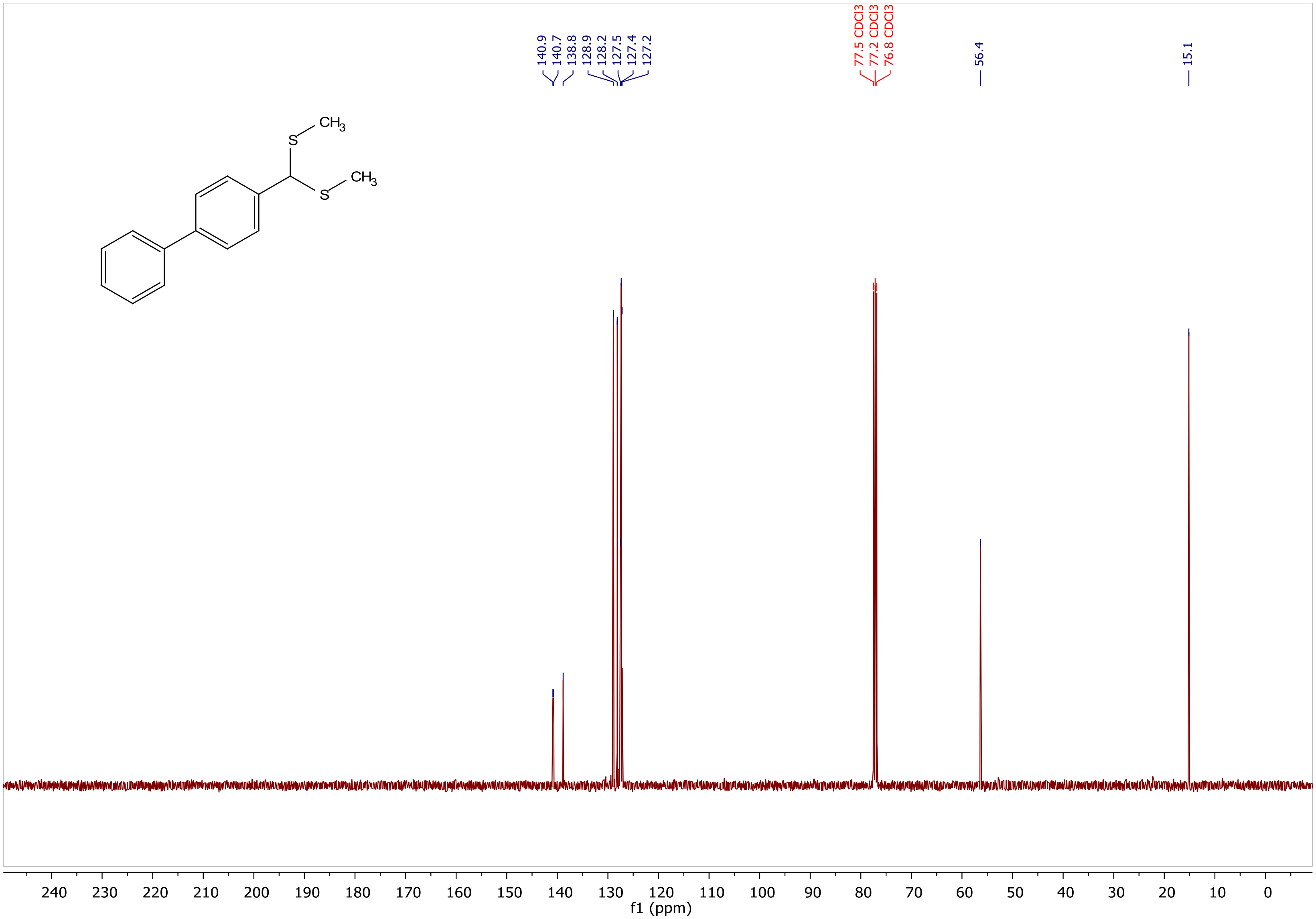

Figure S6. $^{13}C\{^{1}H\}$-NMR (101 MHz, $CDCl_3$) of compound **3b**.

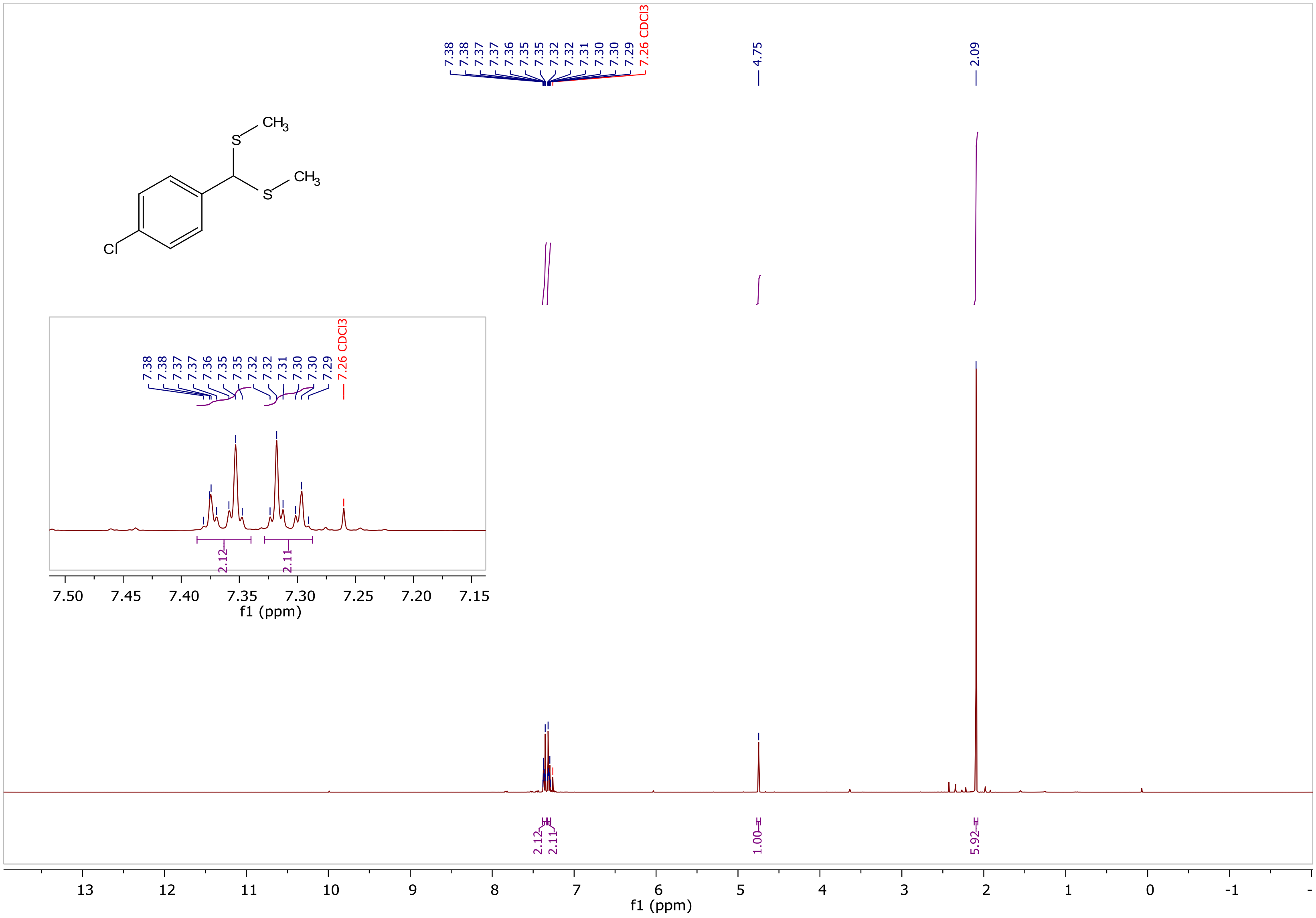

Figure S7. 1H-NMR (400 MHz $CDCl_3$) of compound **4b**.

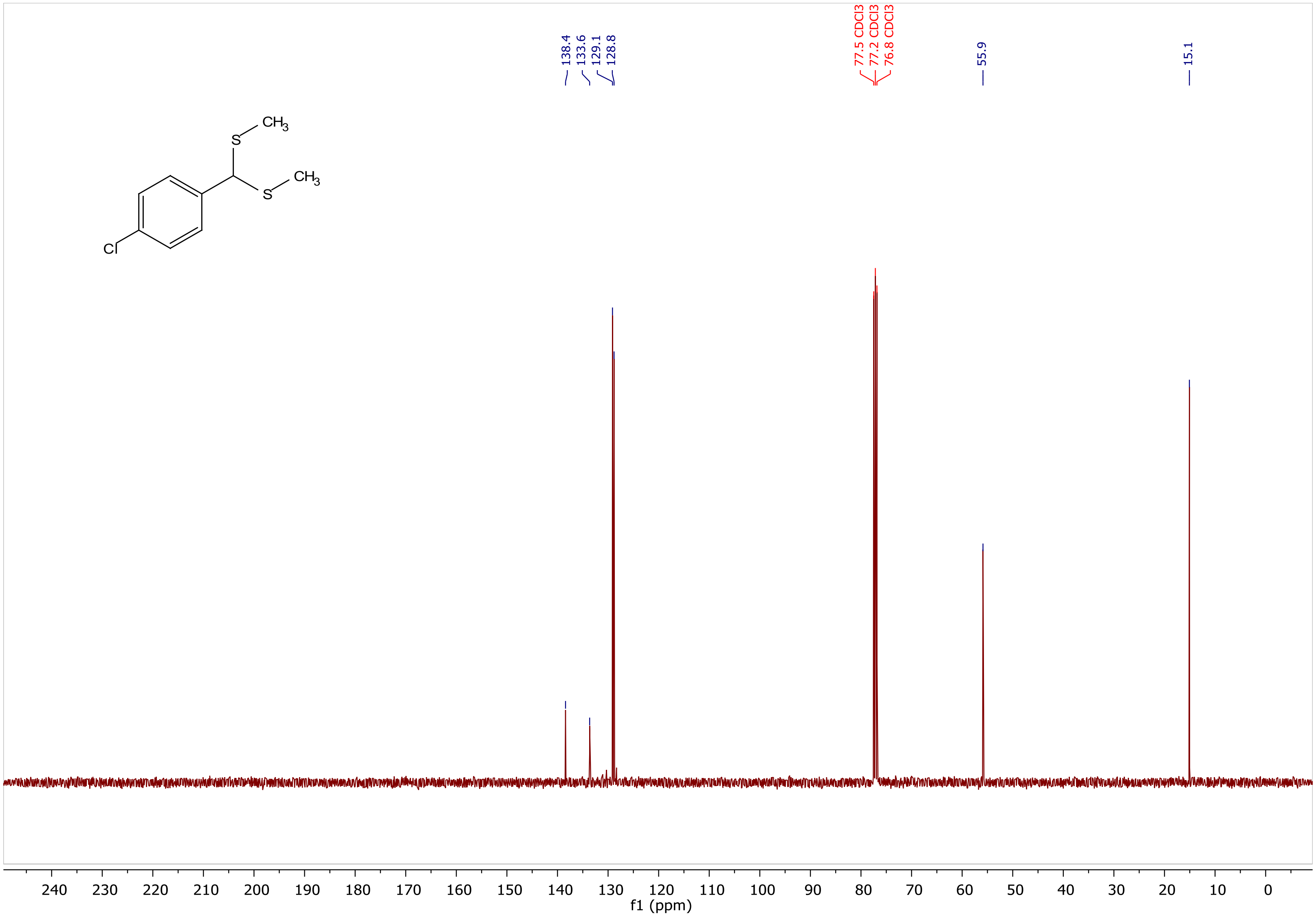

Figure S8. $^{13}C\{^1H\}$-NMR (101 MHz, $CDCl_3$) of compound **4b.**

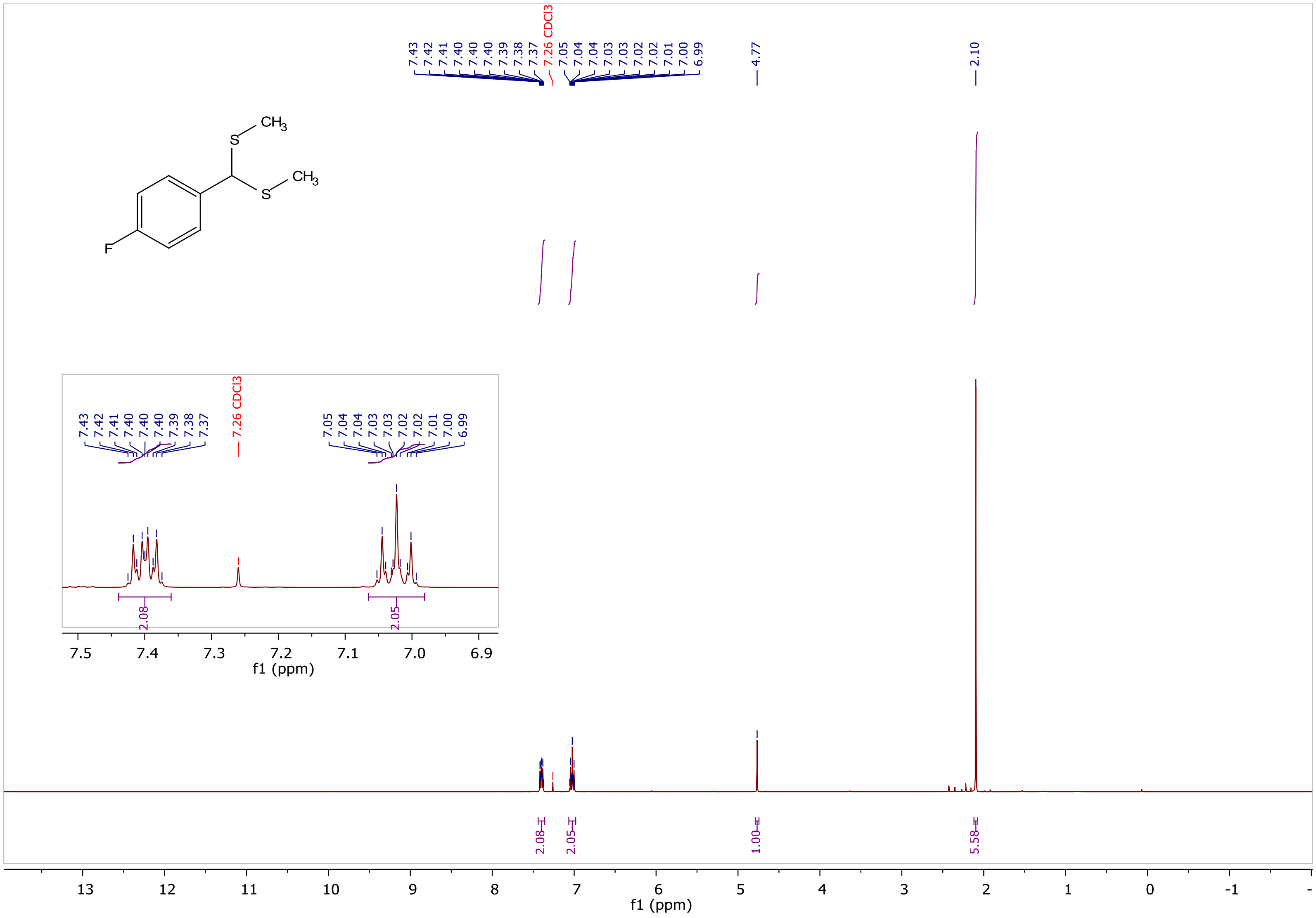

Figure S9. ^{1}H-NMR (400 MHz $CDCl_3$) of compound **5b.**

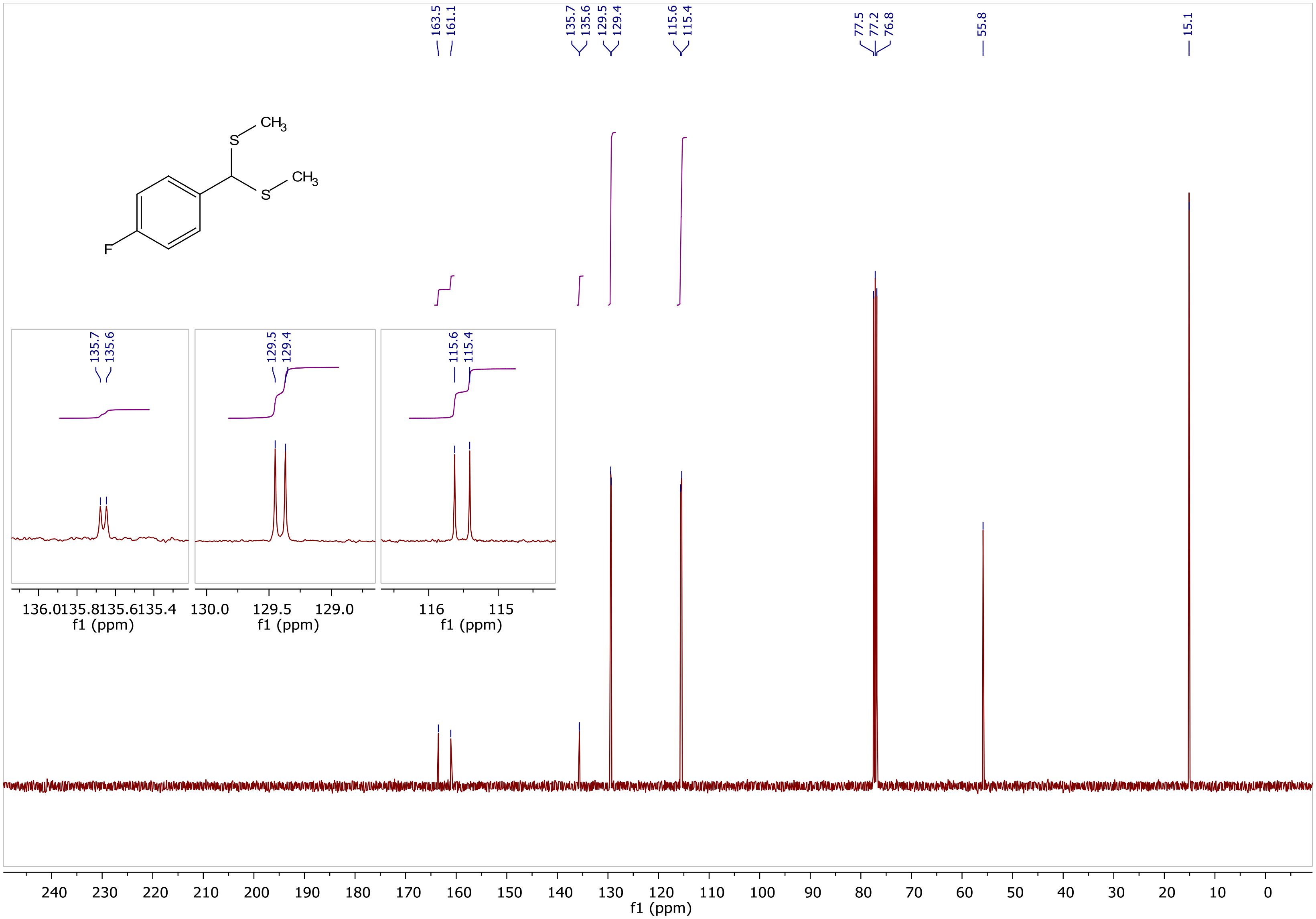

Figure S10. $^{13}C\{^{1}H\}$-NMR (101 MHz, $CDCl_3$) of compound **5b.**

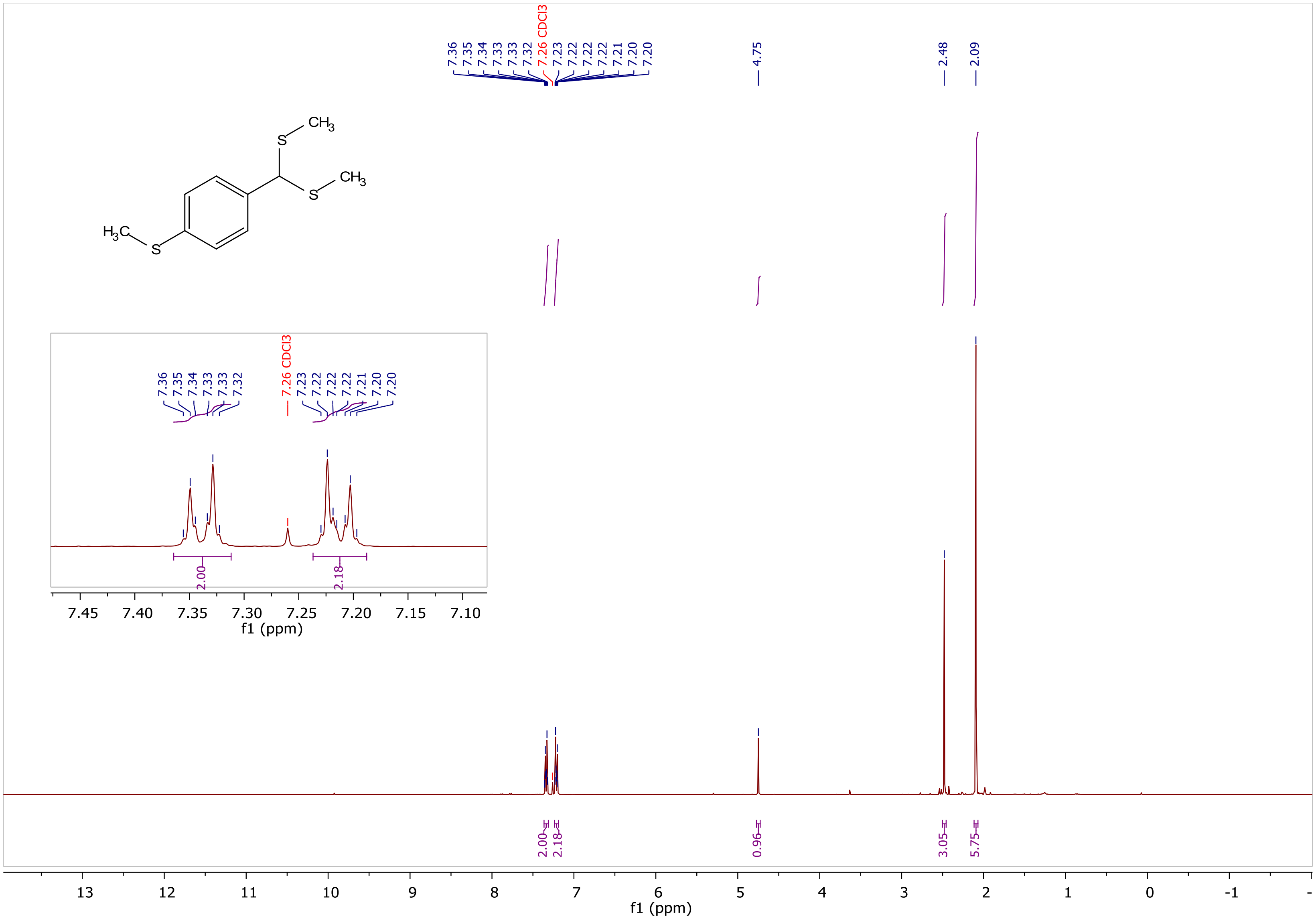

Figure S11. ^{1}H-NMR (400 MHz $CDCl_3$) of compound **6b.**

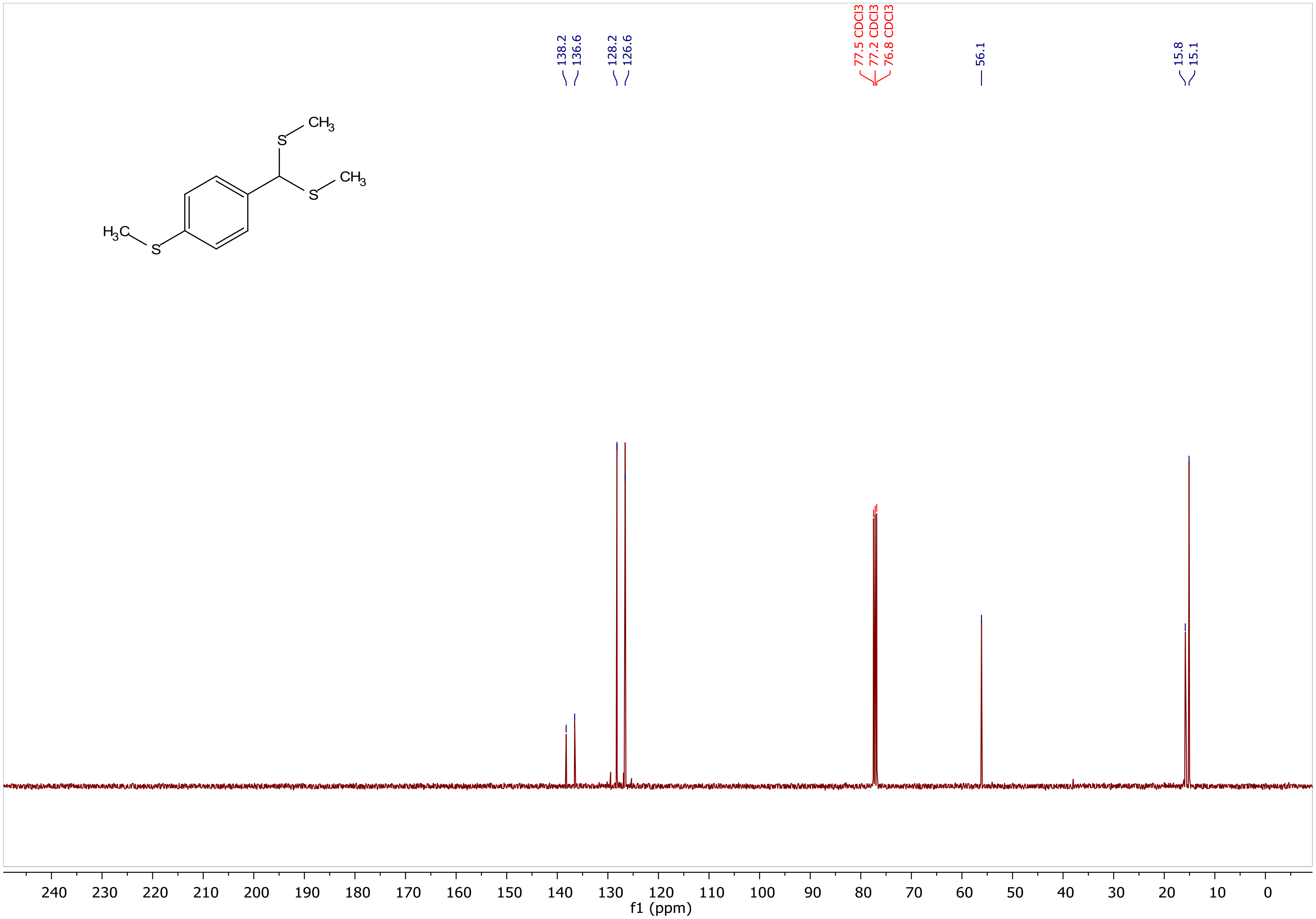

Figure S12. $^{13}C\{^1H\}$-NMR (101 MHz, $CDCl_3$) of compound **6b.**

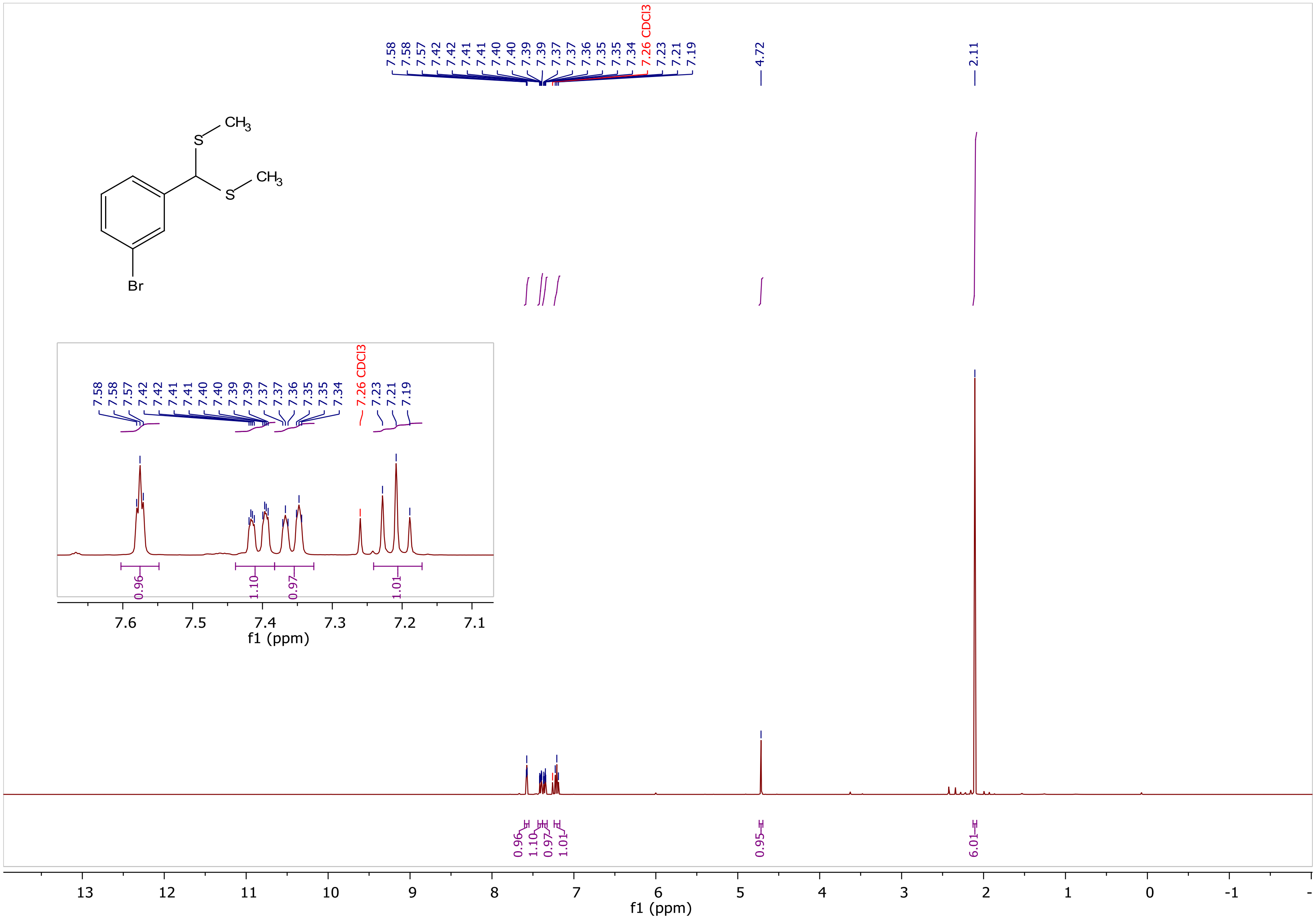

Figure S13. ^{1}H-NMR (400 MHz $CDCl_3$) of compound **7b.**

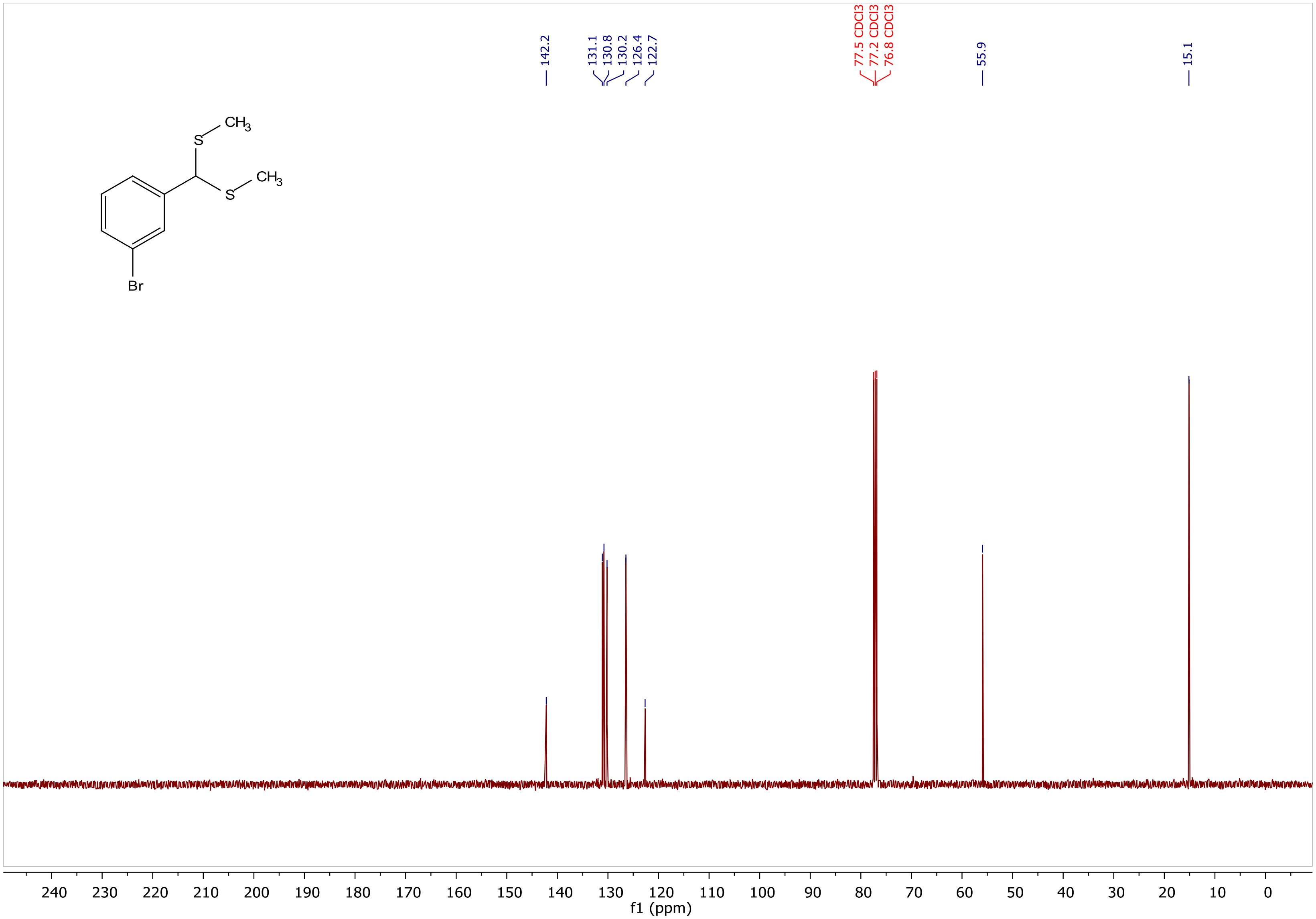

Figure S14. $^{13}C\{^{1}H\}$-NMR (101 MHz, $CDCl_3$) of compound **7b.**

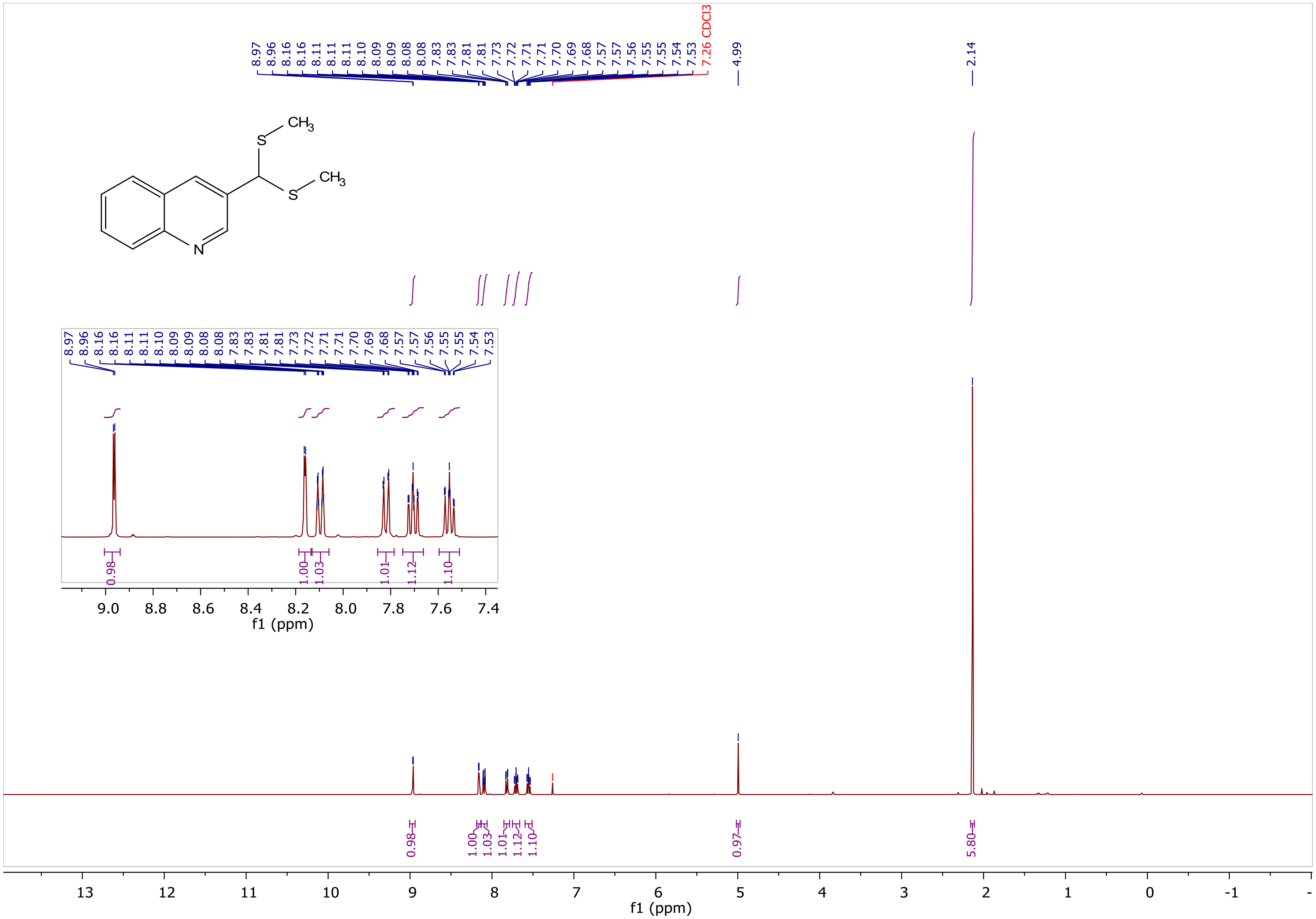

Figure S15. ^{1}H-NMR (400 MHz $CDCl_3$) of compound **8b.**

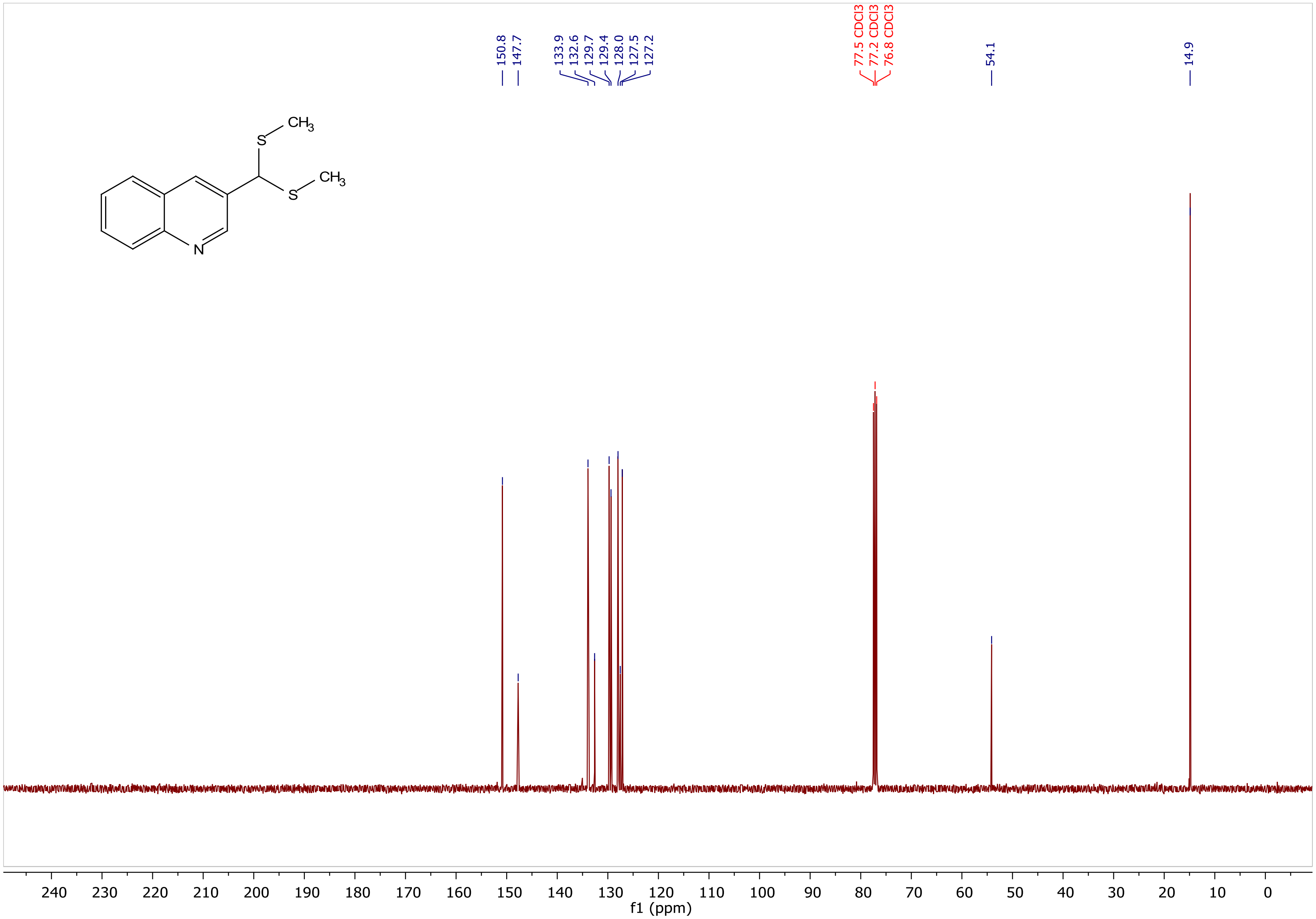

Figure S16. $^{13}C\{^1H\}$-NMR (101 MHz, $CDCl_3$) of compound **8b.**

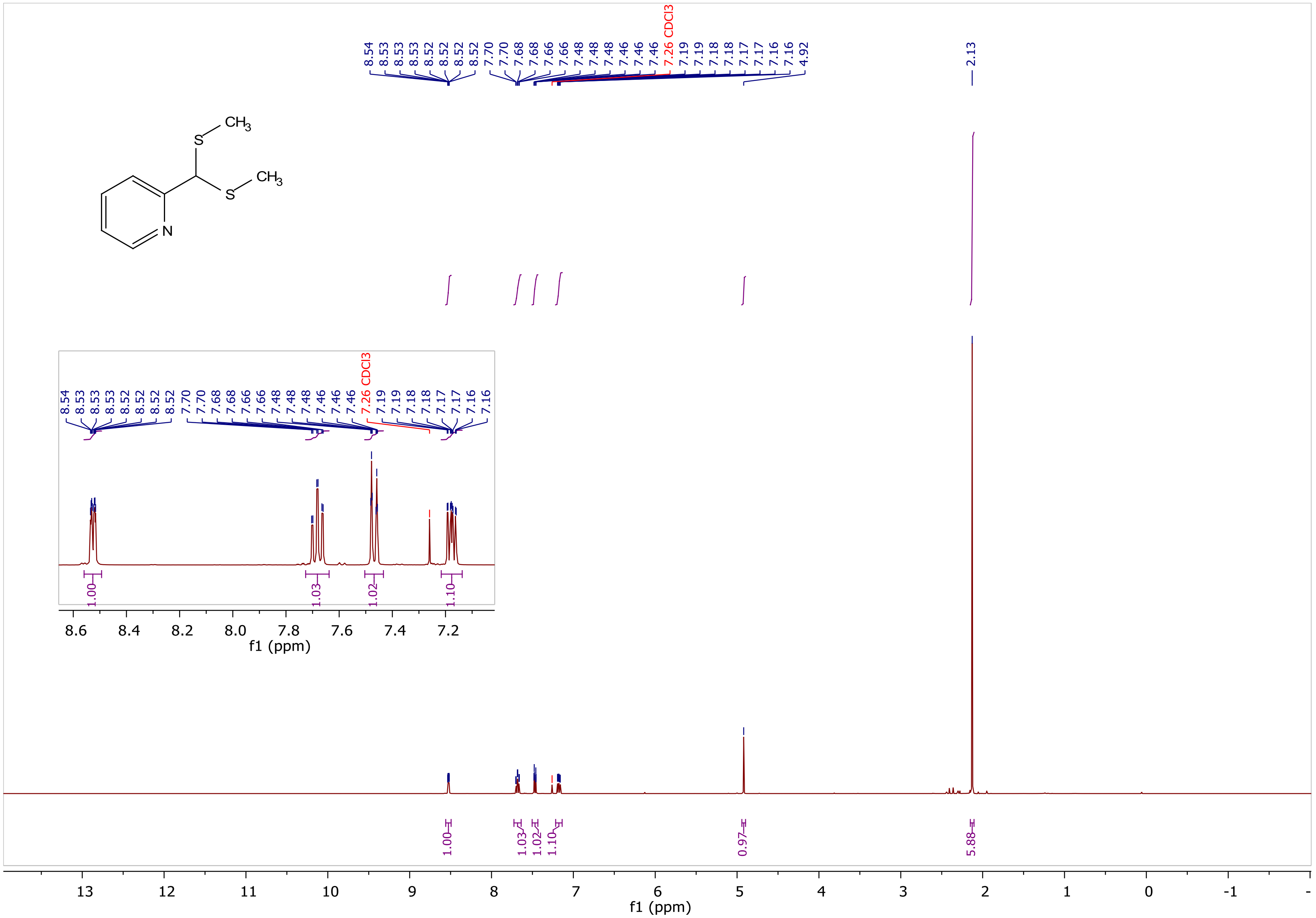

Figure S17. ^{1}H-NMR (400 MHz $CDCl_3$) of compound **9b.**

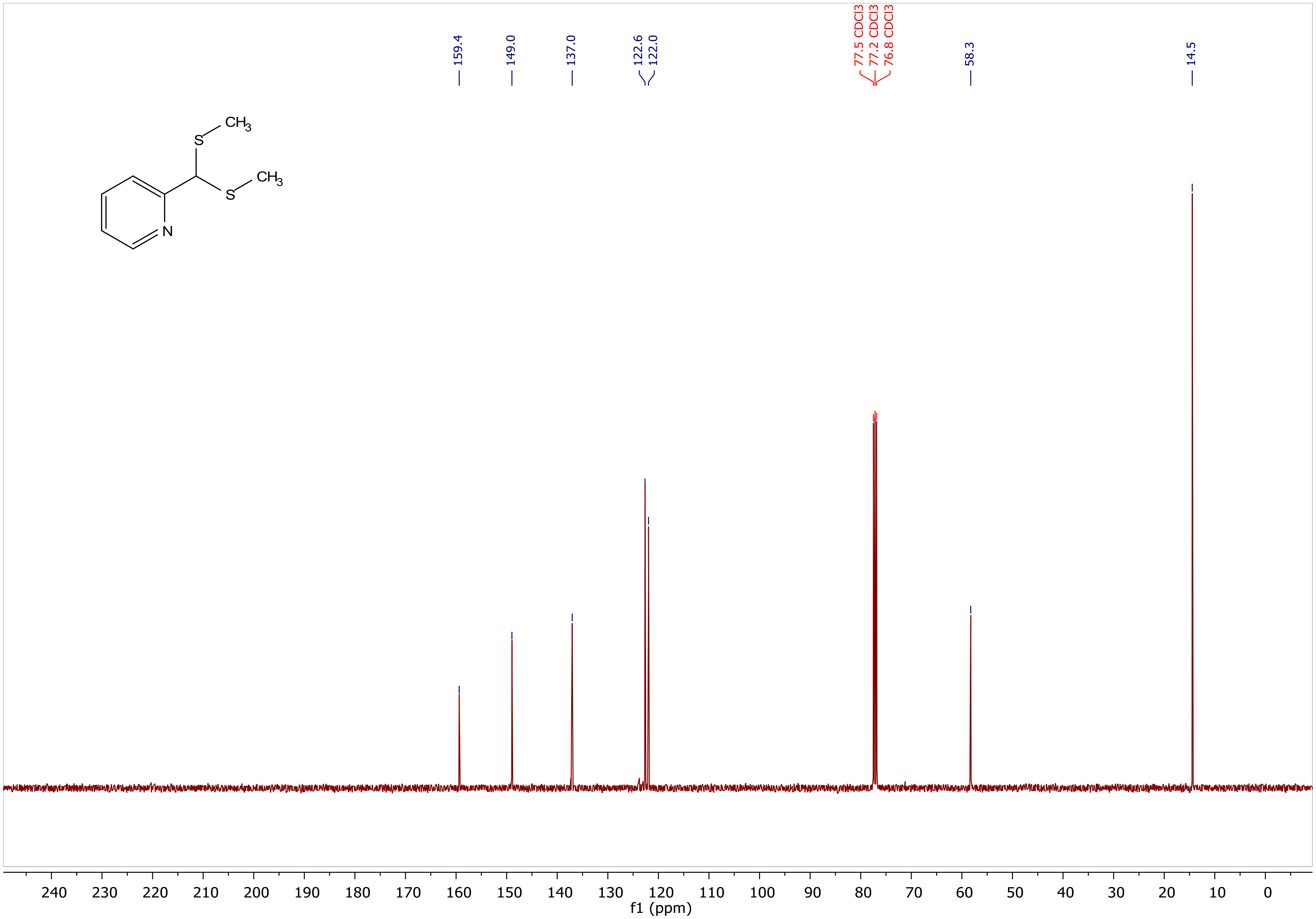

Figure S18. $^{13}C\{^1H\}$-NMR (101 MHz, $CDCl_3$) of compound **9b.**

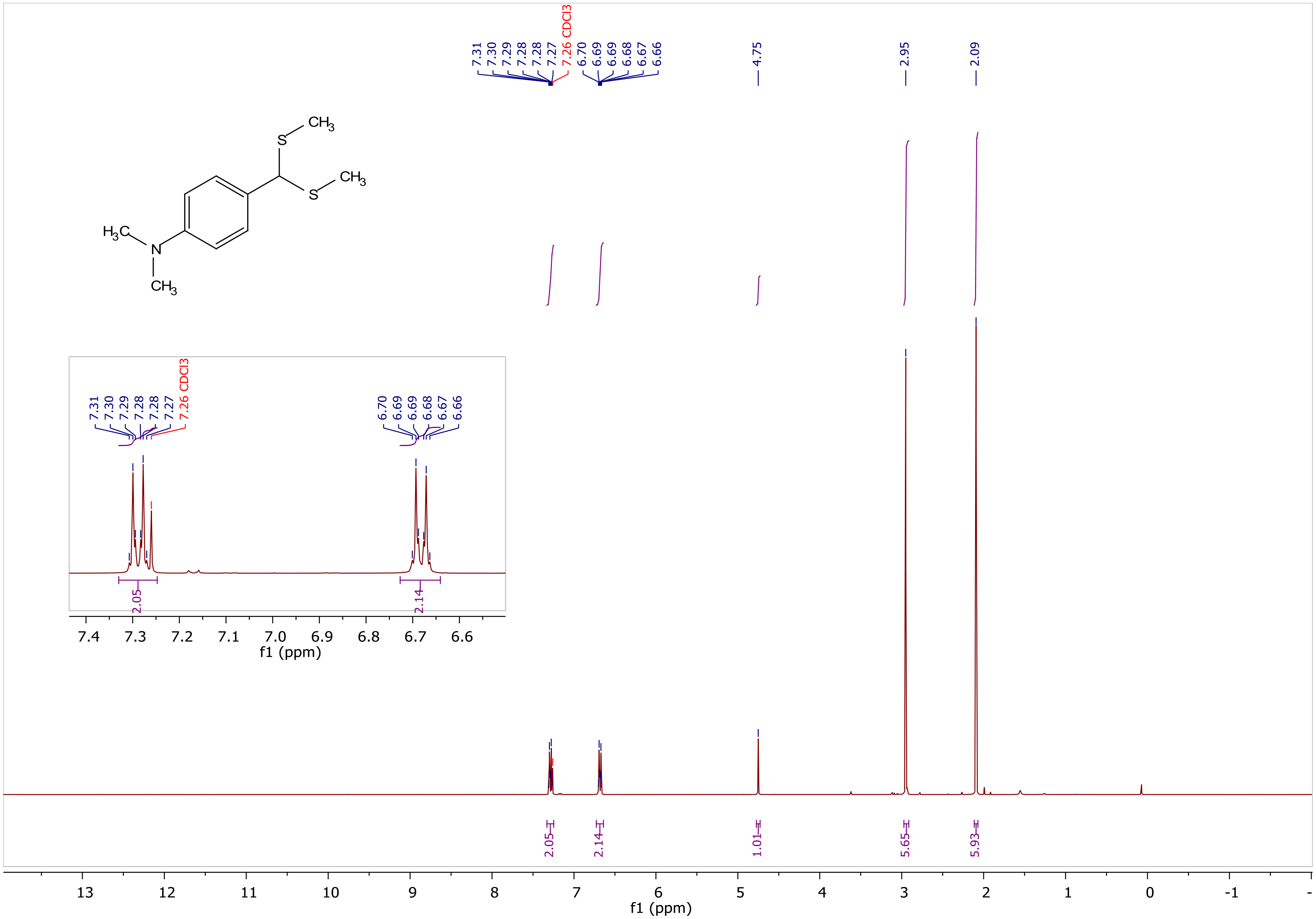

Figure S19. ^{1}H-NMR (400 MHz $CDCl_3$) of compound **10b.**

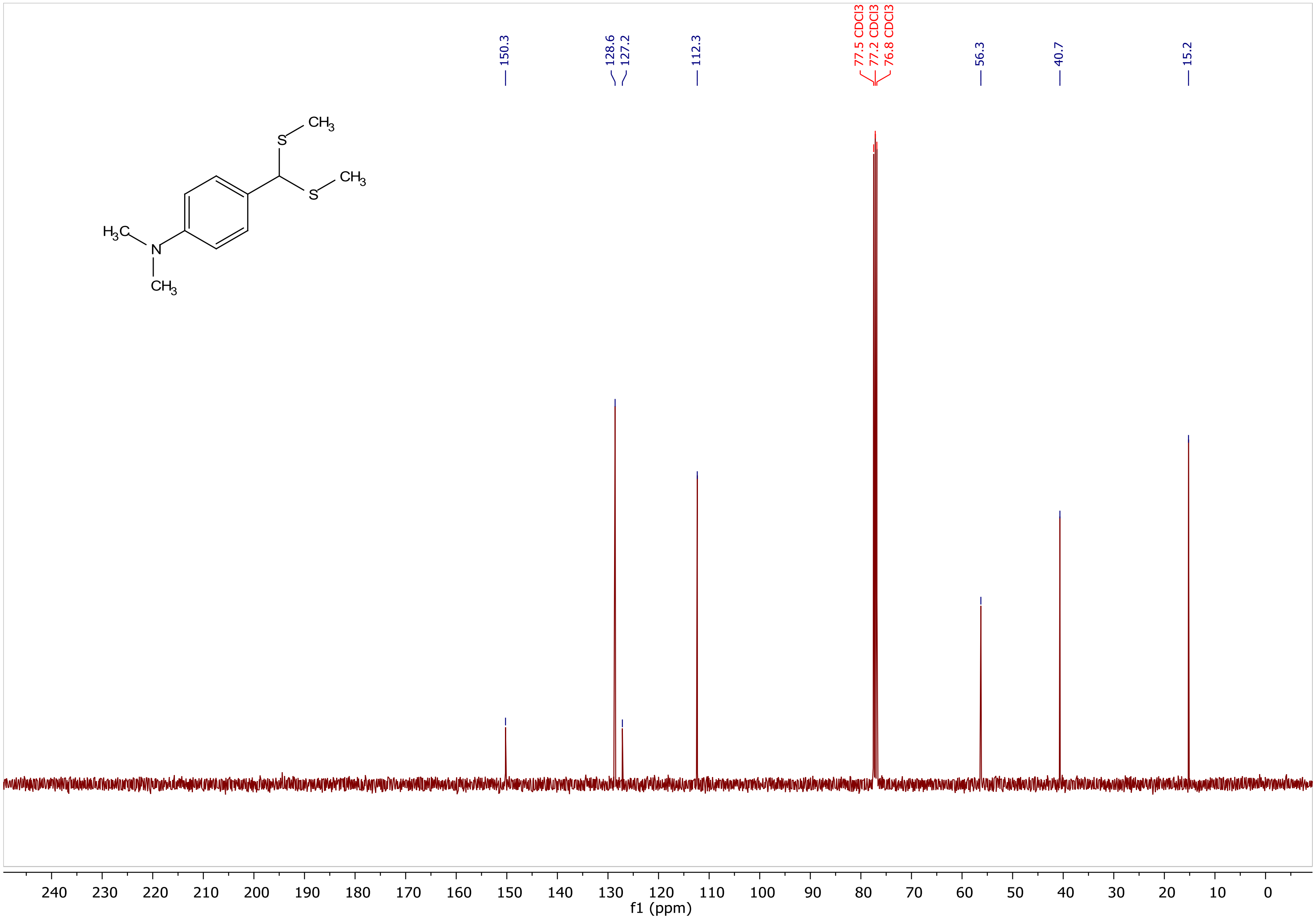

Figure S20. $^{13}C\{^{1}H\}$-NMR (101 MHz, $CDCl_3$) of compound **10b.**

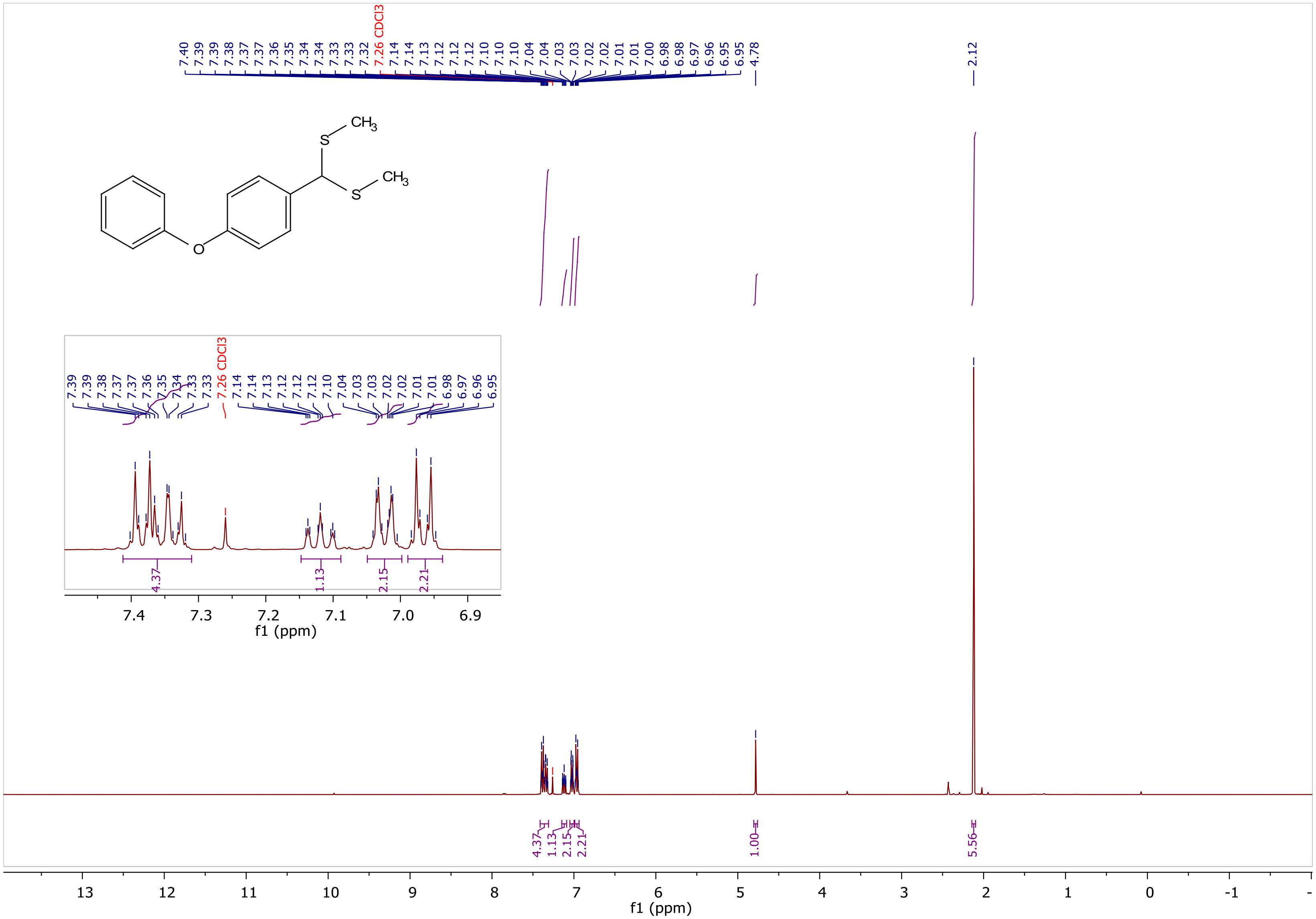

Figure S21. ^{1}H-NMR (400 MHz $CDCl_3$) of compound **11b.**

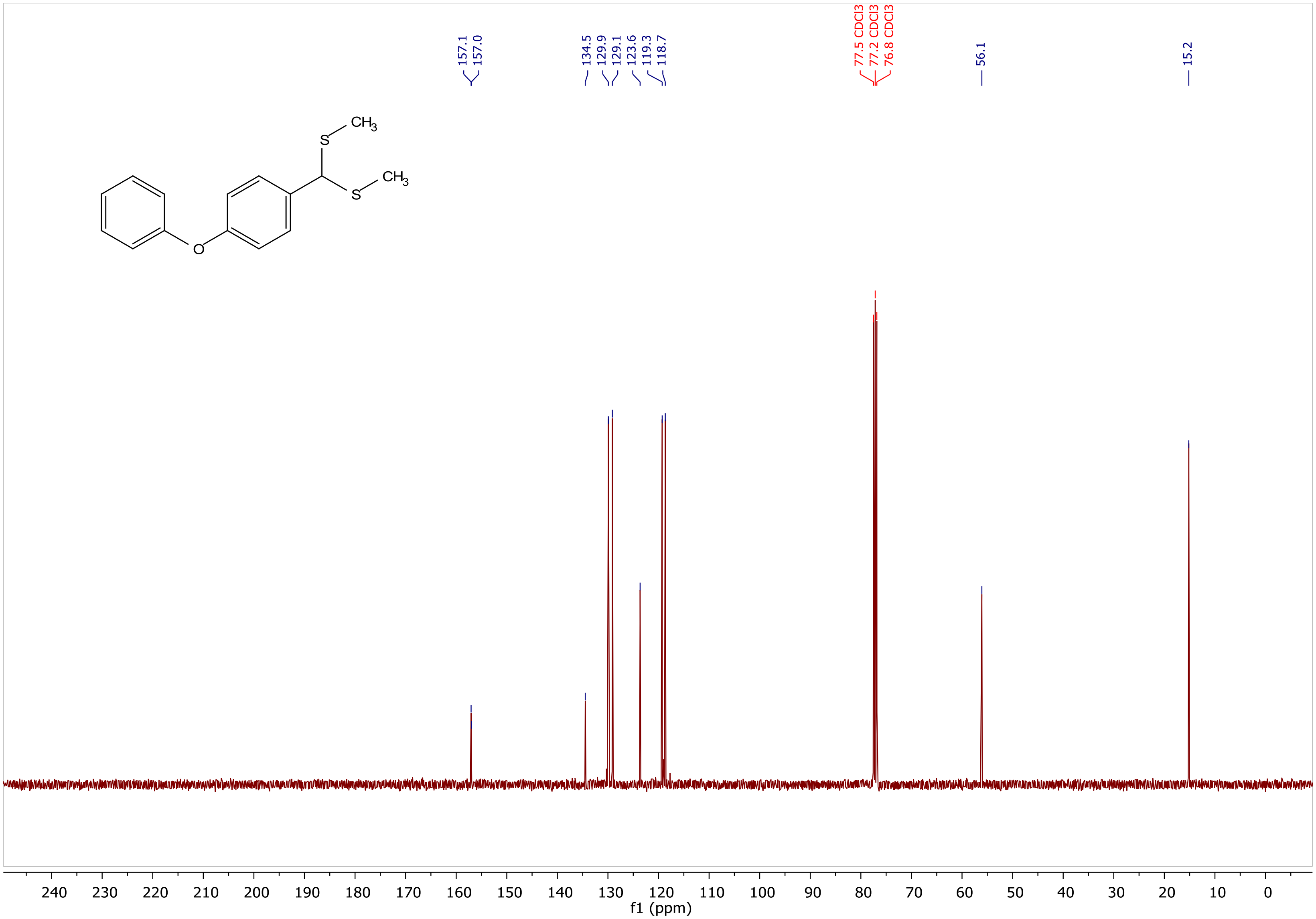

Figure S22. $^{13}C\{^{1}H\}$-NMR (101 MHz, $CDCl_3$) of compound **11b.**

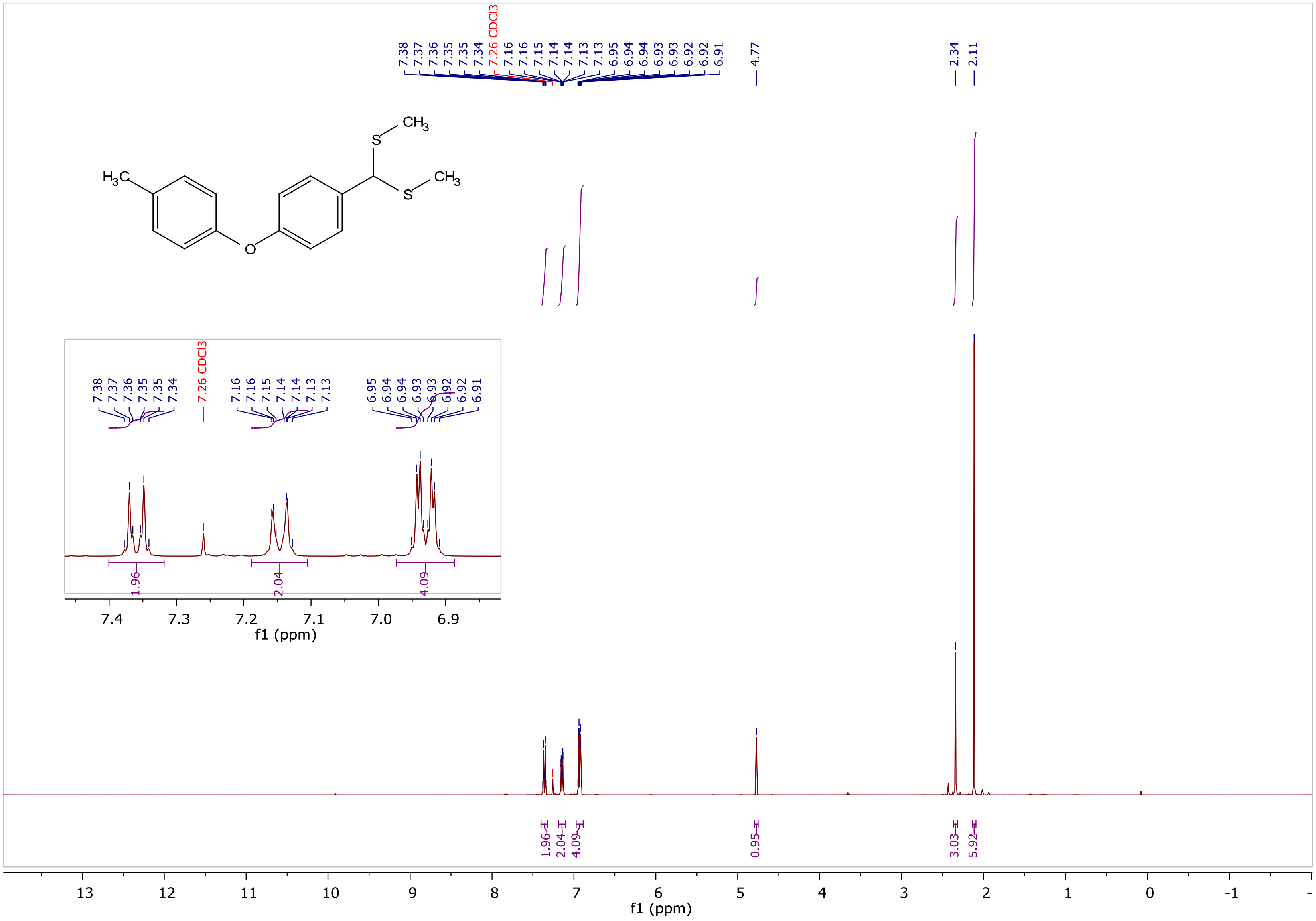

Figure S23. ^{1}H-NMR (400 MHz $CDCl_3$) of compound **12b.**

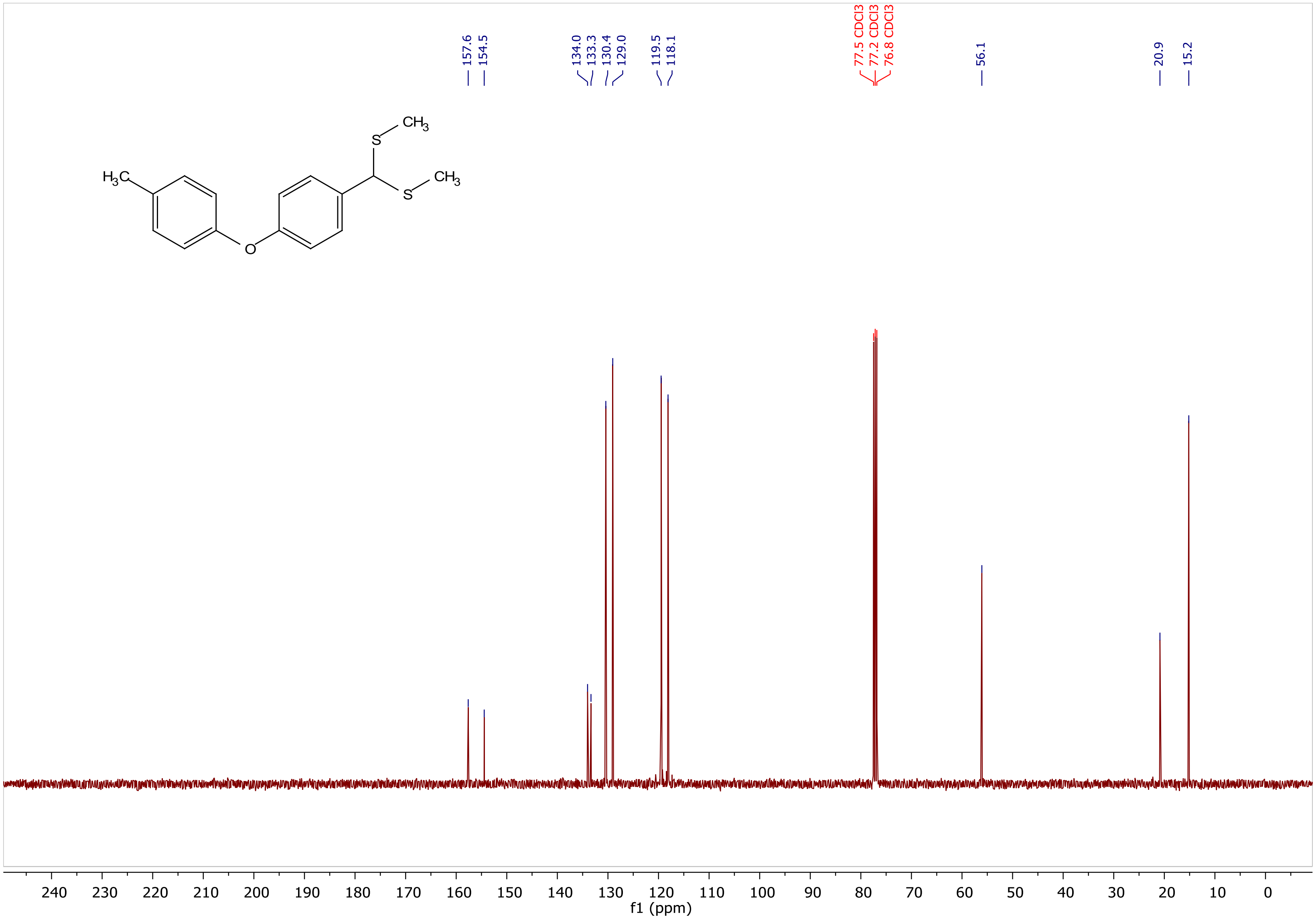

Figure S24. $^{13}C\{^{1}H\}$-NMR (101 MHz, $CDCl_3$) of compound **12b.**

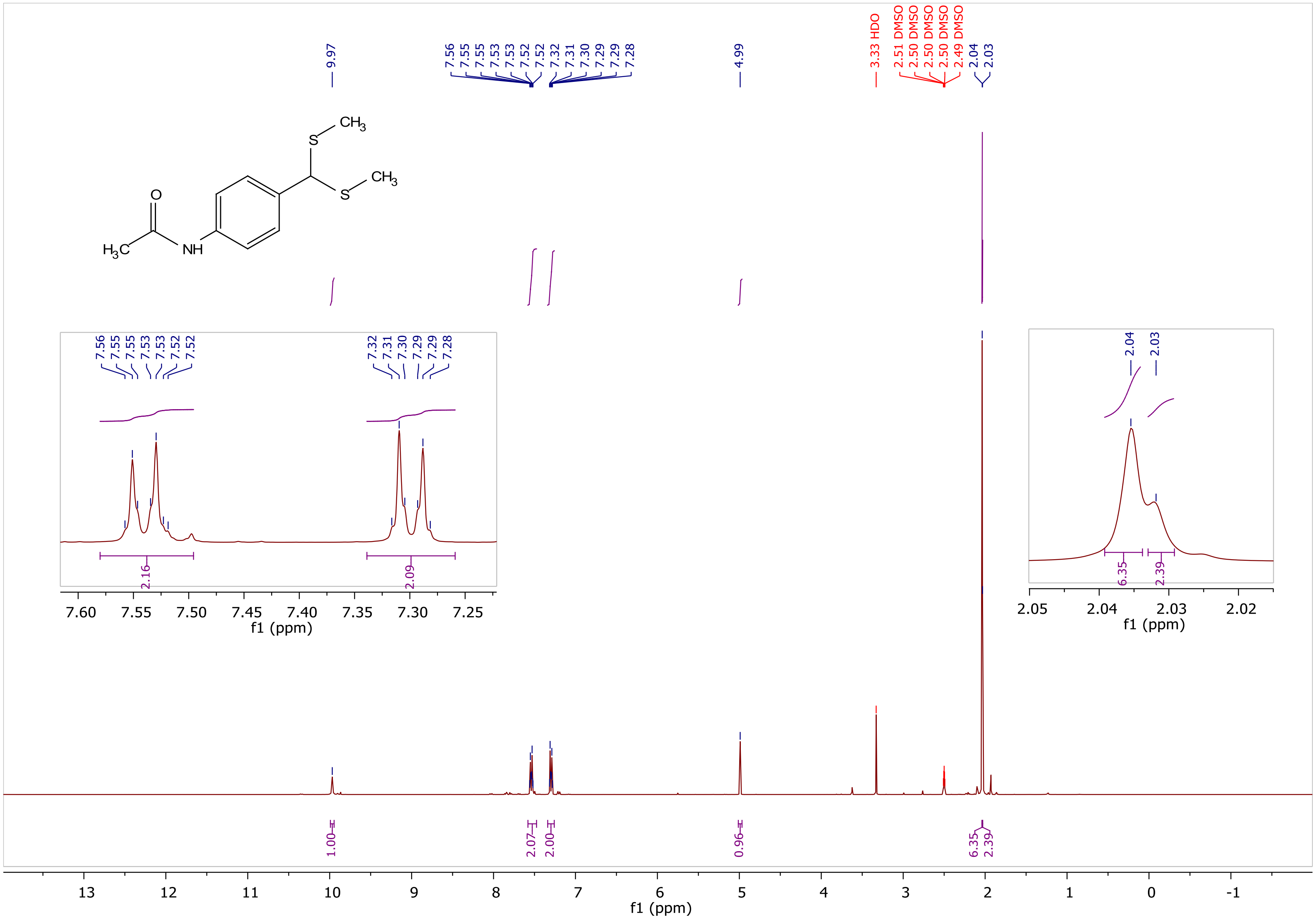

Figure S25. ^{1}H-NMR (400 MHz DMSO-d_6) of compound **13b.**

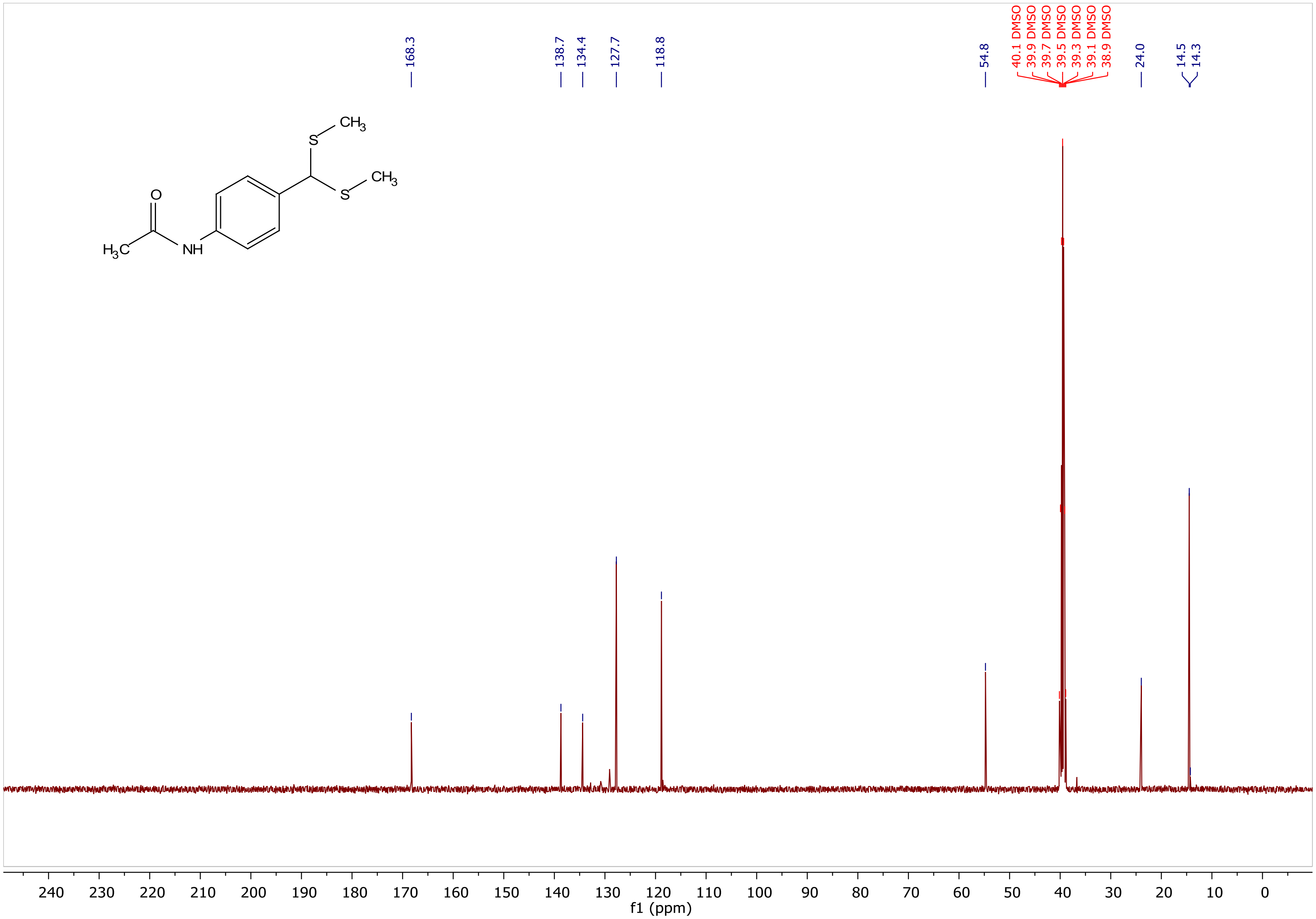

Figure S26. $^{13}C\{^1H\}$-NMR (101 MHz, DMSO-d$_6$) of compound **13b.**

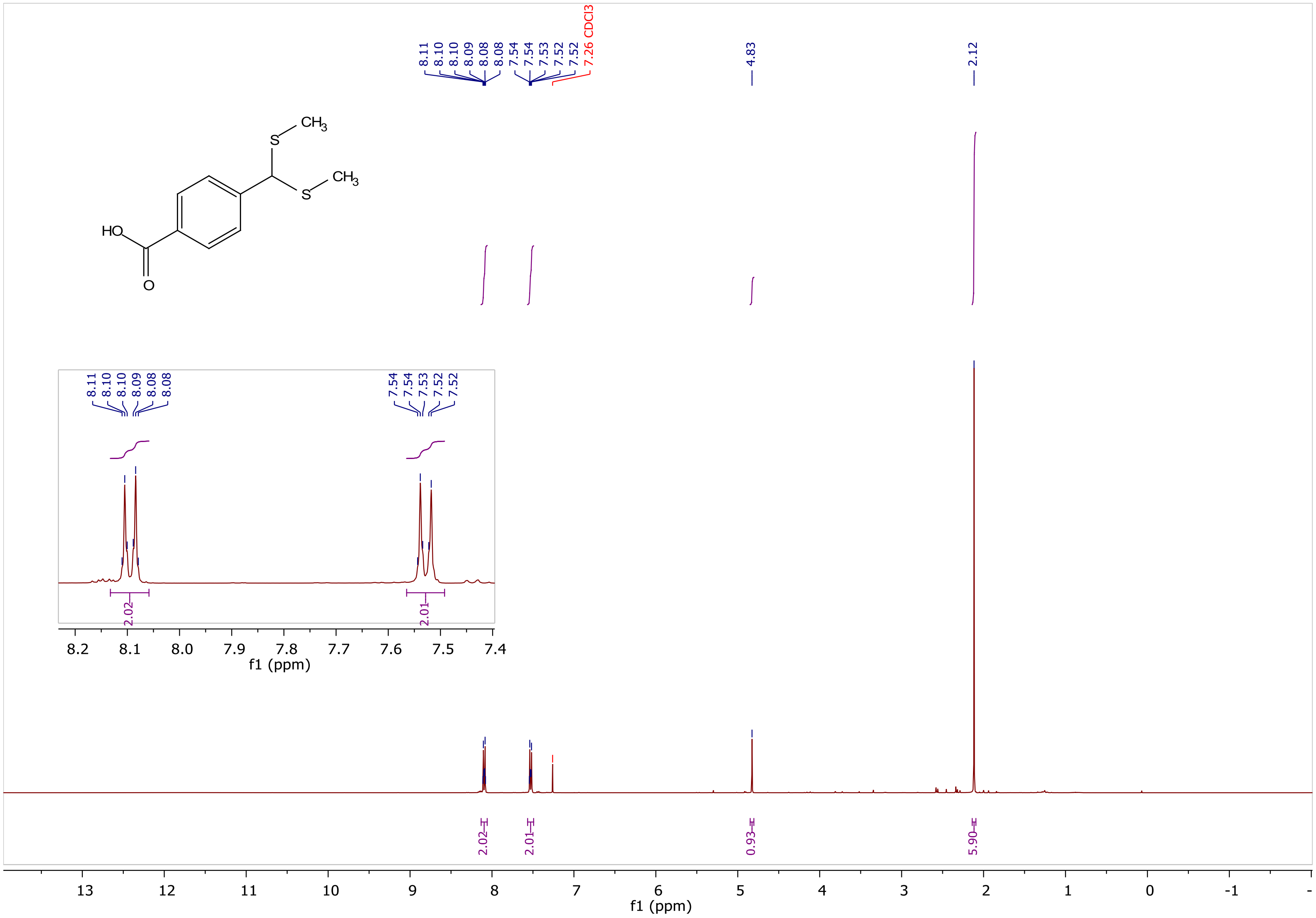

Figure S27. ^{1}H-NMR (400 MHz $CDCl_3$) of compound **14b.**

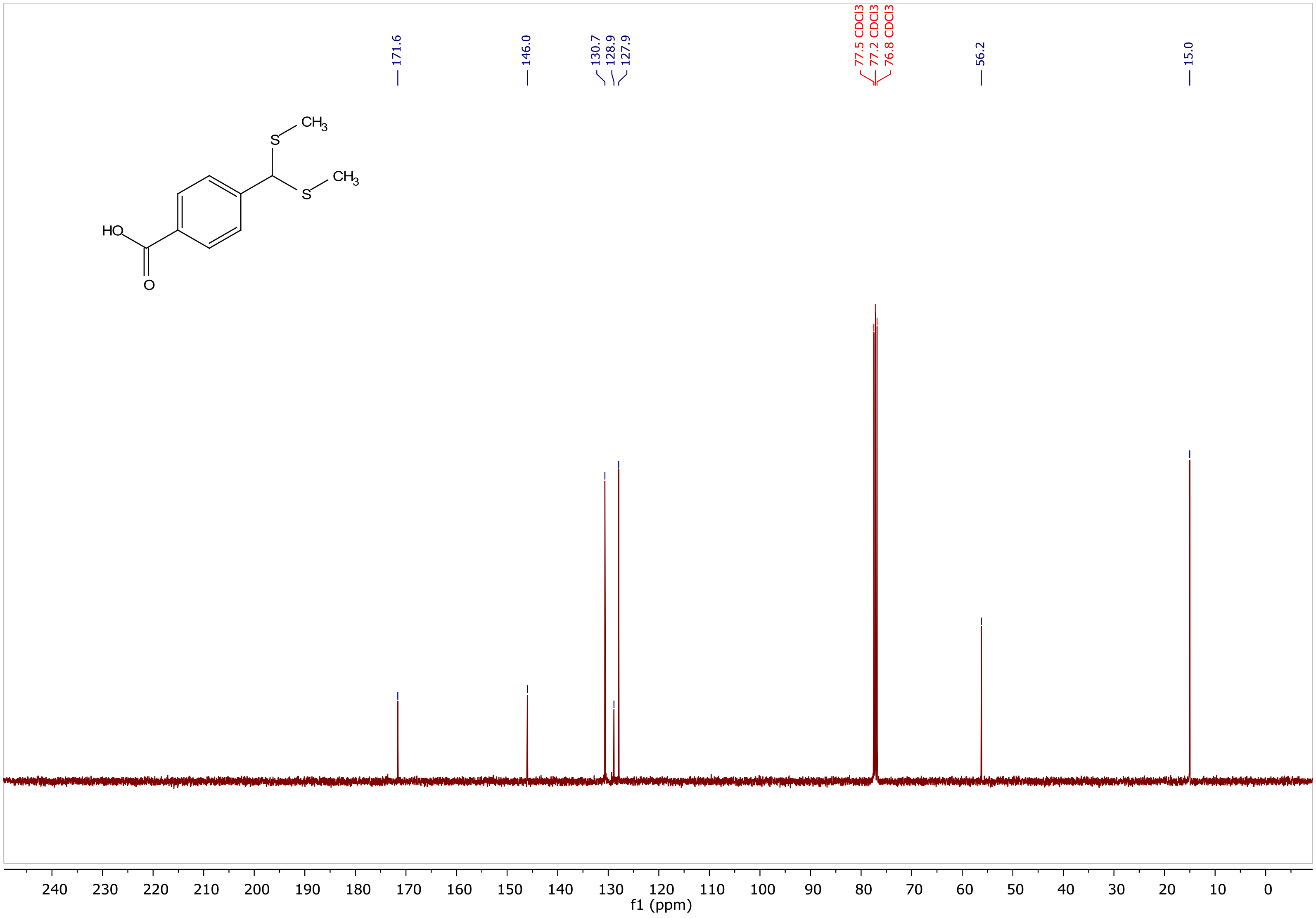

Figure S28. $^{13}C\{^{1}H\}$-NMR (101 MHz, $CDCl_3$) of compound **14b.**

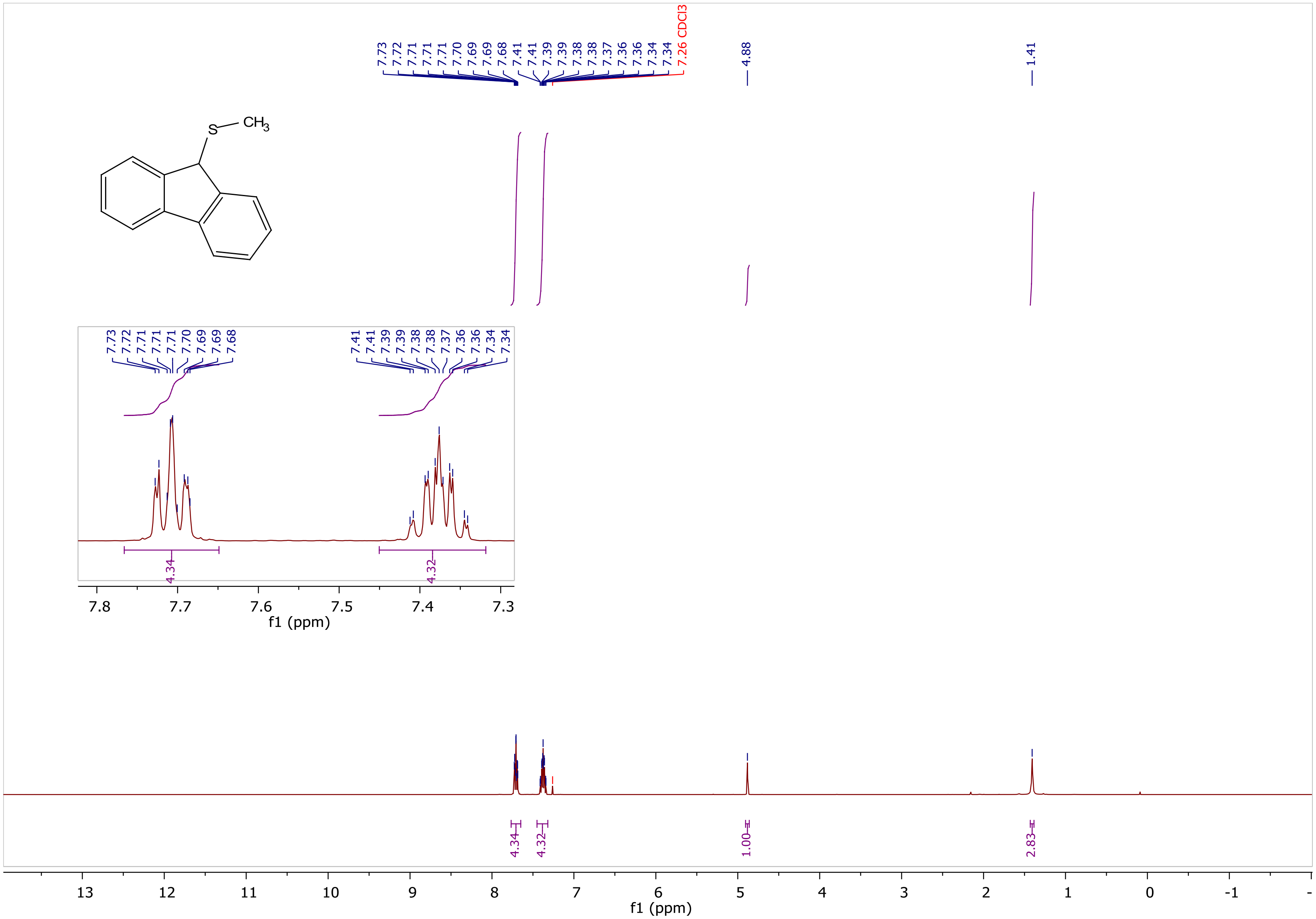

Figure S29. ^{1}H-NMR (400 MHz $CDCl_3$) of compound **15b.**

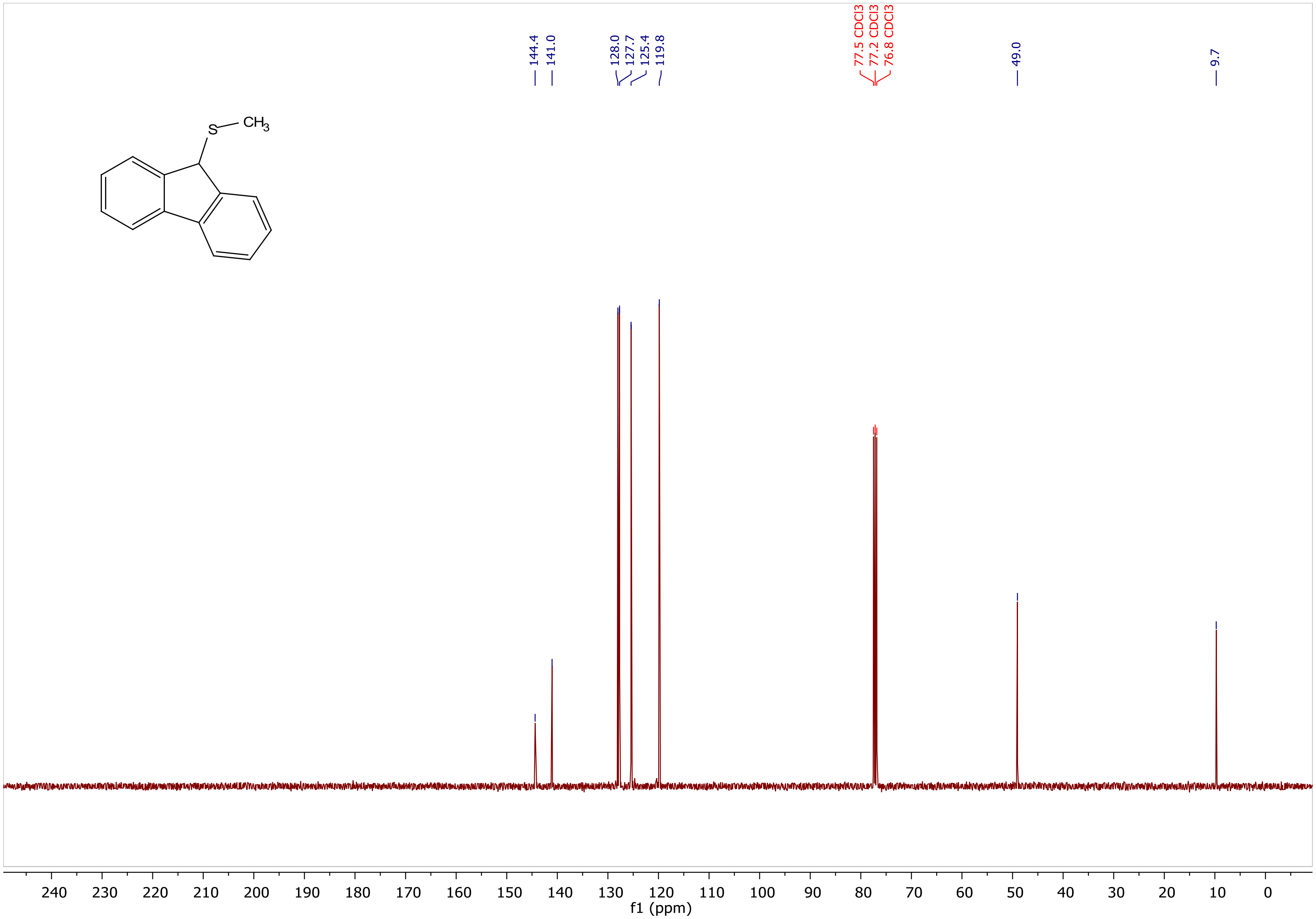

Figure S30. $^{13}C\{^{1}H\}$-NMR (101 MHz, $CDCl_3$) of compound 1**5b.**

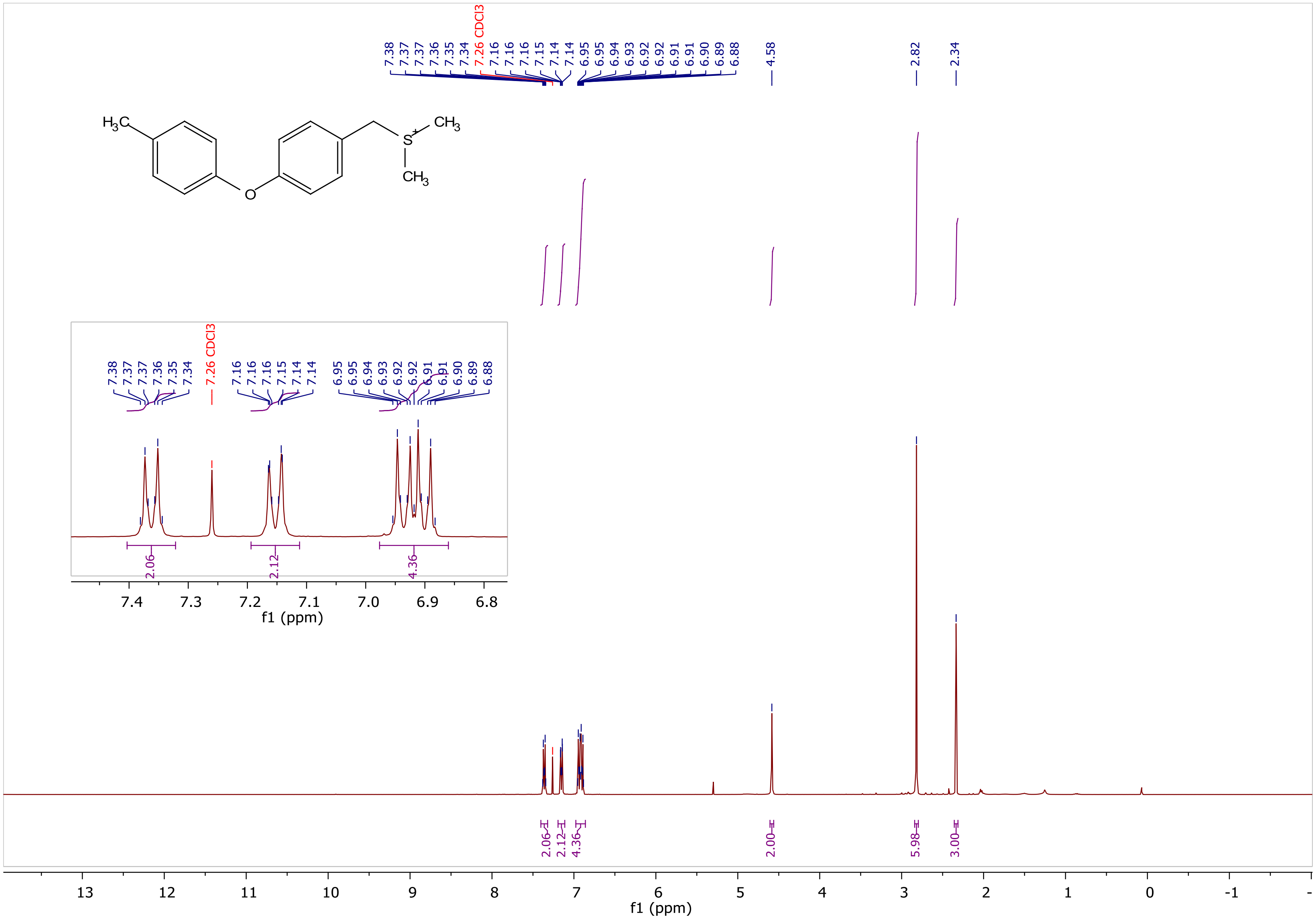

Figure S31. ^{1}H-NMR (400 MHz $CDCl_3$) of compound **12ba.**

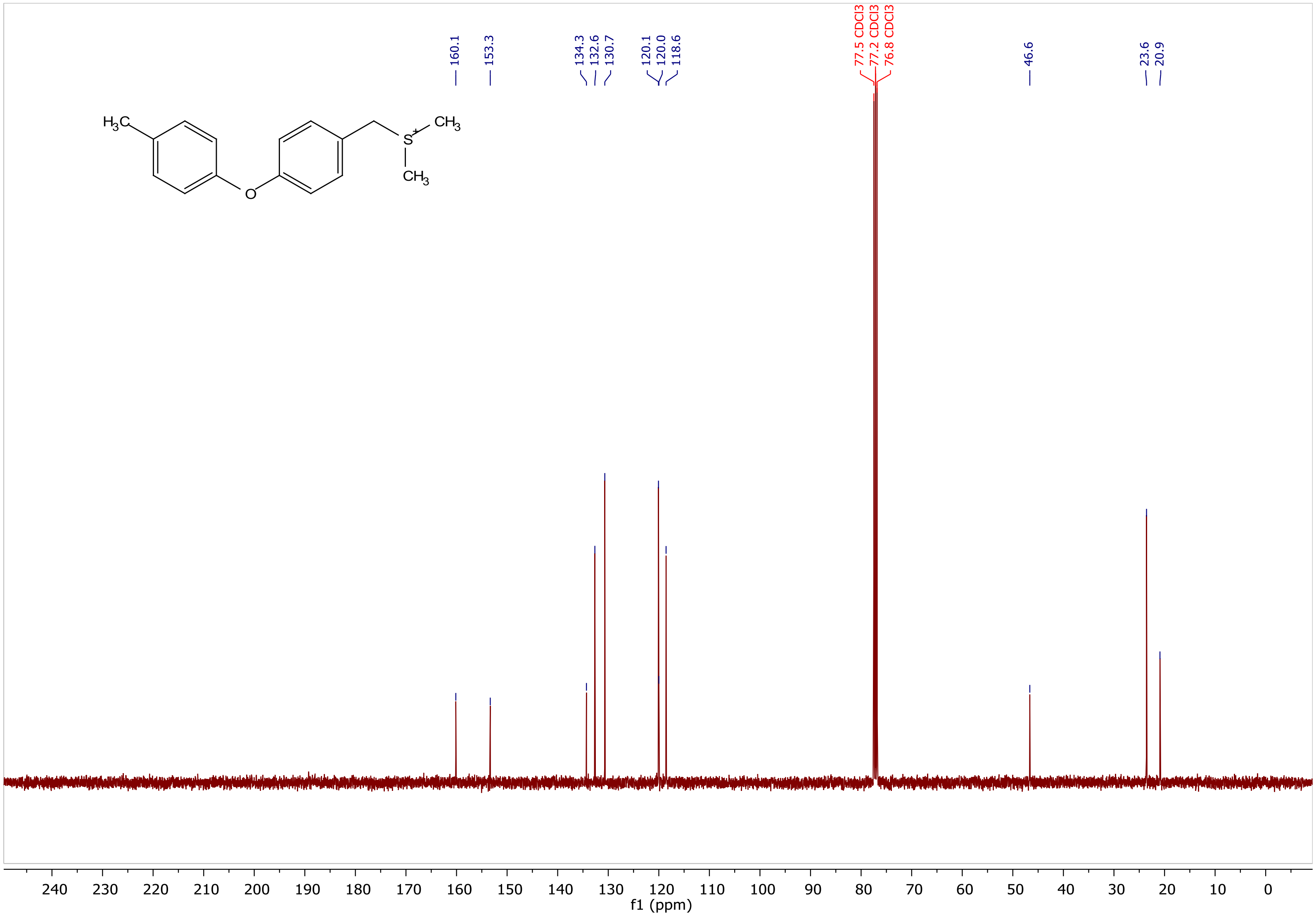

Figure S32. $^{13}C\{^{1}H\}$-NMR (101 MHz, $CDCl_3$) of compound **12ba.**

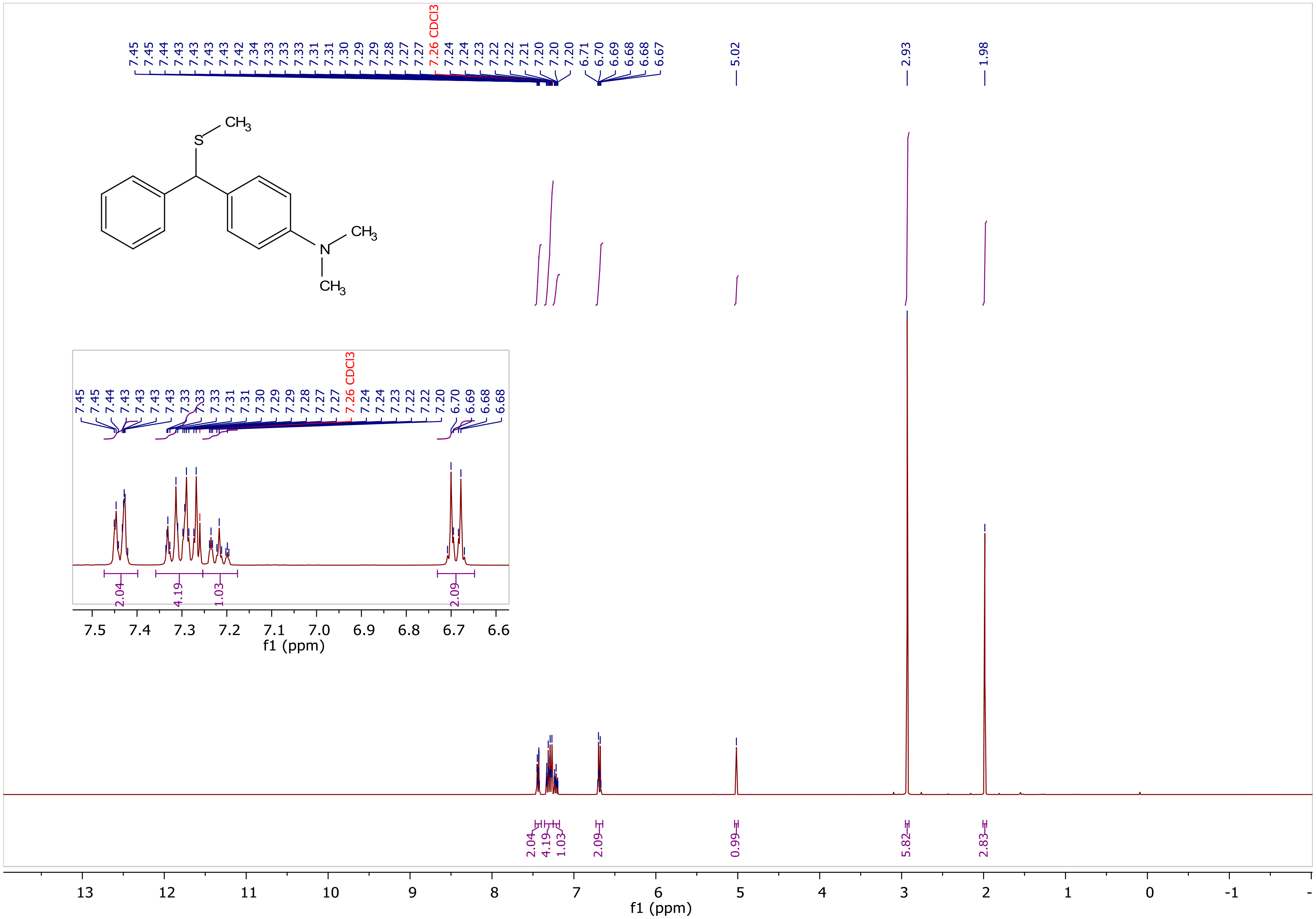

Figure S33. ^{1}H-NMR (400 MHz $CDCl_3$) of compound **1d.**

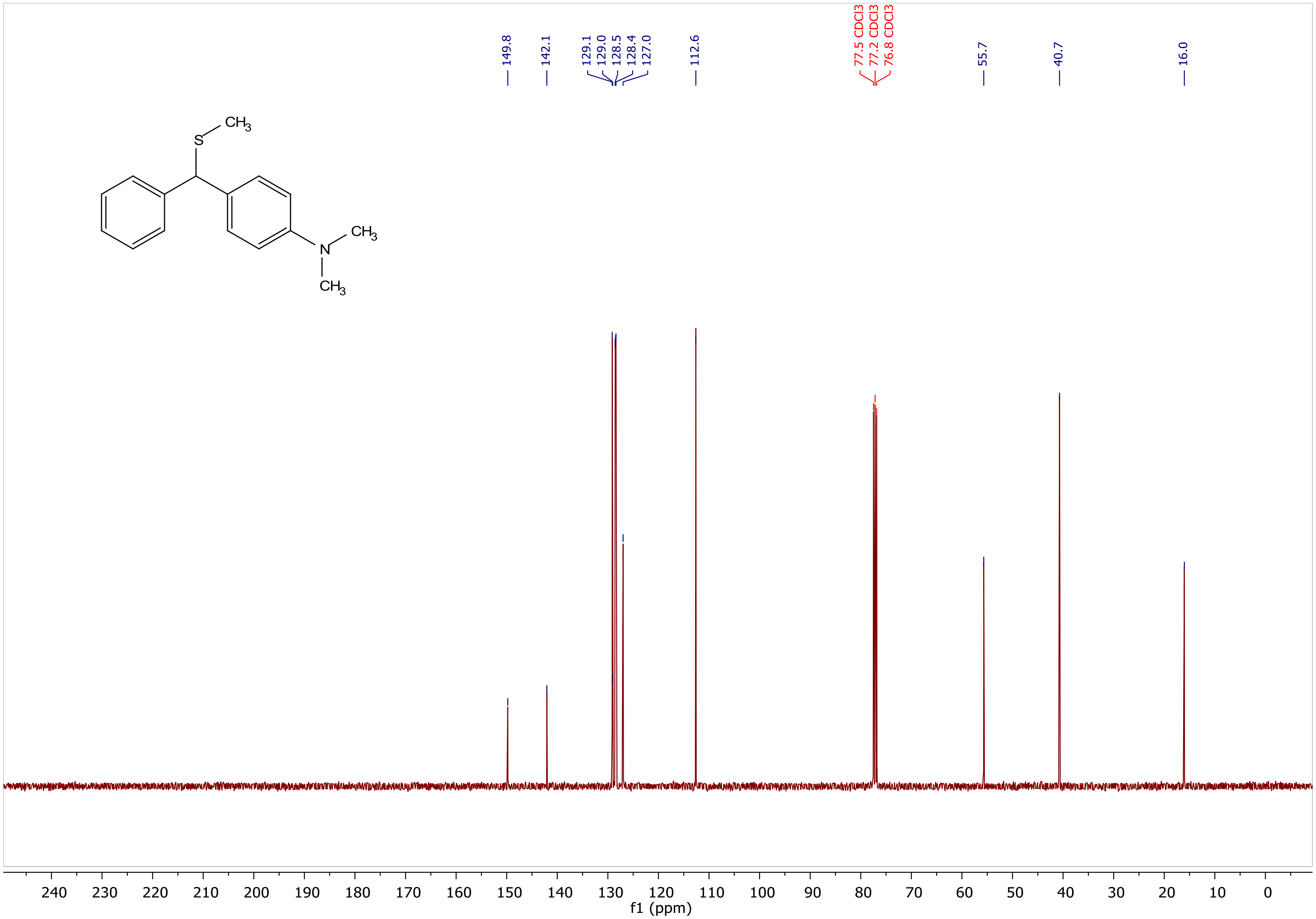

Figure S34. $^{13}C\{^{1}H\}$-NMR (101 MHz, $CDCl_3$) of compound **1d.**

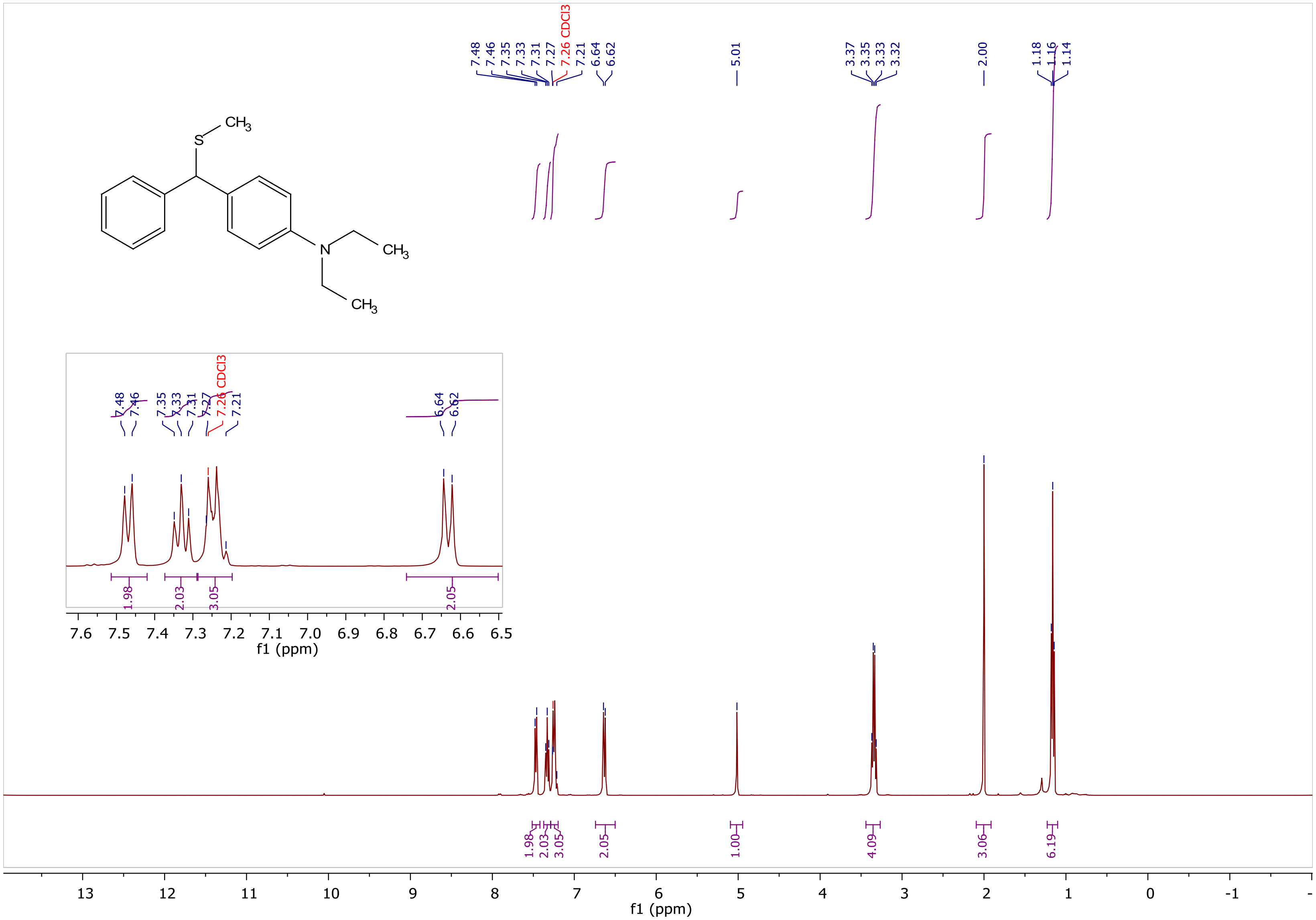

Figure S35. ^{1}H-NMR (400 MHz CDCl3) of compound **2d**.

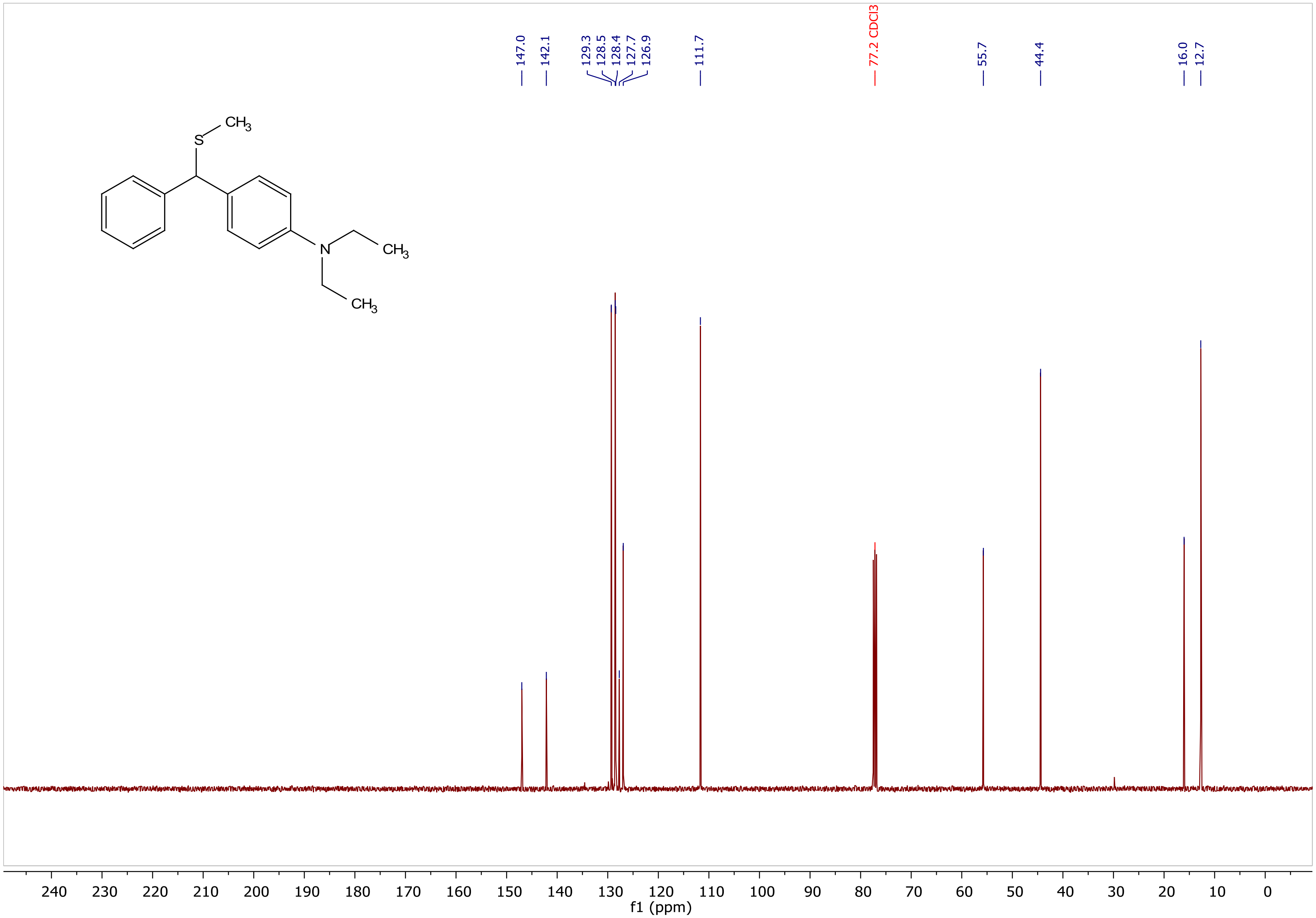

Figure S36. $^{13}C\{^{1}H\}$-NMR (101 MHz, $CDCl_3$) of compound **2d.**

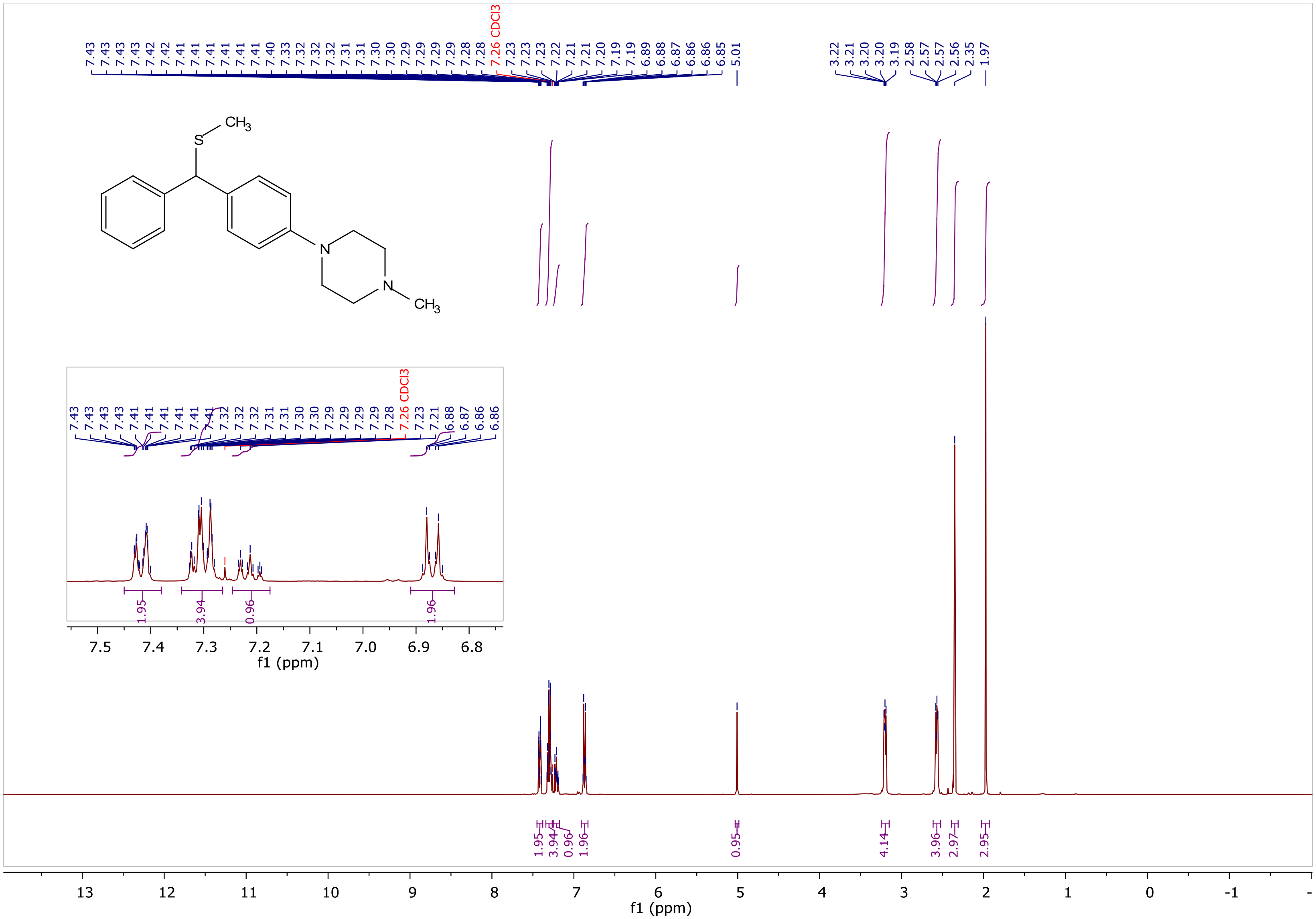

Figure S37. ^{1}H-NMR (400 MHz $CDCl_3$) of compound **3d.**

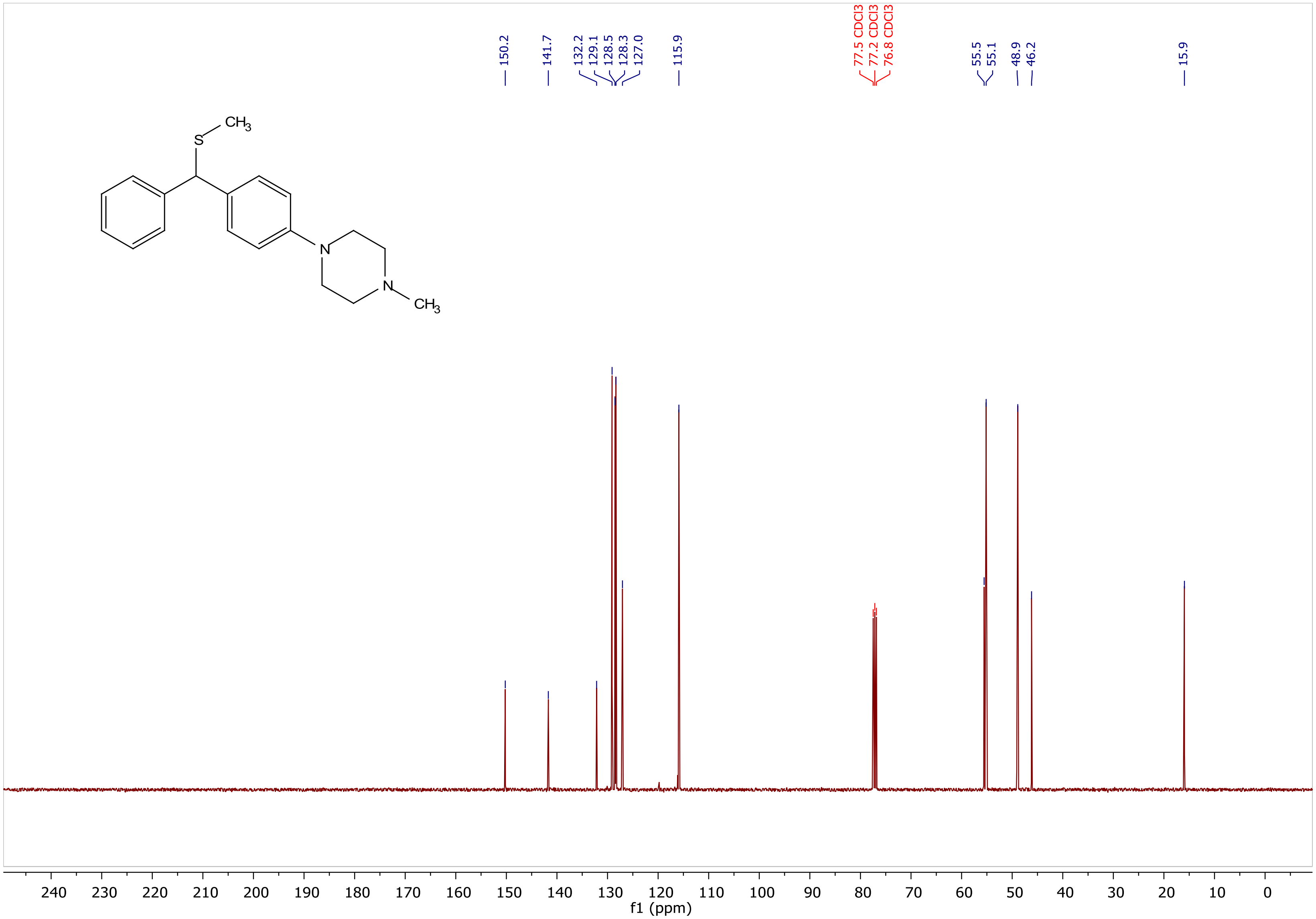

Figure S38. $^{13}C\{^{1}H\}$-NMR (101 MHz, $CDCl_3$) of compound **3d.**

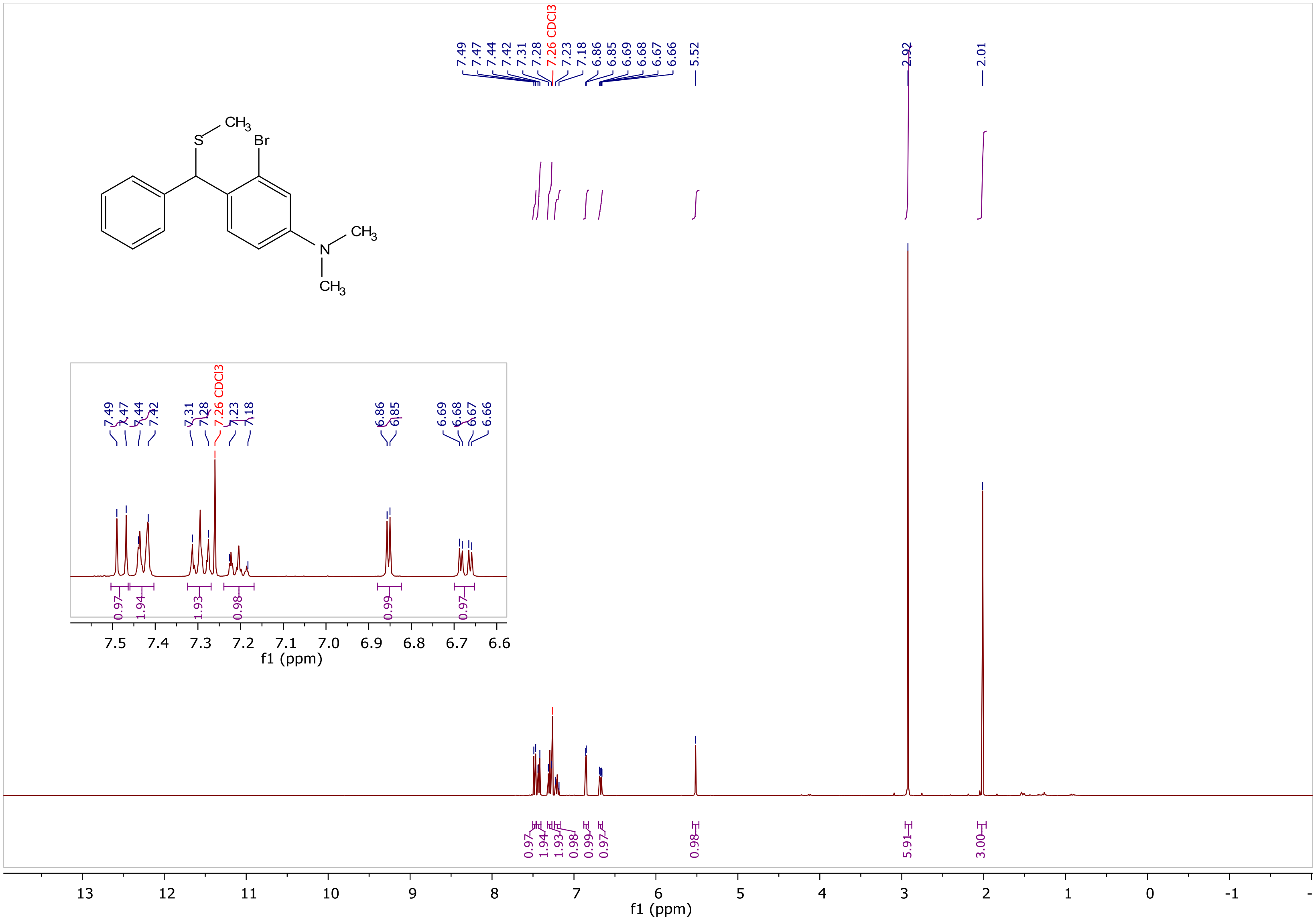

Figure S39. ^{1}H-NMR (400 MHz $CDCl_3$) of compound **4d.**

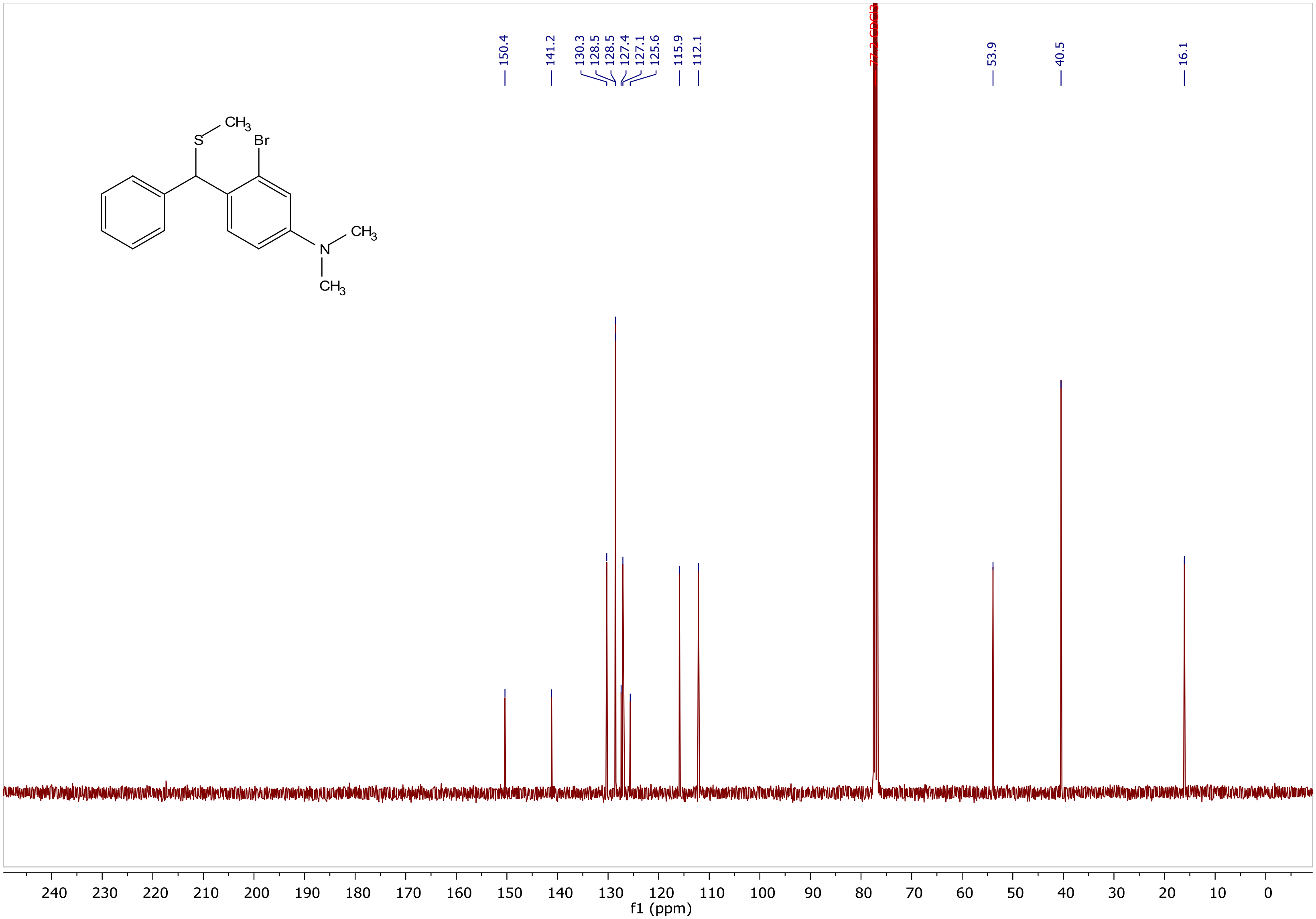

Figure S40. $^{13}C\{^{1}H\}$-NMR (101 MHz, $CDCl_3$) of compound **4d.**

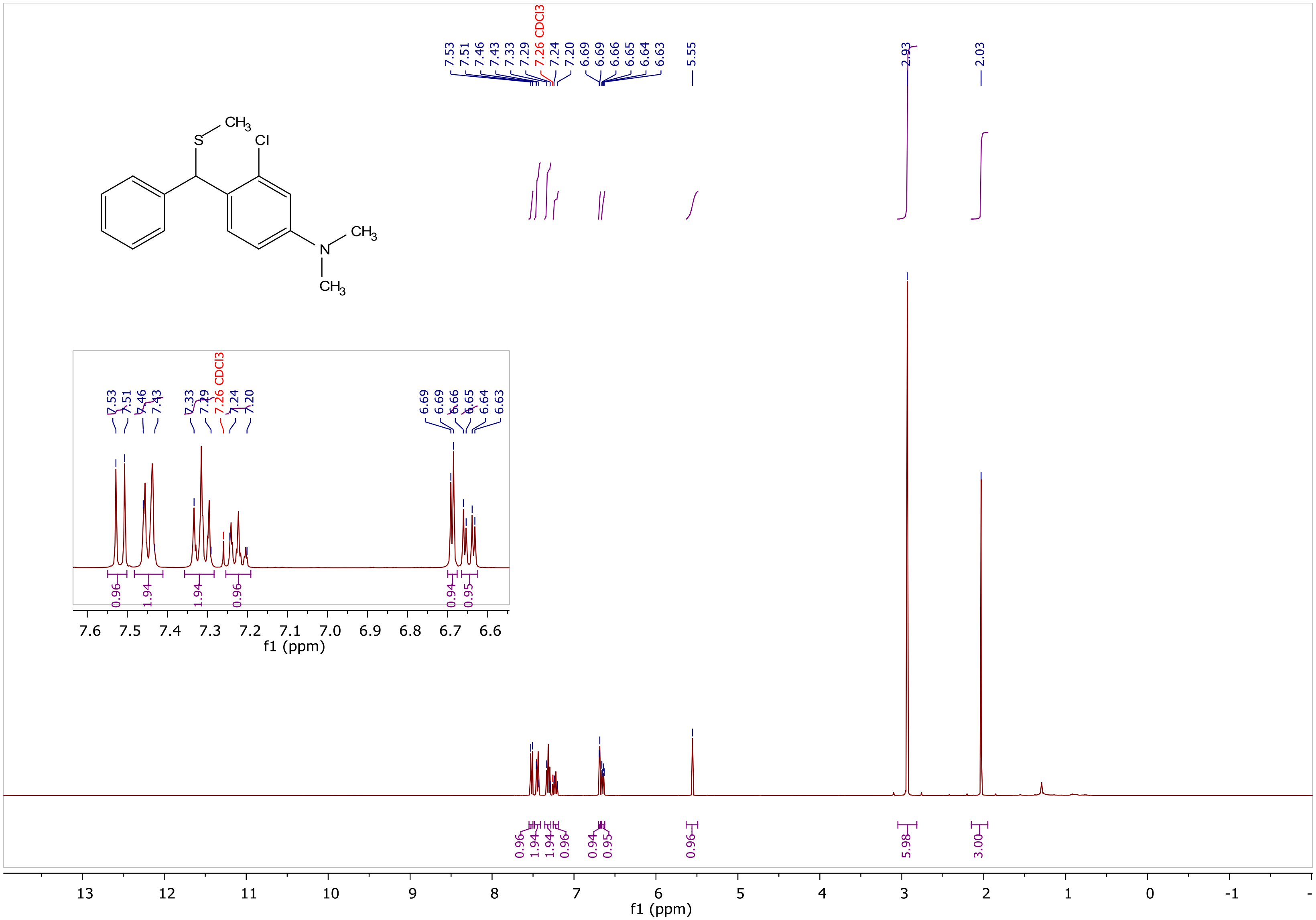

Figure S41. ^{1}H-NMR (400 MHz $CDCl_3$) of compound **5d.**

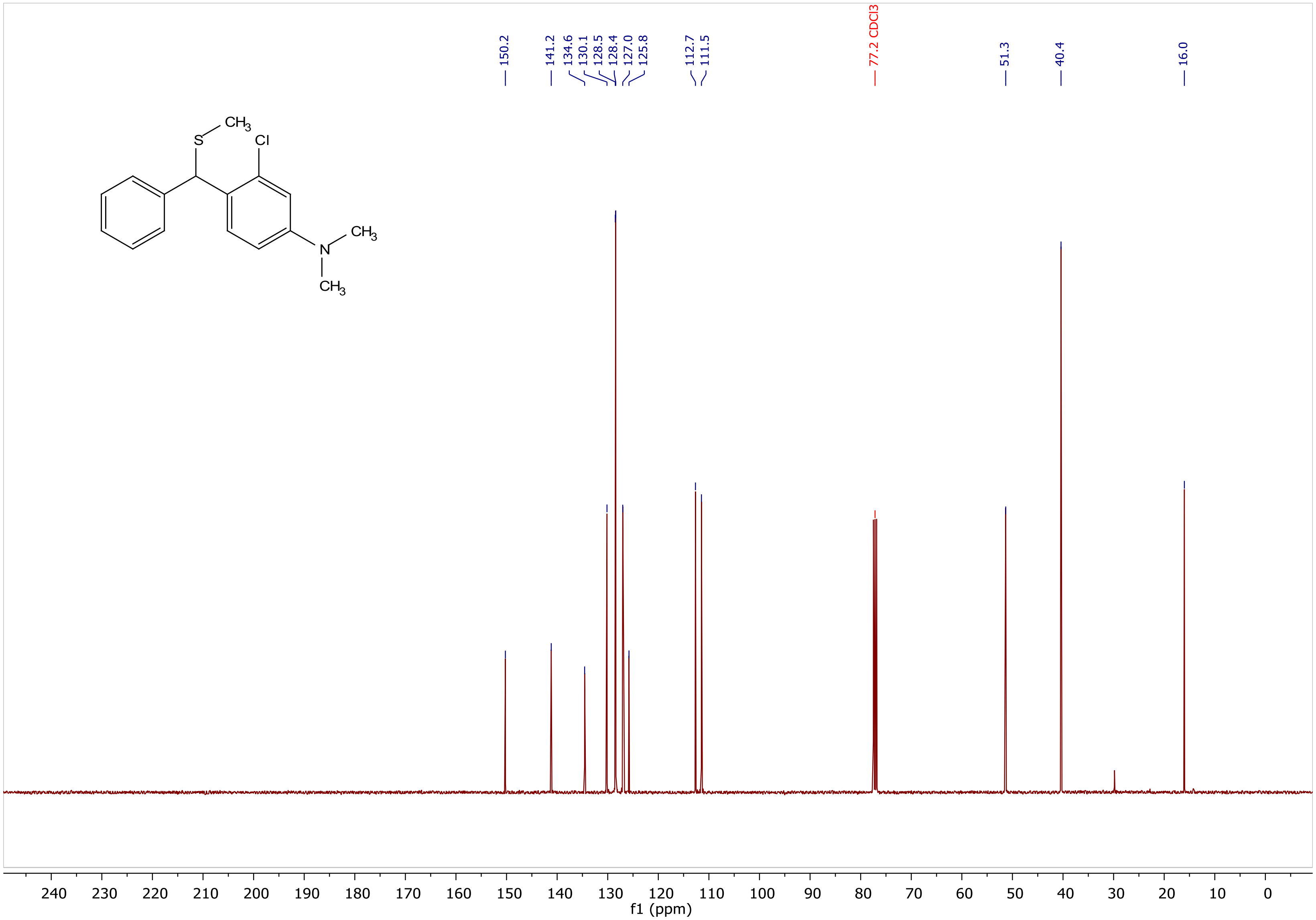

Figure S42. $^{13}C\{^{1}H\}$-NMR (101 MHz, $CDCl_3$) of compound **5d.**

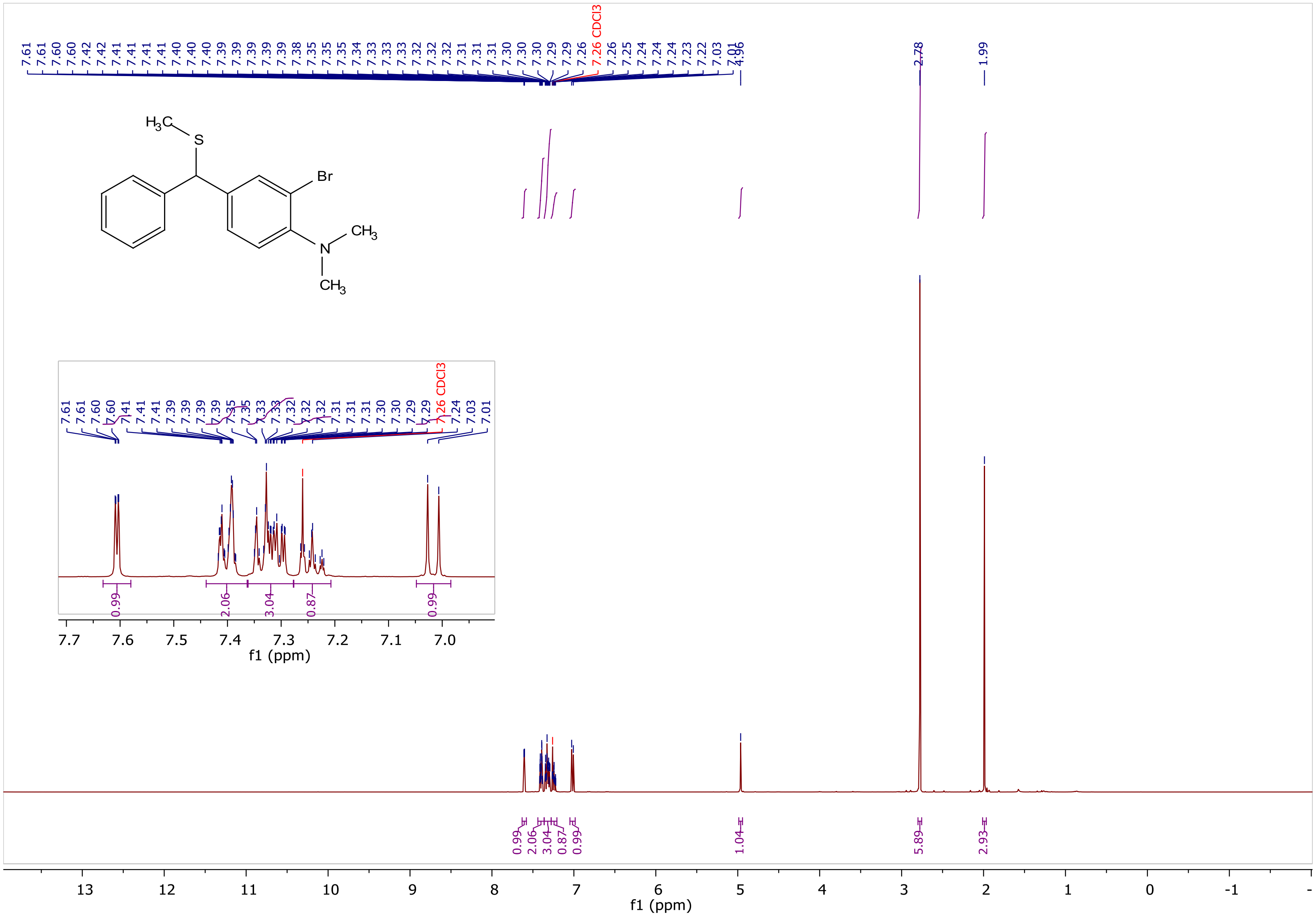

Figure S43. ^{1}H-NMR (400 MHz $CDCl_3$) of compound **6d.**

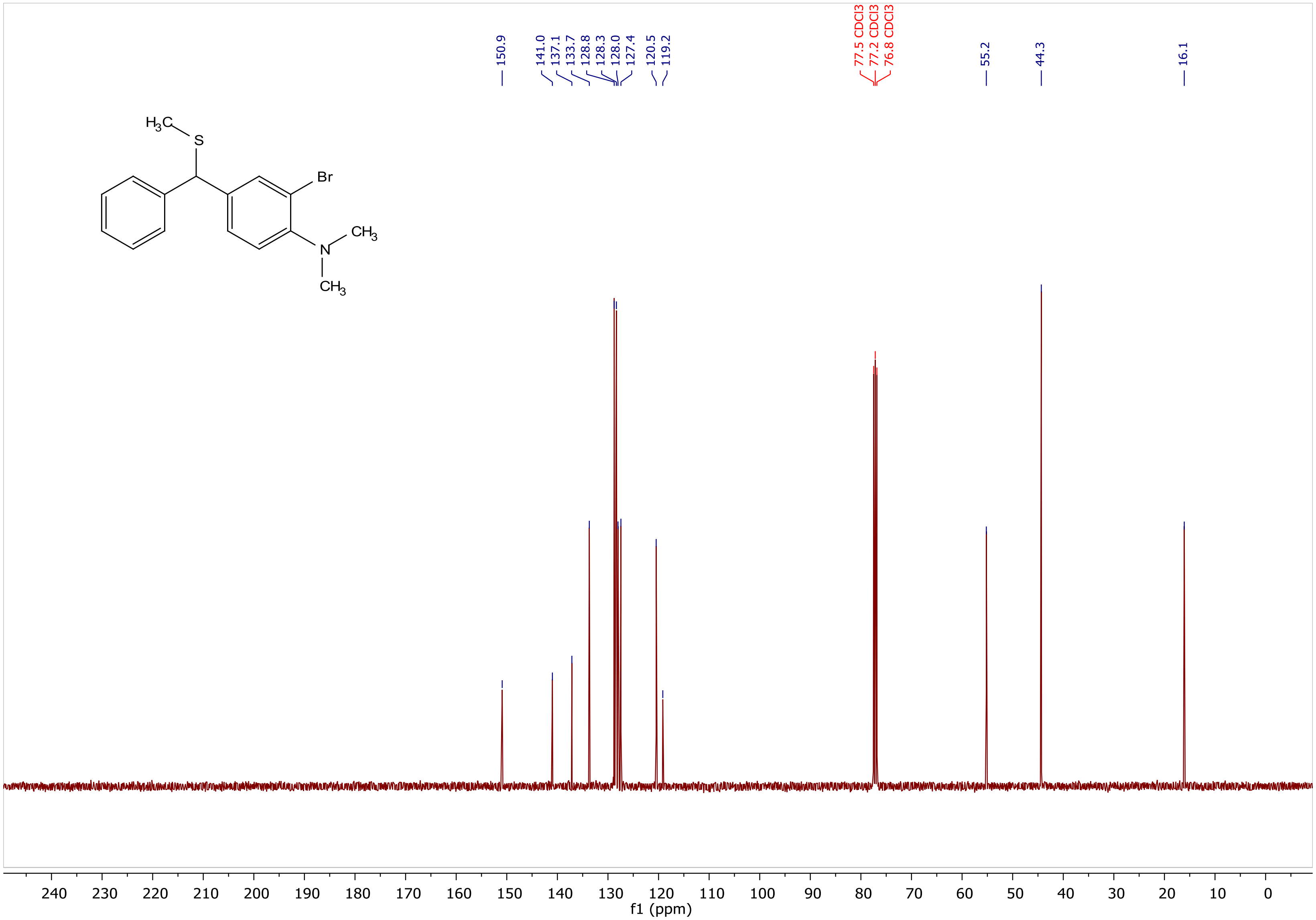

Figure S44. $^{13}C\{^{1}H\}$-NMR (101 MHz, $CDCl_3$) of compound **6d.**

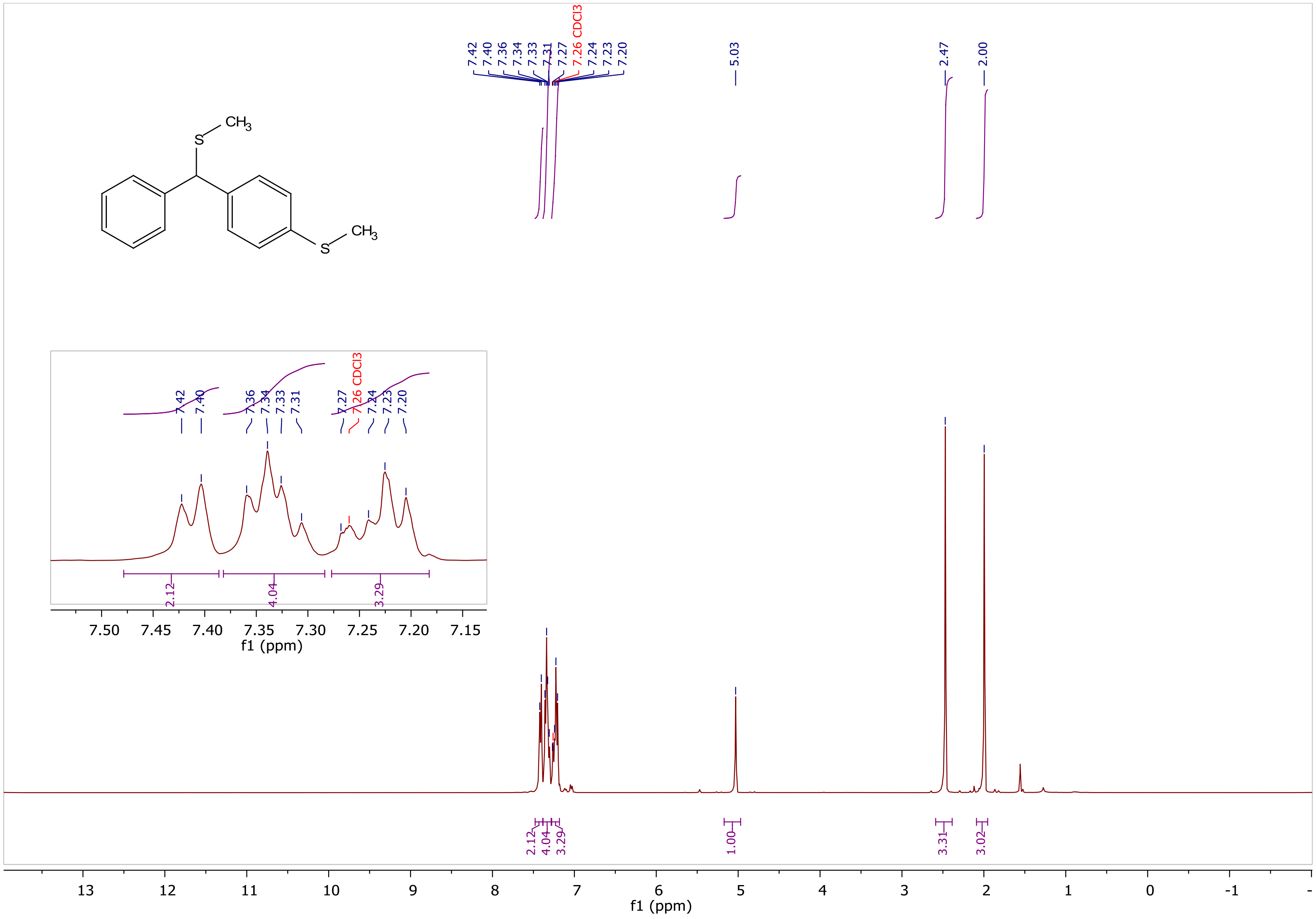

Figure S45. ^{1}H-NMR (400 MHz $CDCl_3$) of compound **7d.**

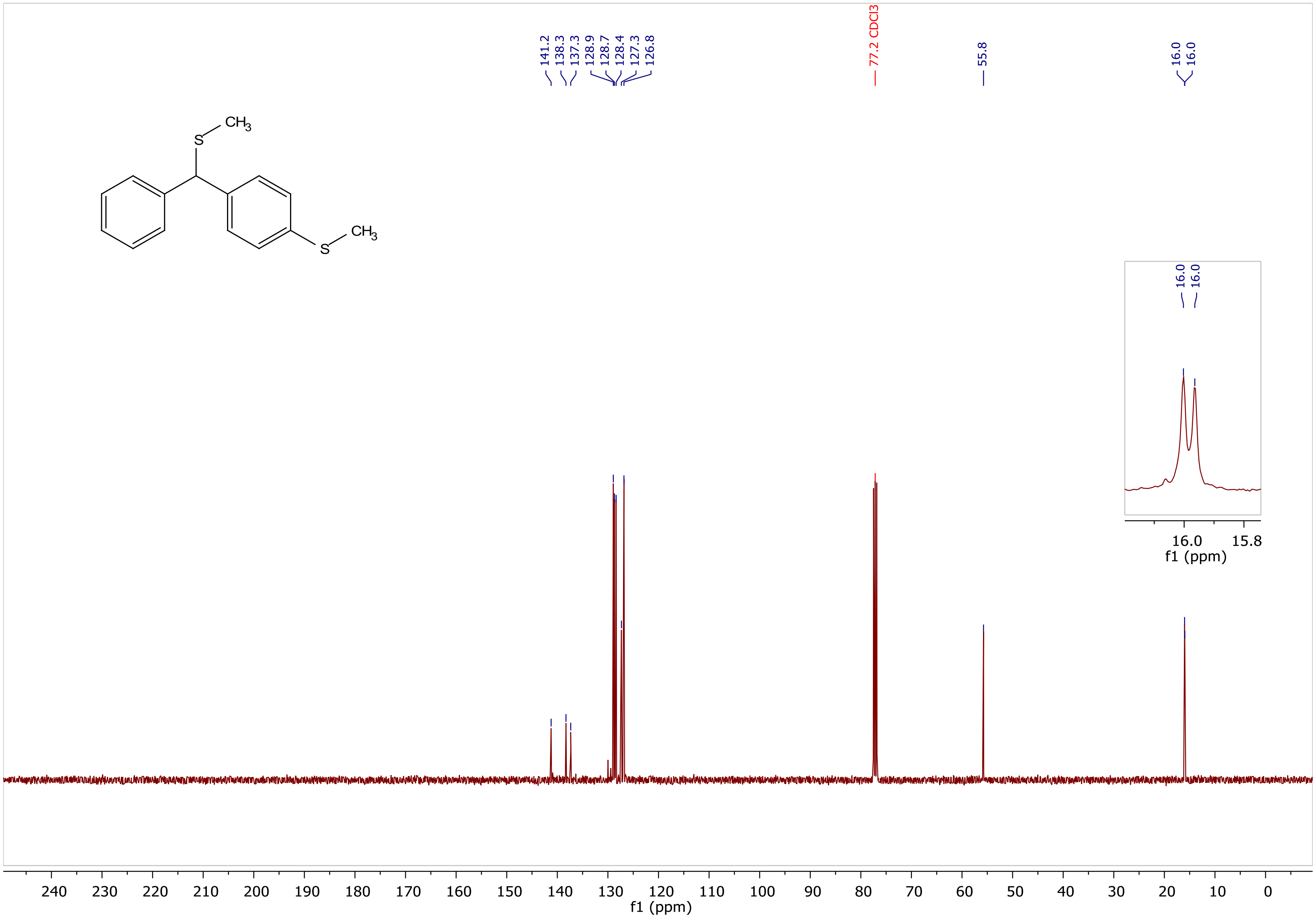

Figure S46. $^{13}C\{^{1}H\}$-NMR (101 MHz, $CDCl_3$) of compound **7d.**

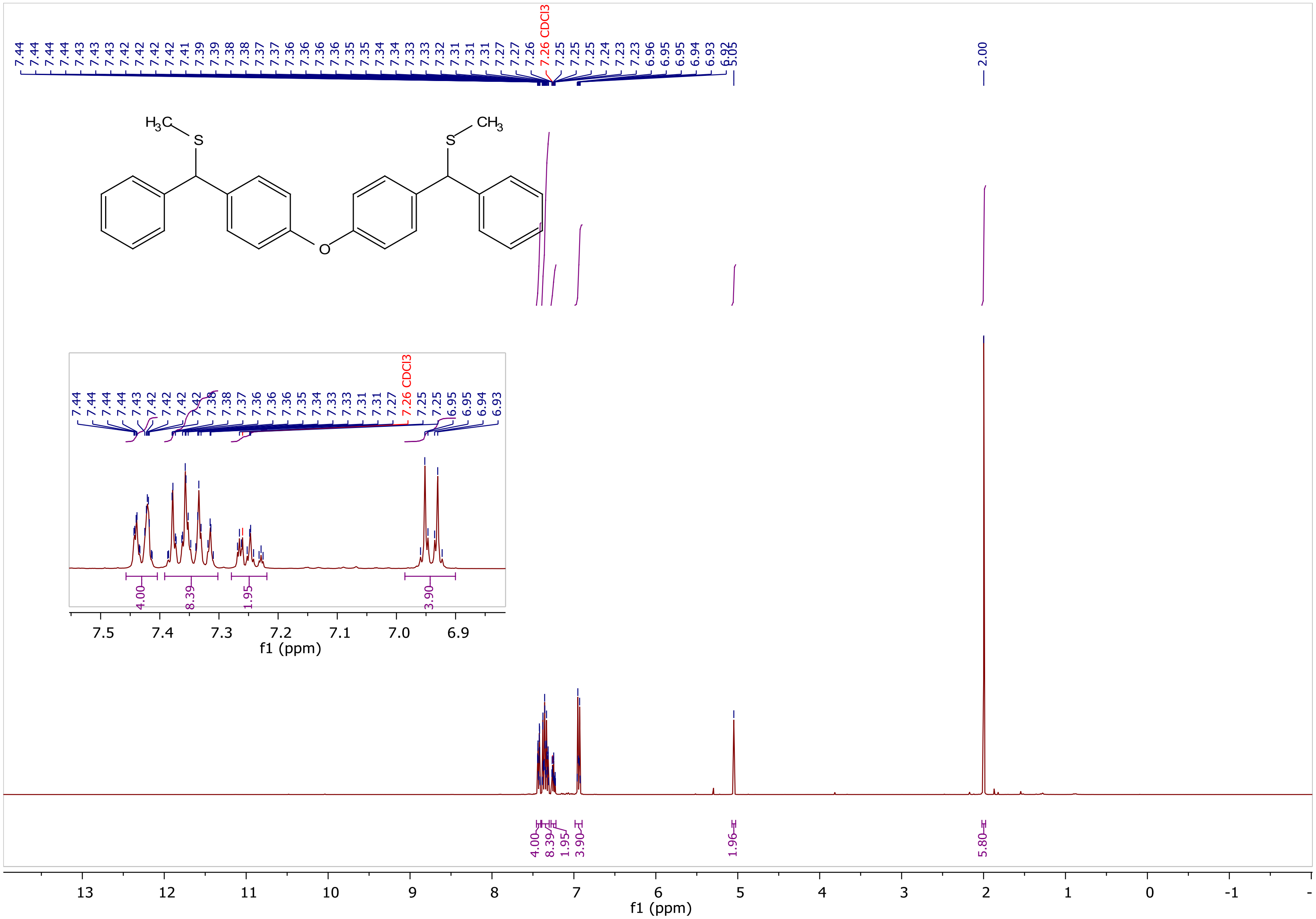

Figure S47. ^{1}H-NMR (400 MHz $CDCl_3$) of compound **8d.**

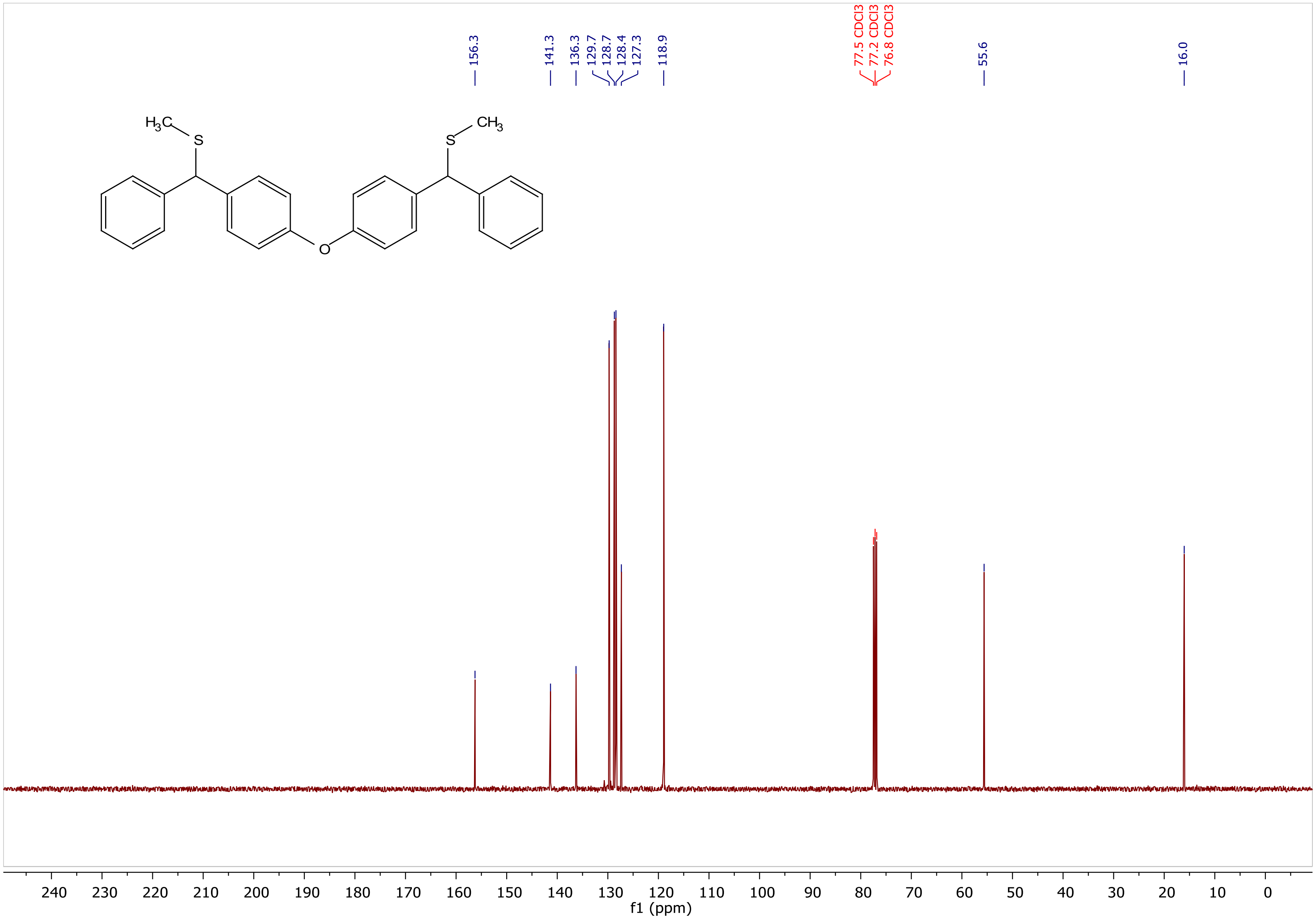

Figure S48. $^{13}C\{^{1}H\}$-NMR (101 MHz, $CDCl_3$) of compound **8d.**

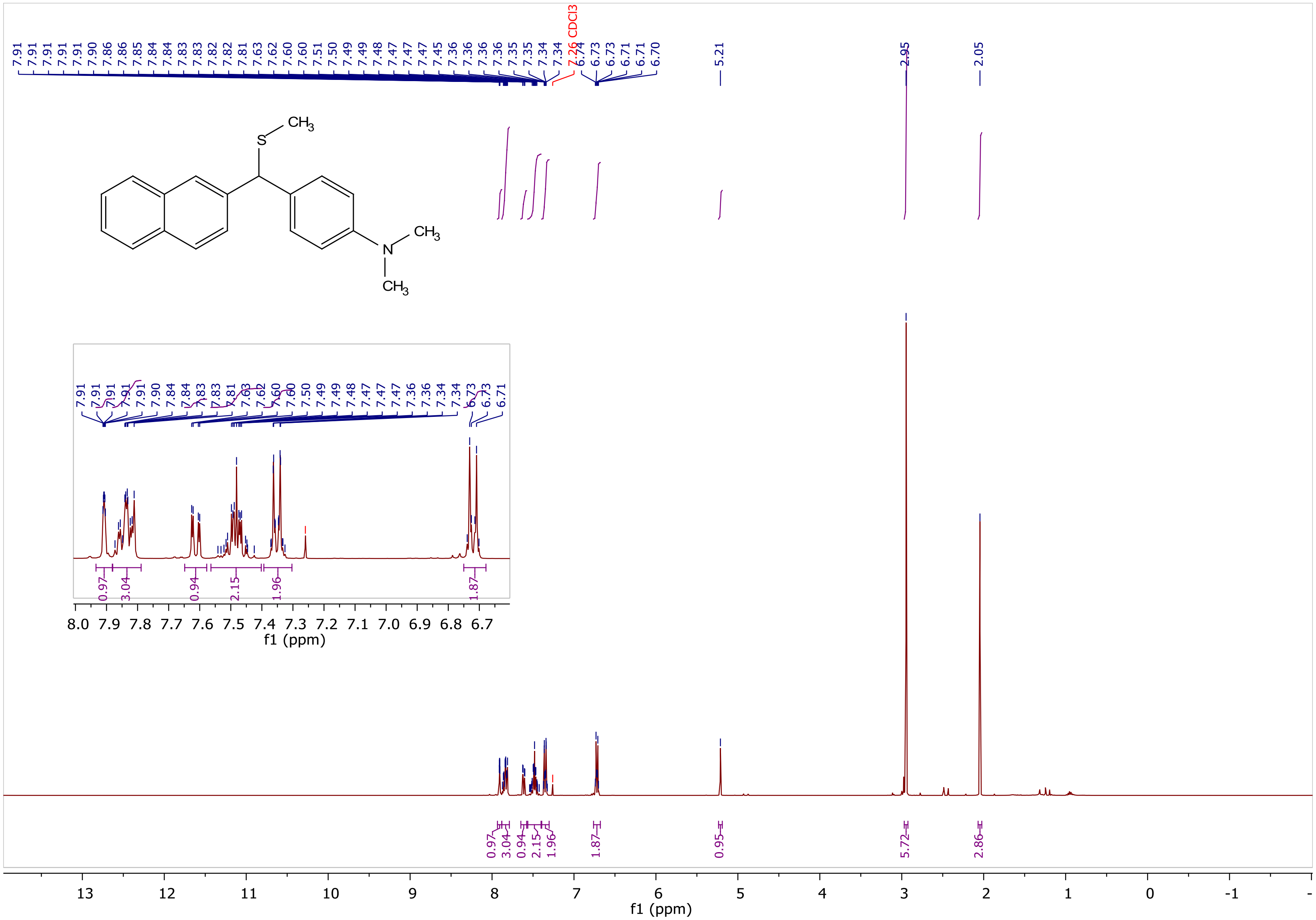

Figure S49. ^{1}H-NMR (400 MHz $CDCl_3$) of compound **9d.**

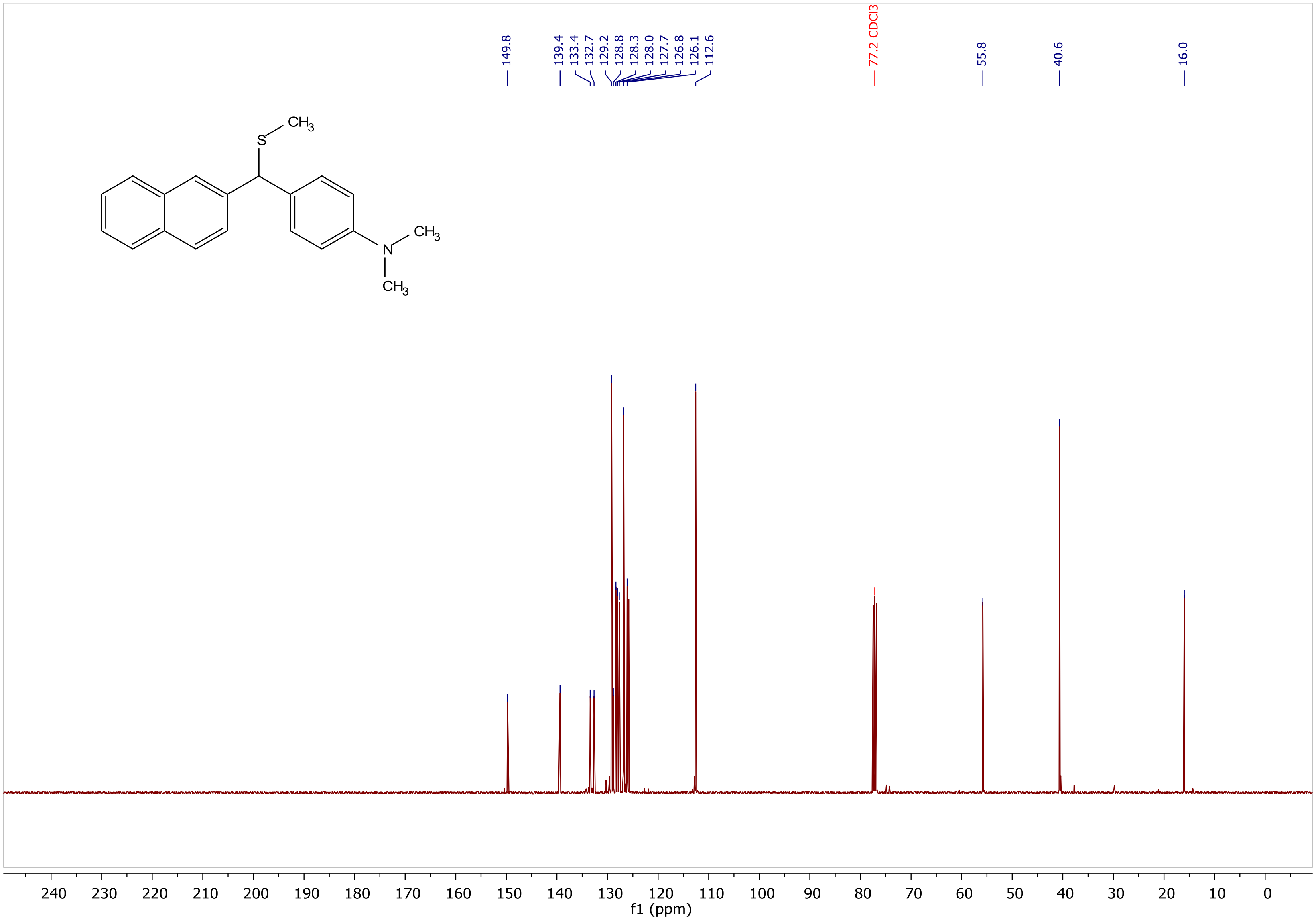

Figure S50. $^{13}C\{^{1}H\}$-NMR (101 MHz, $CDCl_3$) of compound **9d**.

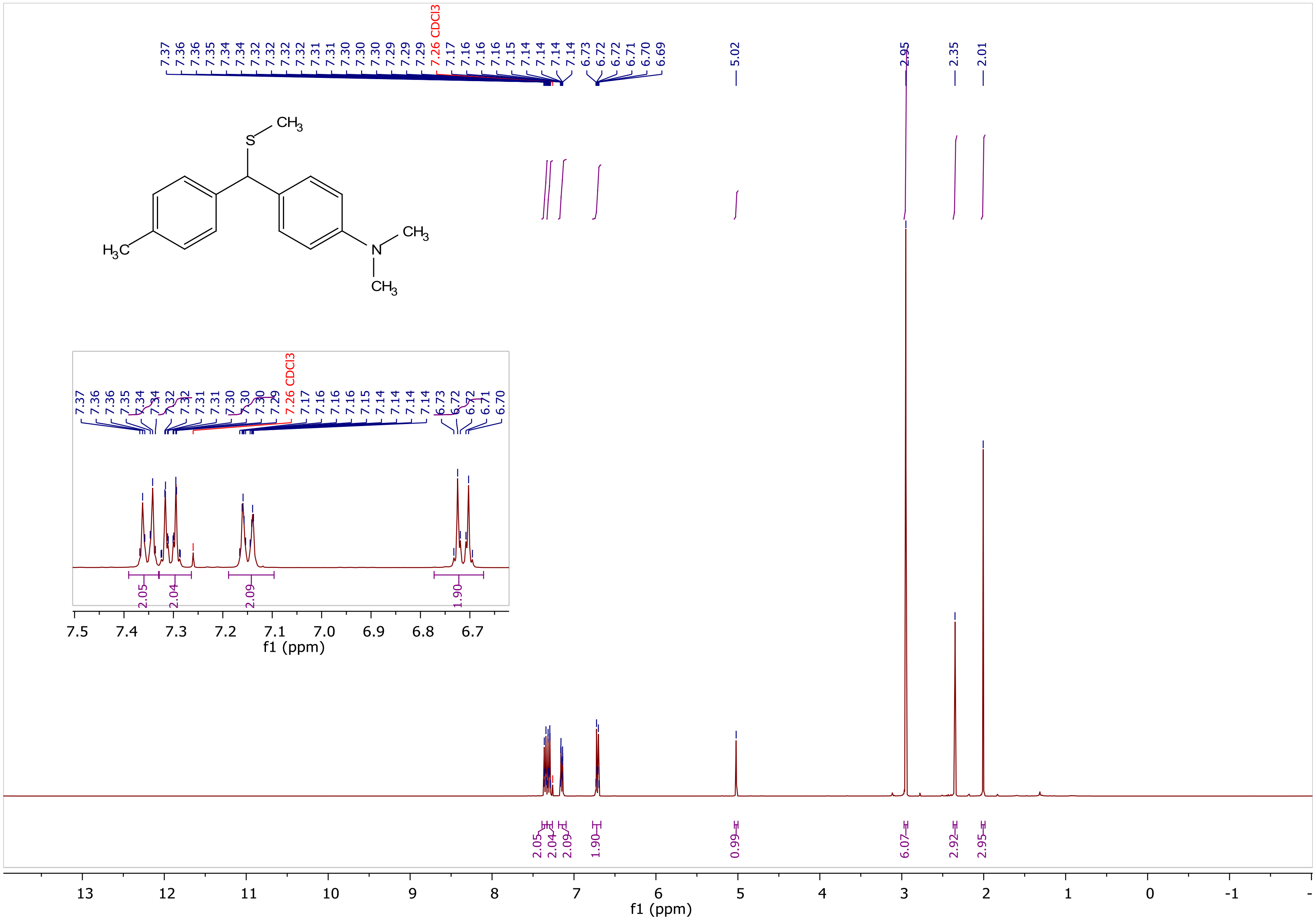

Figure S51. ^{1}H-NMR (400 MHz $CDCl_3$) of compound **10d.**

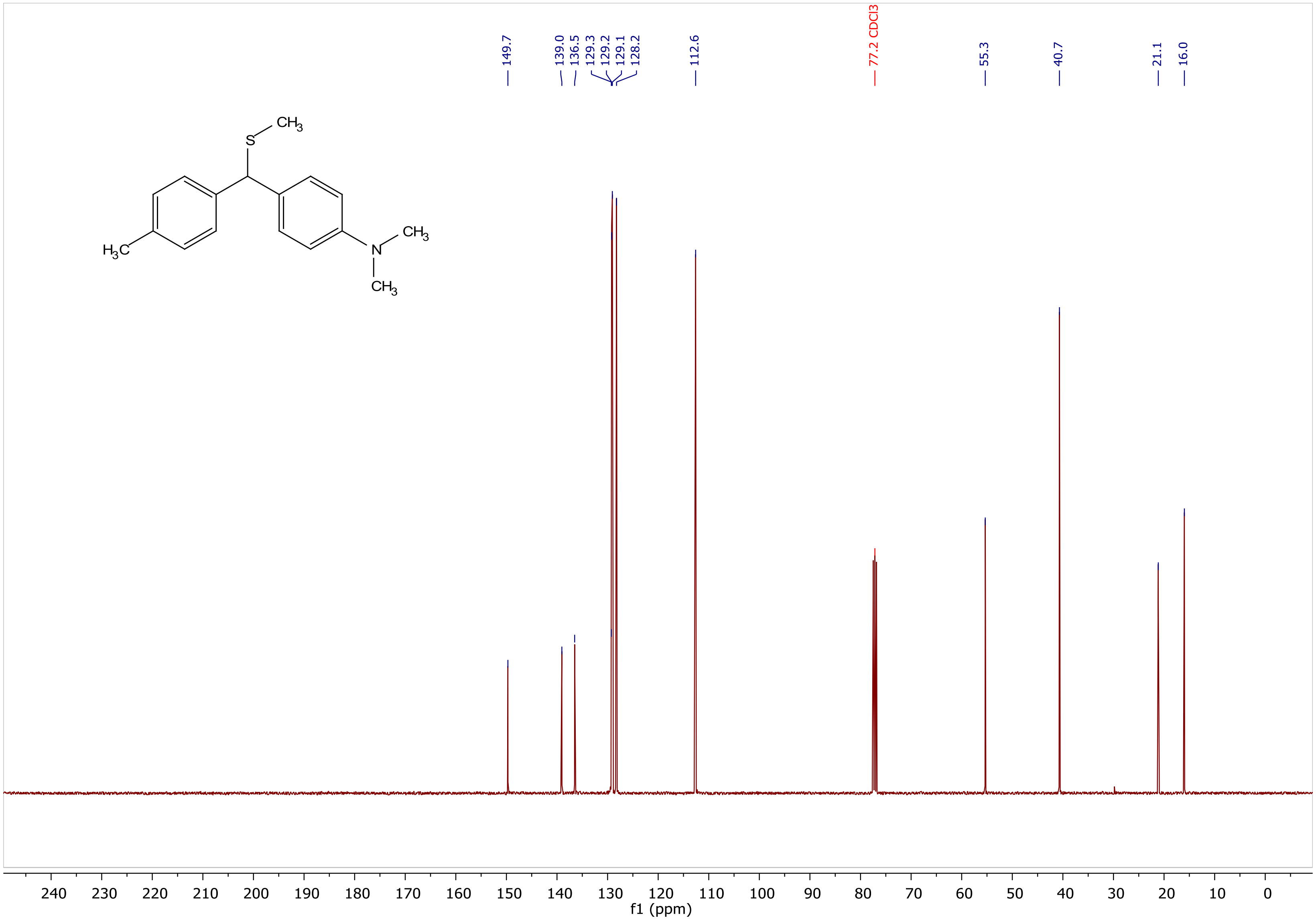

Figure S52. $^{13}C\{^{1}H\}$-NMR (101 MHz, $CDCl_3$) of compound **10d.**

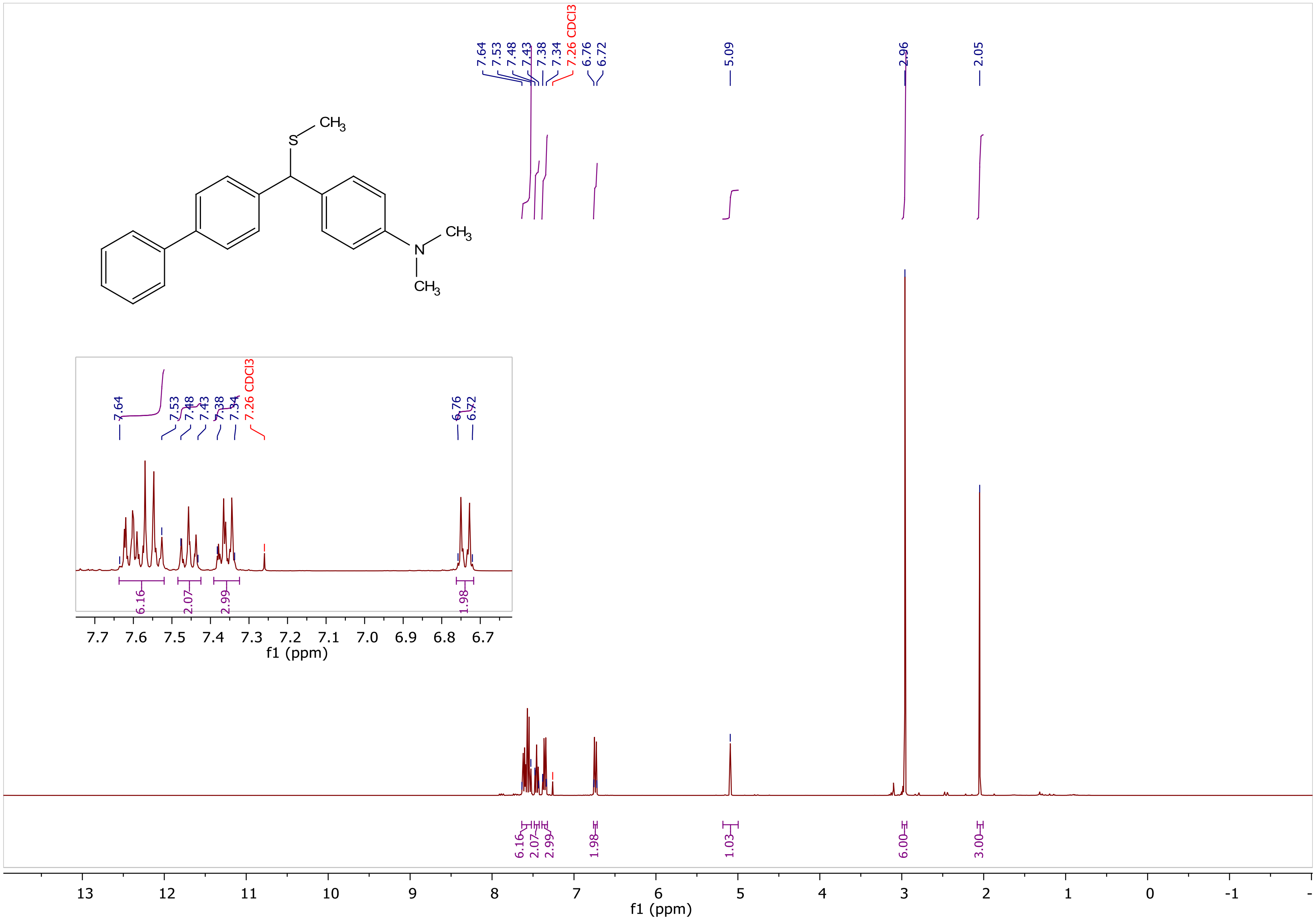

Figure S53. ^{1}H-NMR (400 MHz $CDCl_3$) of compound **11d.**

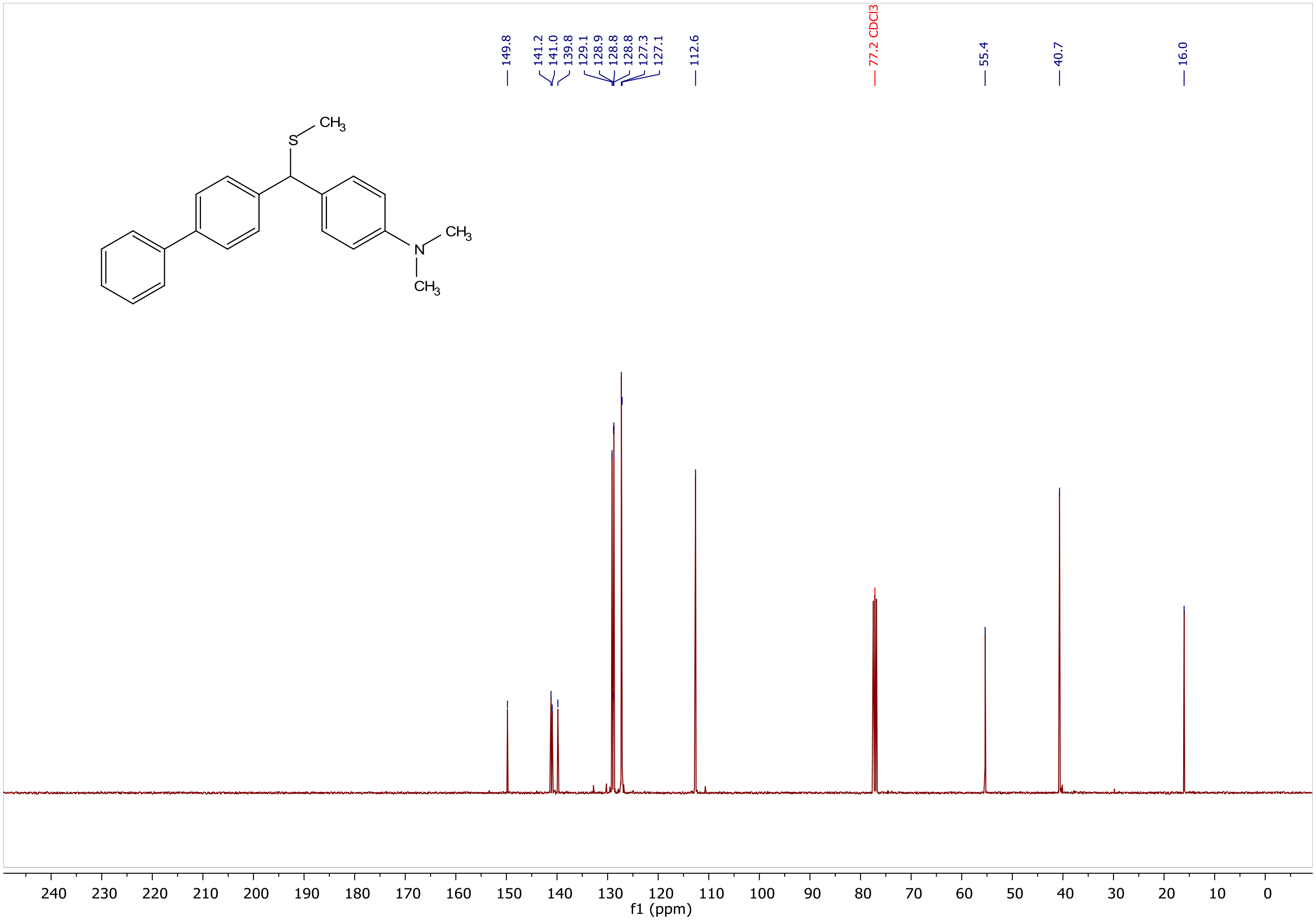

Figure S54. $^{13}C\{^{1}H\}$-NMR (101 MHz, $CDCl_3$) of compound **11d.**

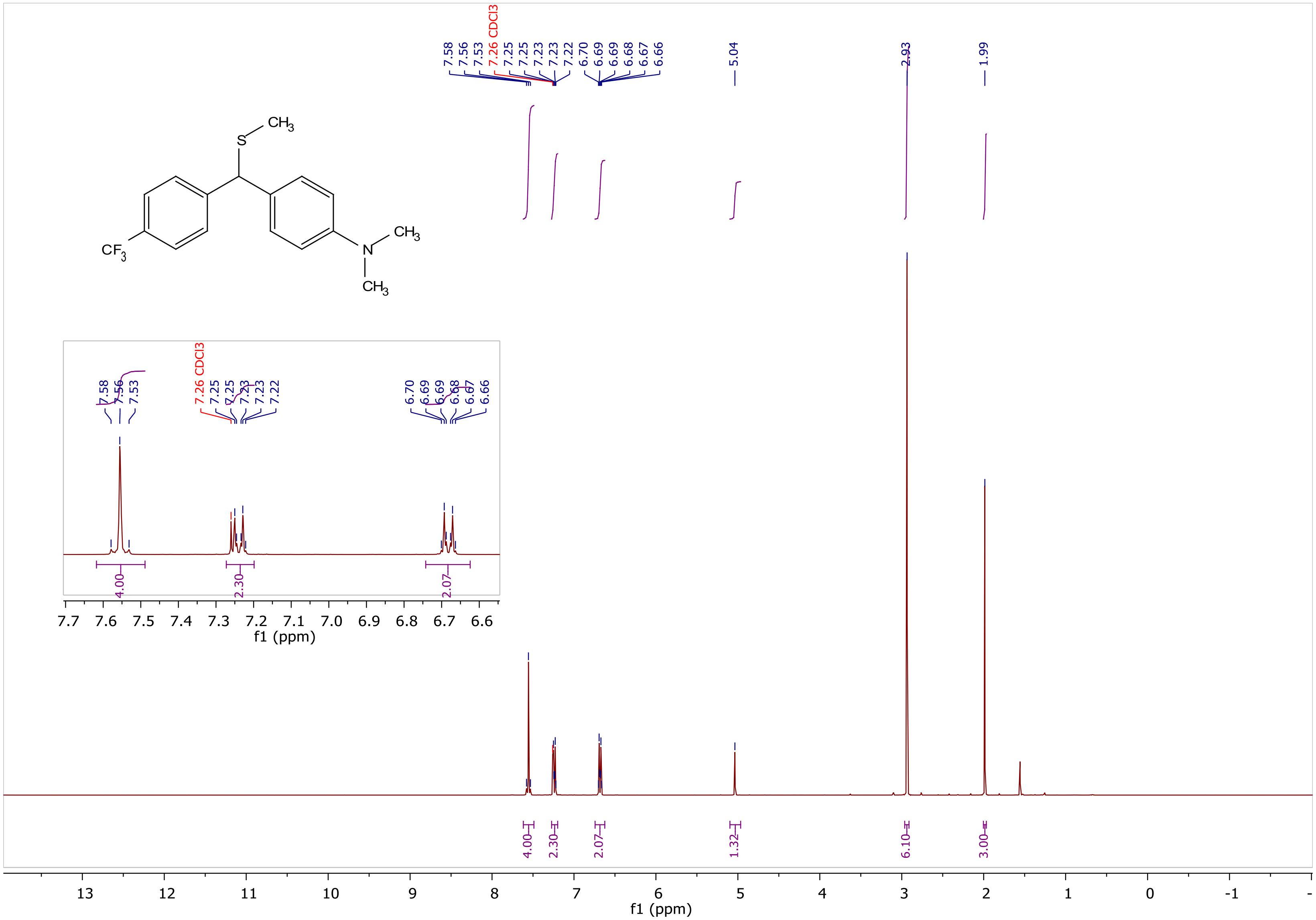

Figure S55. ^{1}H-NMR (400 MHz $CDCl_3$) of compound **12d.**

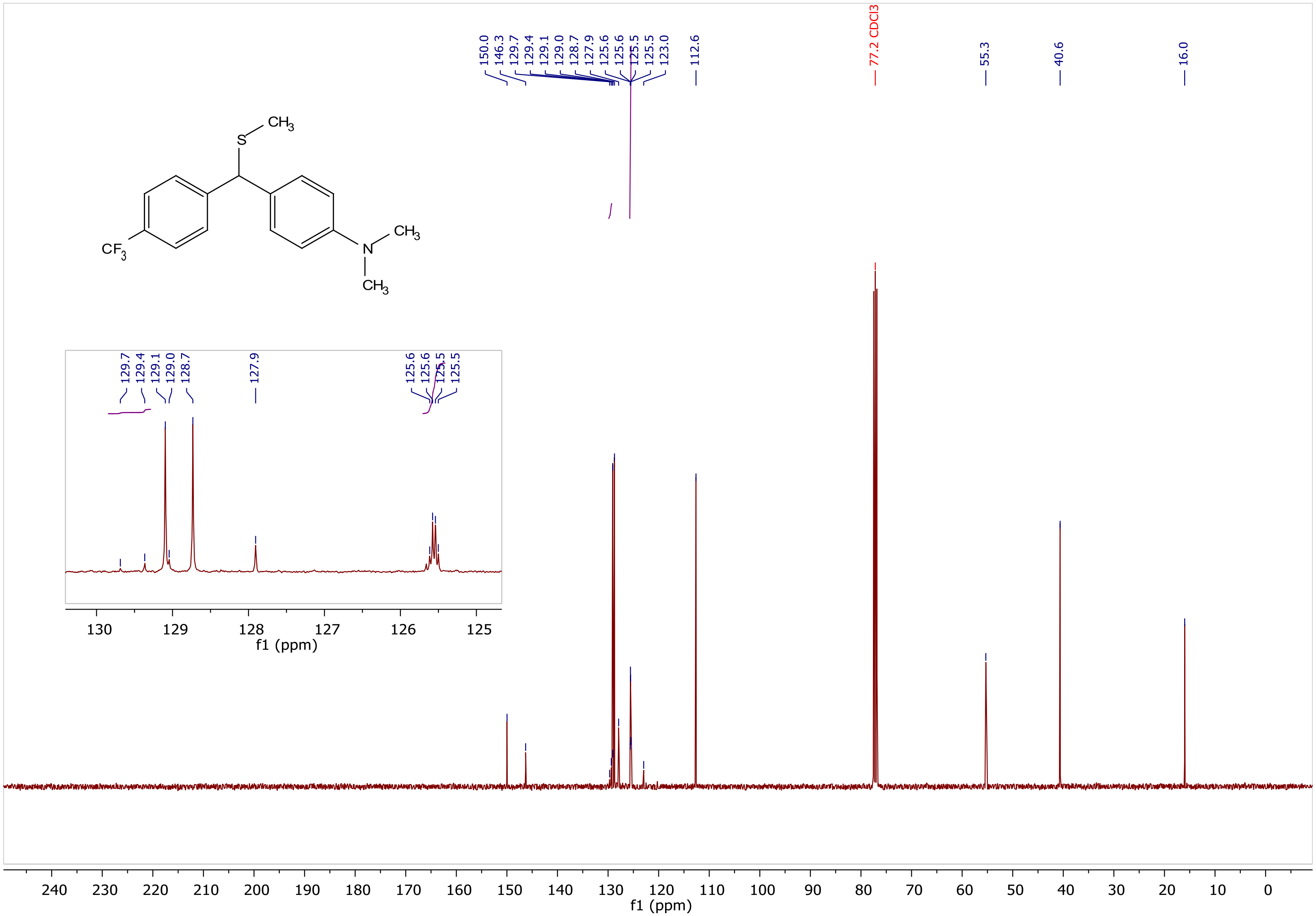

Figure S56. $^{13}C\{^{1}H\}$-NMR (101 MHz, $CDCl_3$) of compound **12d.**

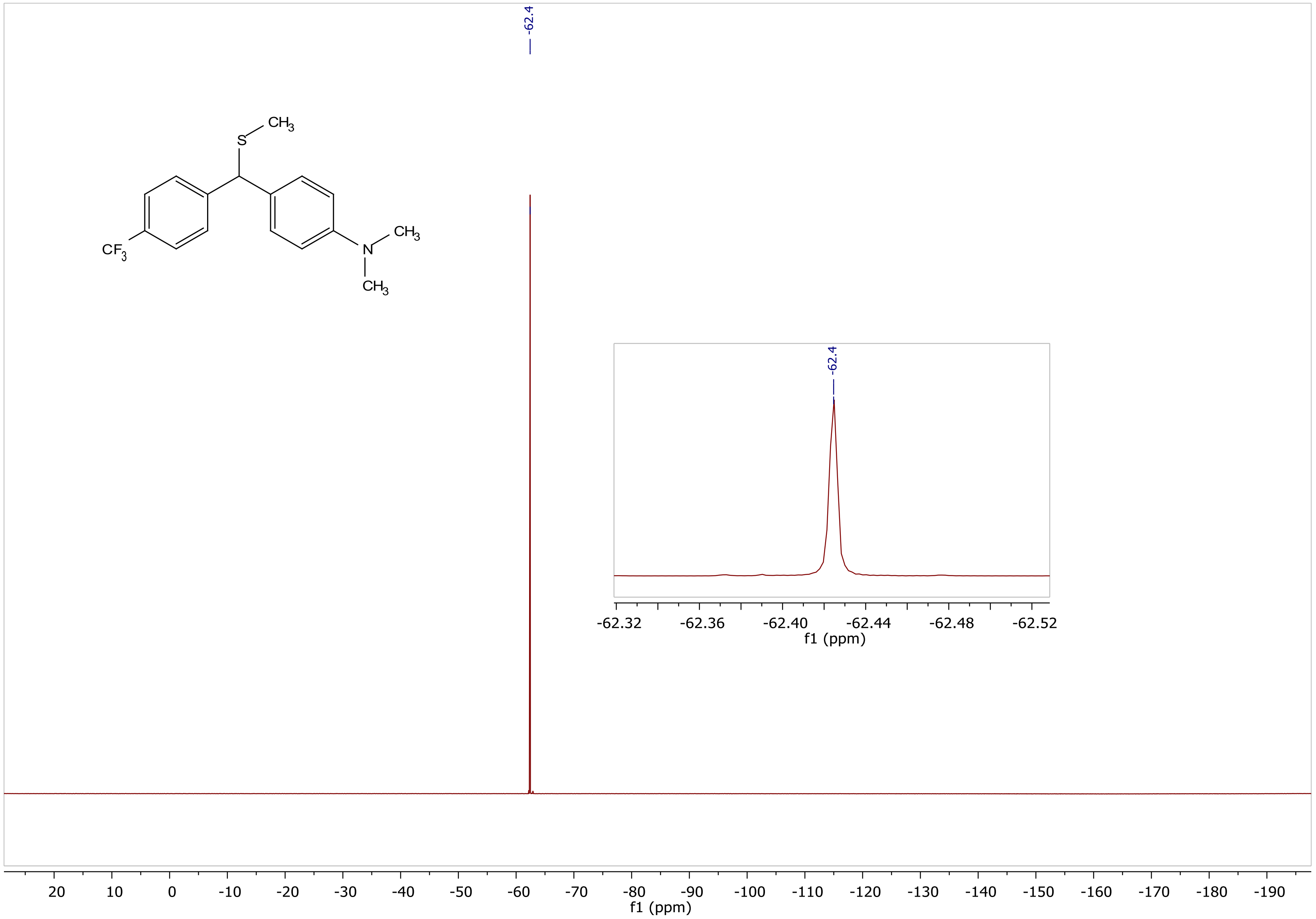

Figure S57. ^{19}F-NMR (376 MHz, $CDCl_3$) of compound **12d**.

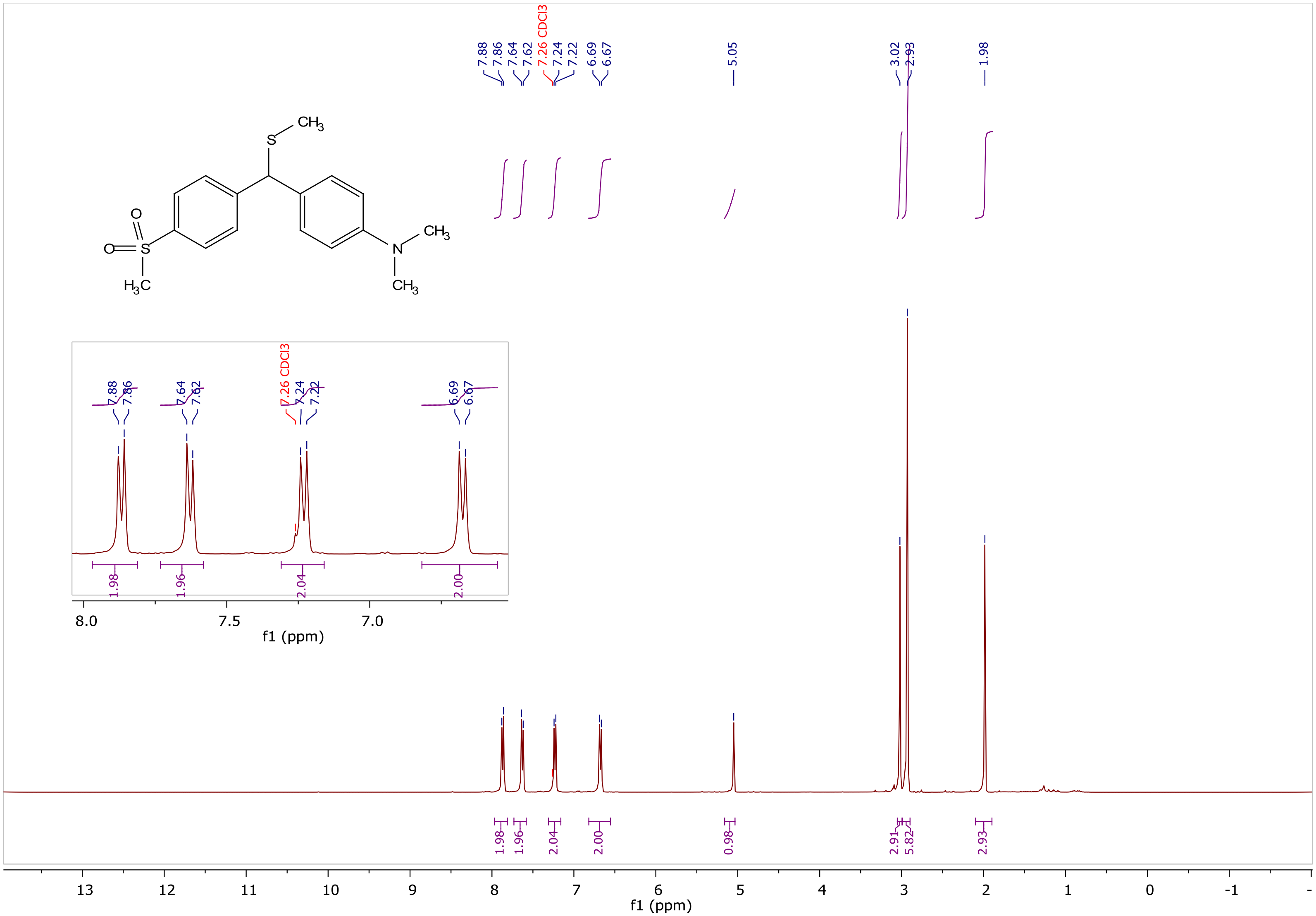

Figure S58. ^{1}H-NMR (400 MHz CDCl₃) of compound 1**3d.**

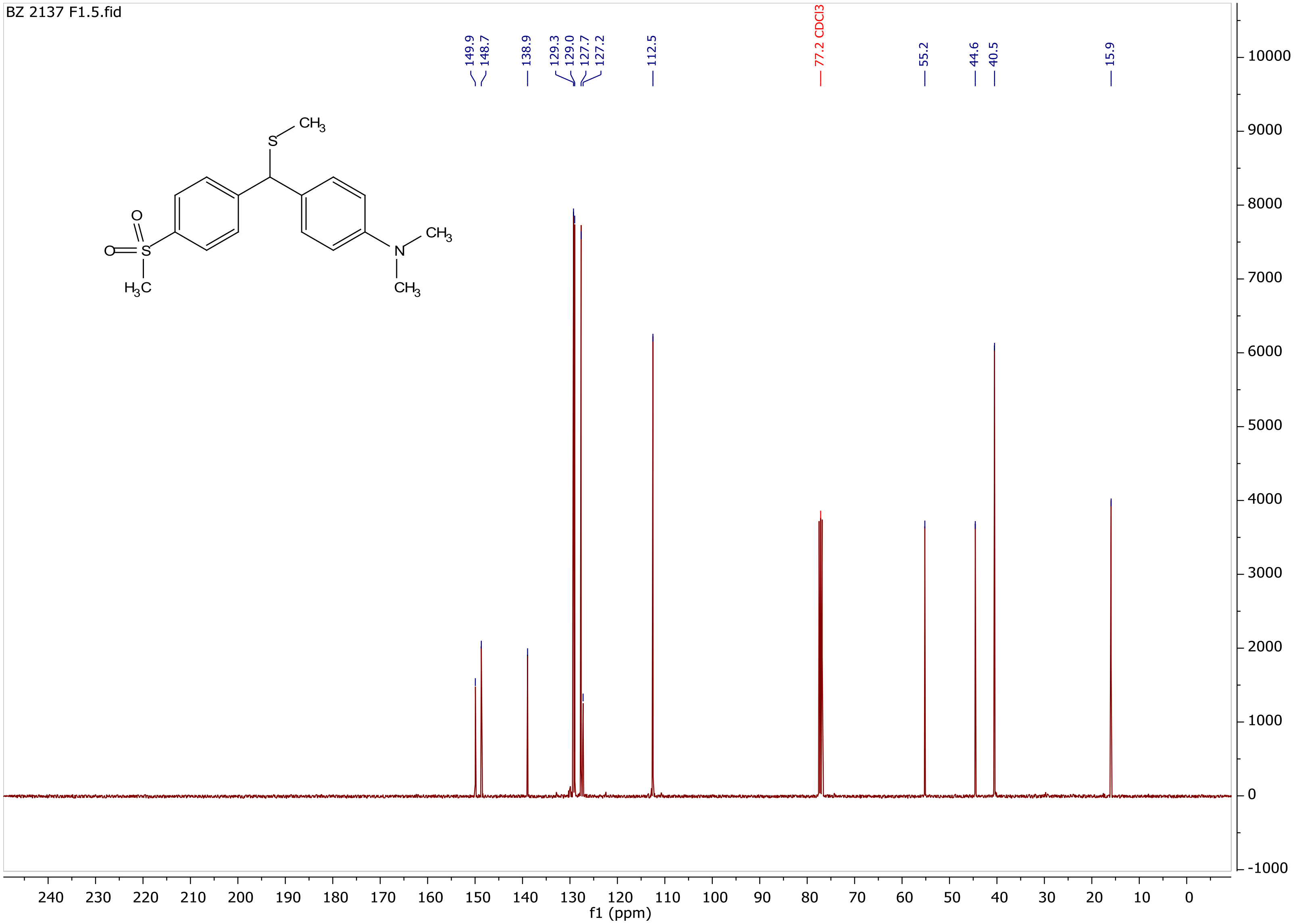

Figure S59. $^{13}C\{^1H\}$-NMR (101 MHz, $CDCl_3$) of compound **13d.**

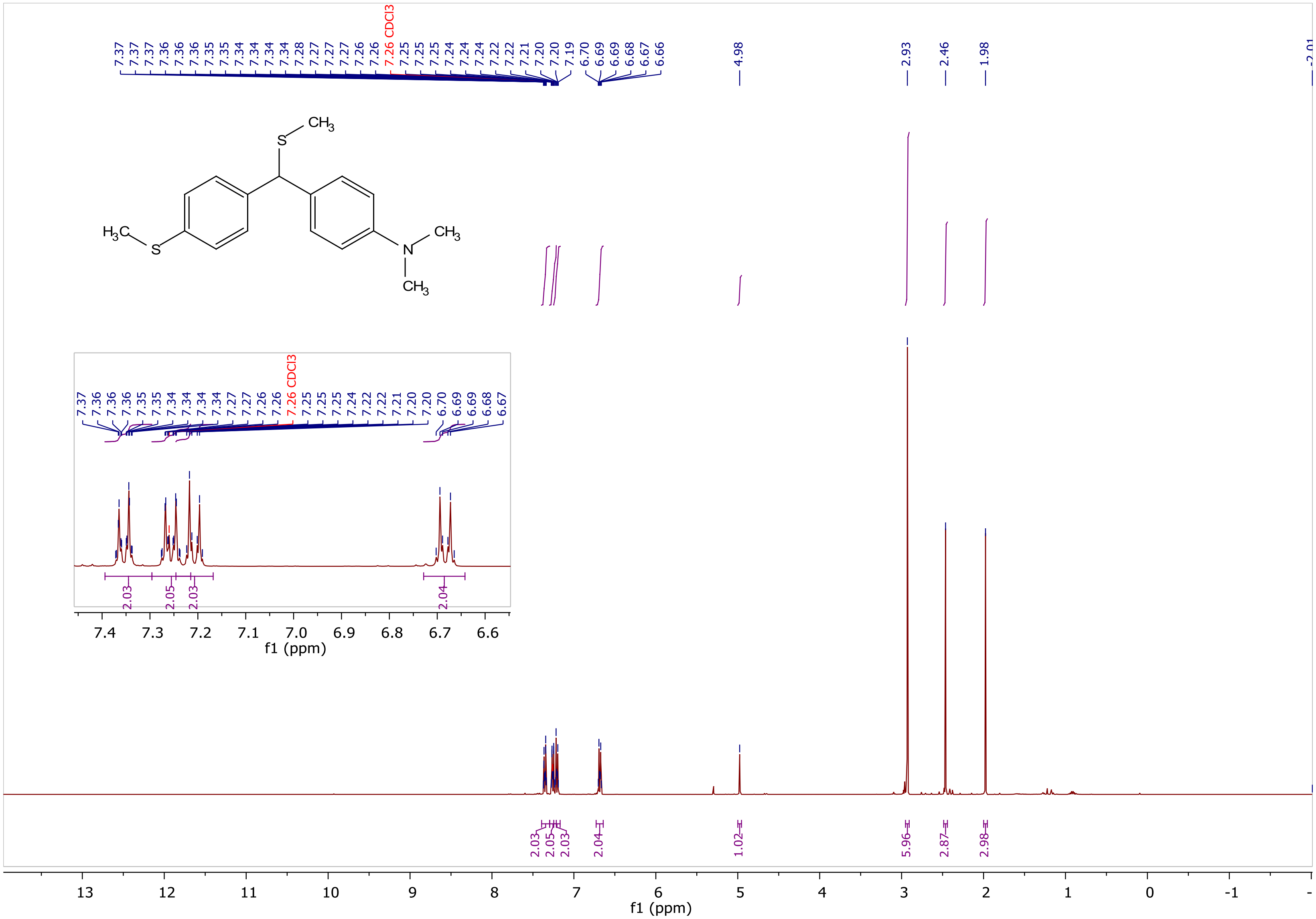

Figure S60. ^{1}H-NMR (400 MHz $CDCl_3$) of compound **14d**.

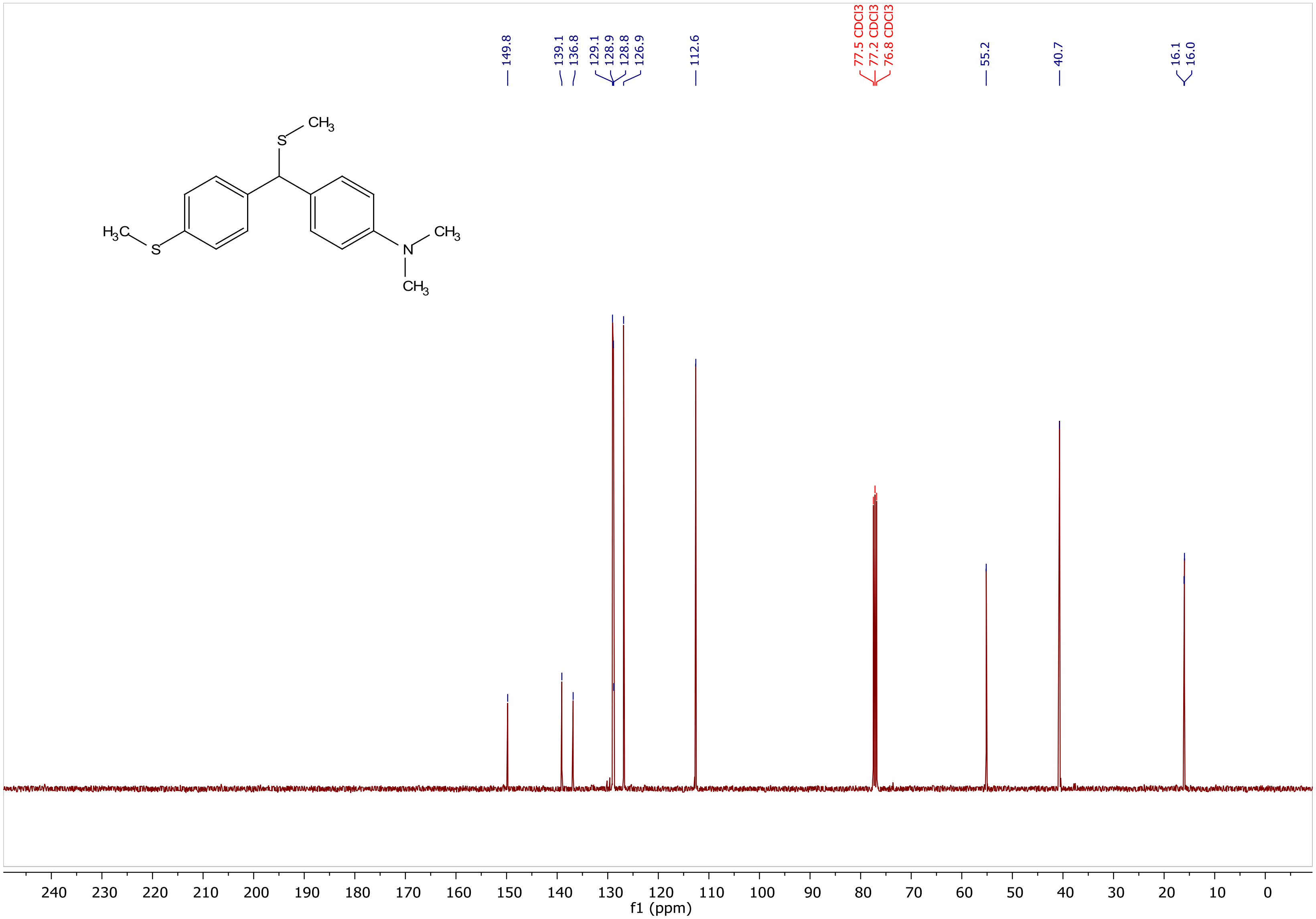

Figure S61. $^{13}C\{^{1}H\}$-NMR (101 MHz, $CDCl_3$) of compound **14d.**

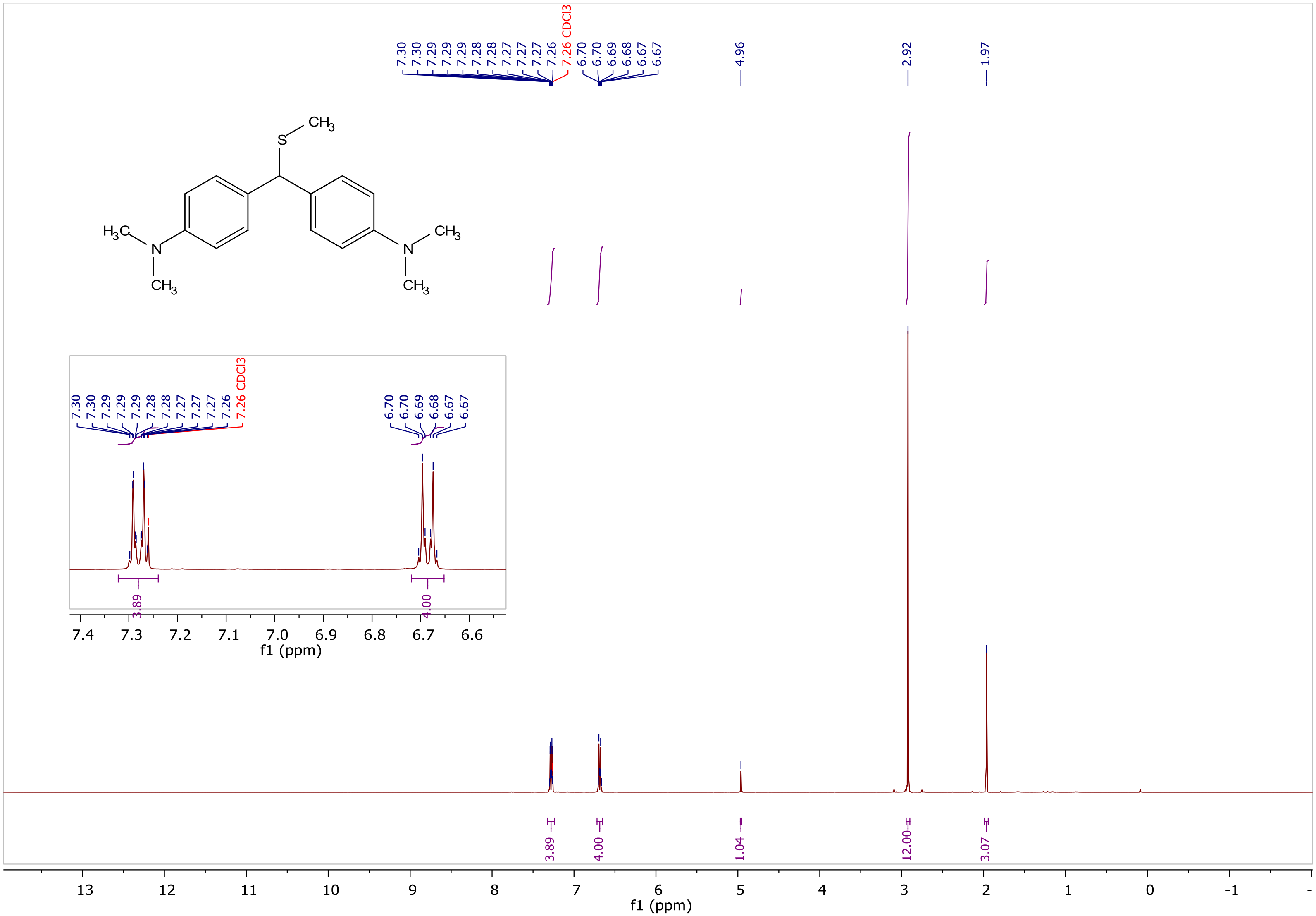

Figure S62. ^{1}H-NMR (400 MHz $CDCl_3$) of compound **15d.**

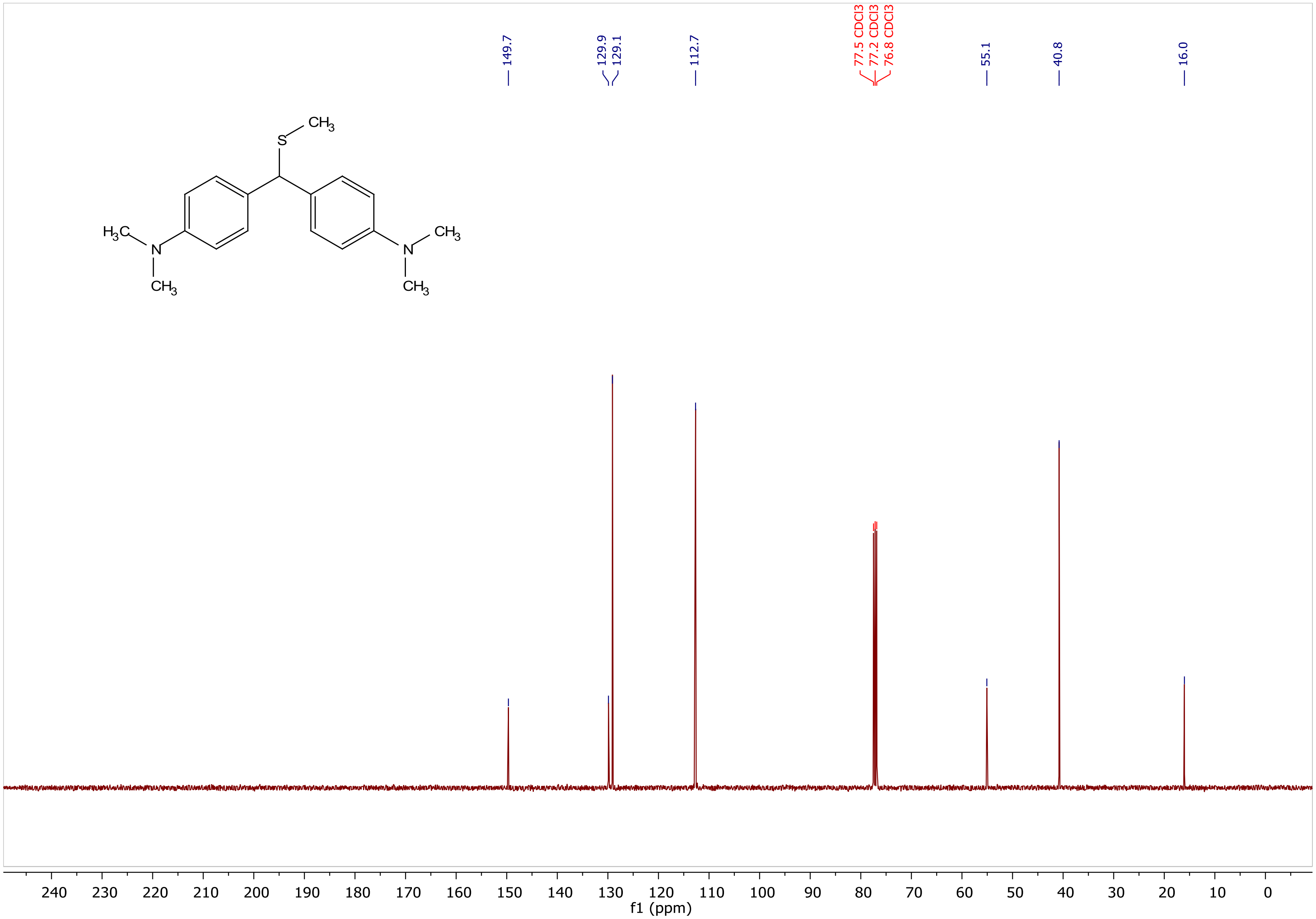

Figure S63. $^{13}C\{^{1}H\}$-NMR (101 MHz, $CDCl_3$) of compound **15d.**

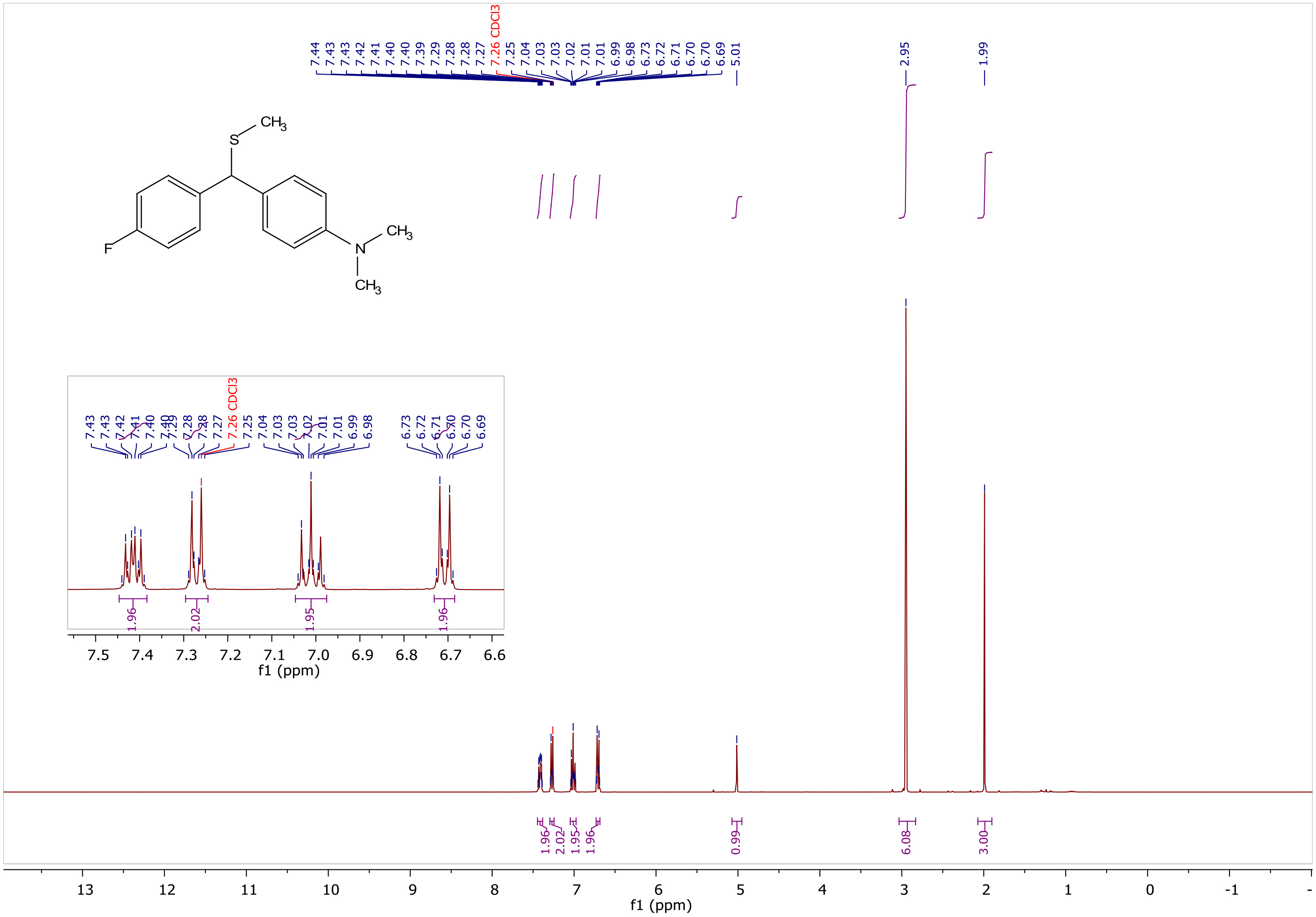

Figure S64. ^{1}H-NMR (400 MHz $CDCl_3$) of compound **16d.**

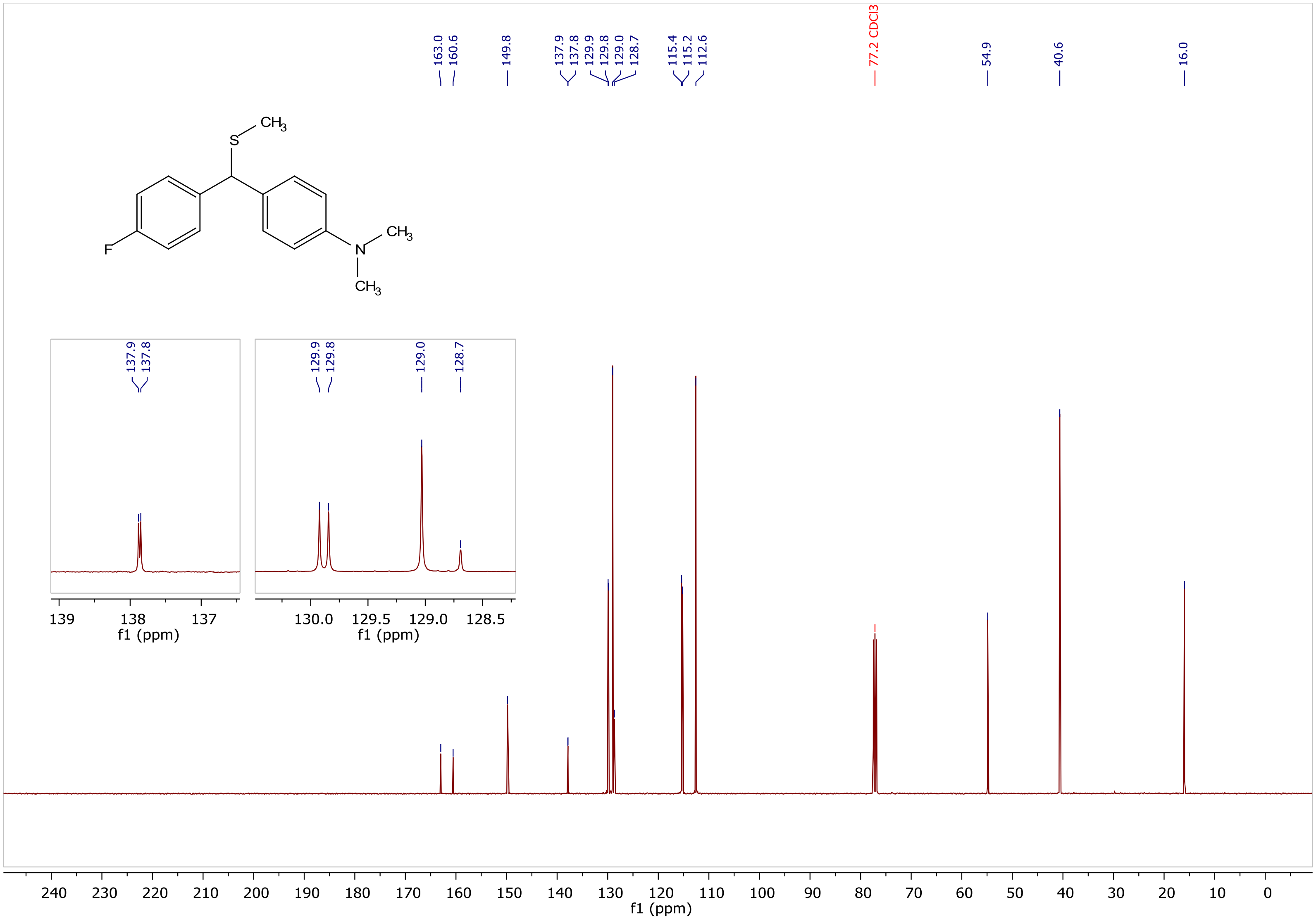

Figure S65. $^{13}C\{^{1}H\}$-NMR (101 MHz, $CDCl_3$) of compound **16d.**

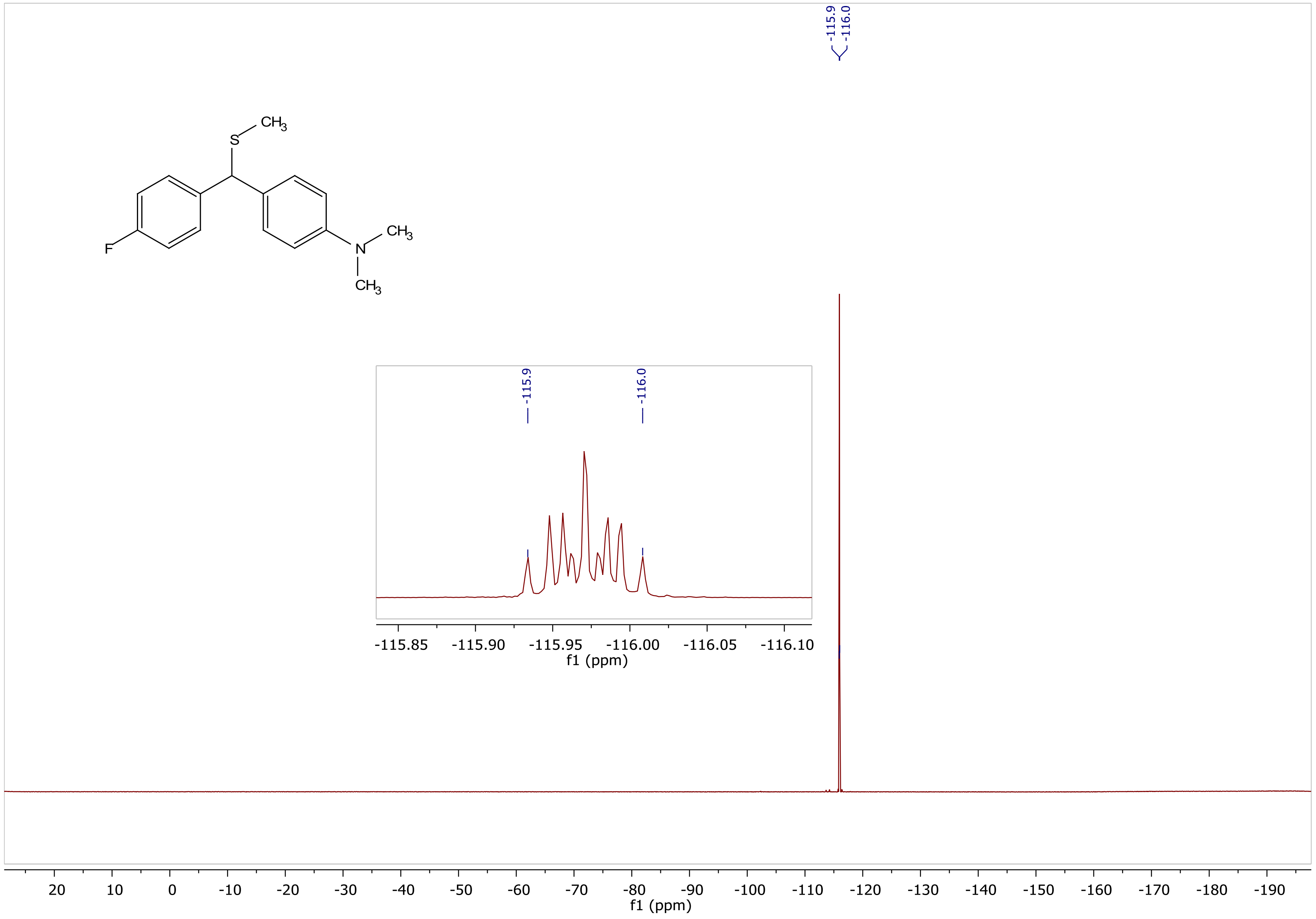

Figure S66. ^{19}F-NMR (376 MHz, $CDCl_3$) of compound **16d**.

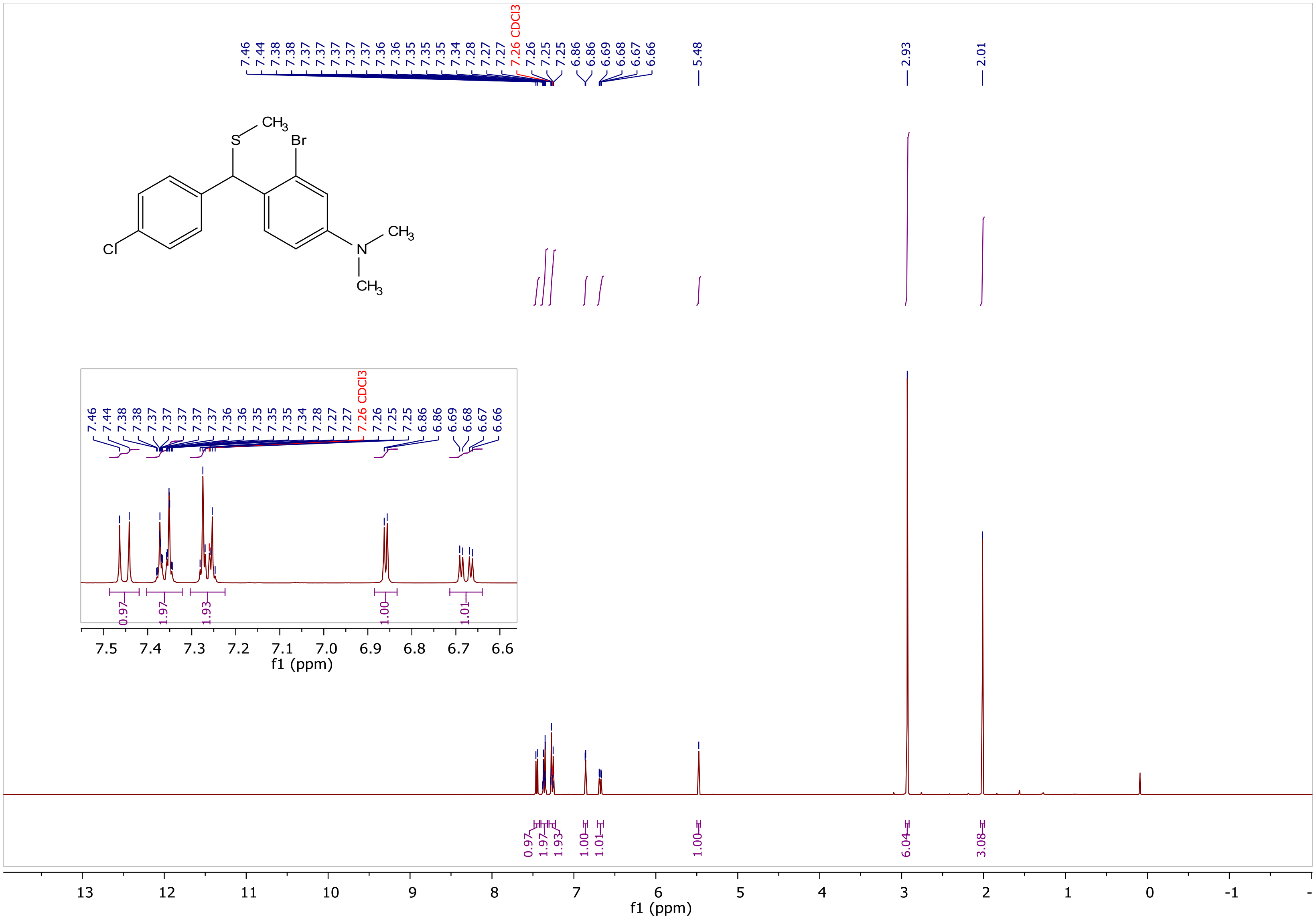

Figure S67. ^{1}H-NMR (400 MHz $CDCl_3$) of compound **17d.**

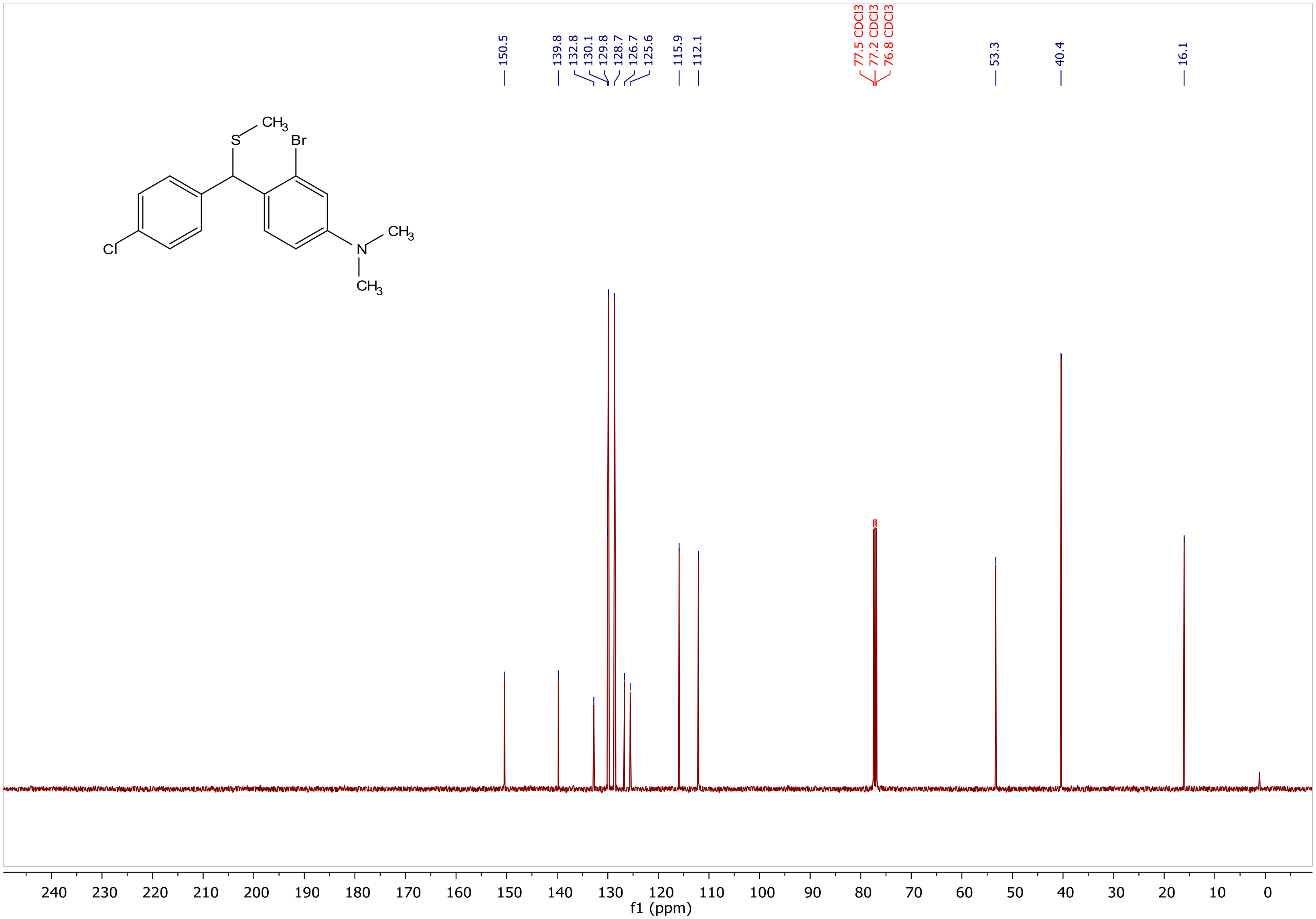

Figure S68. $^{13}C\{^{1}H\}$-NMR (101 MHz, $CDCl_3$) of compound **17d.**

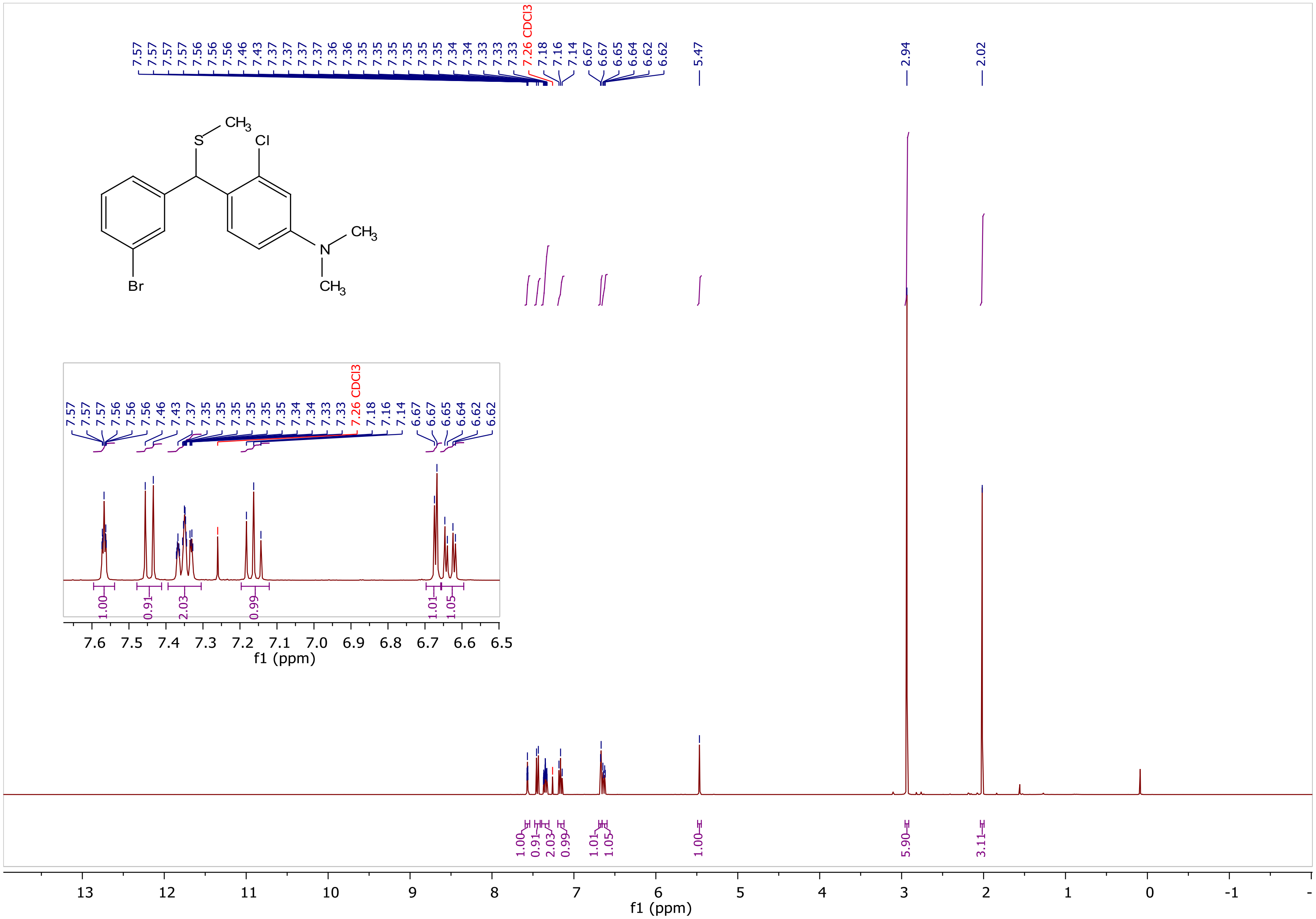

Figure S69. ^{1}H-NMR (400 MHz $CDCl_3$) of compound **18d.**

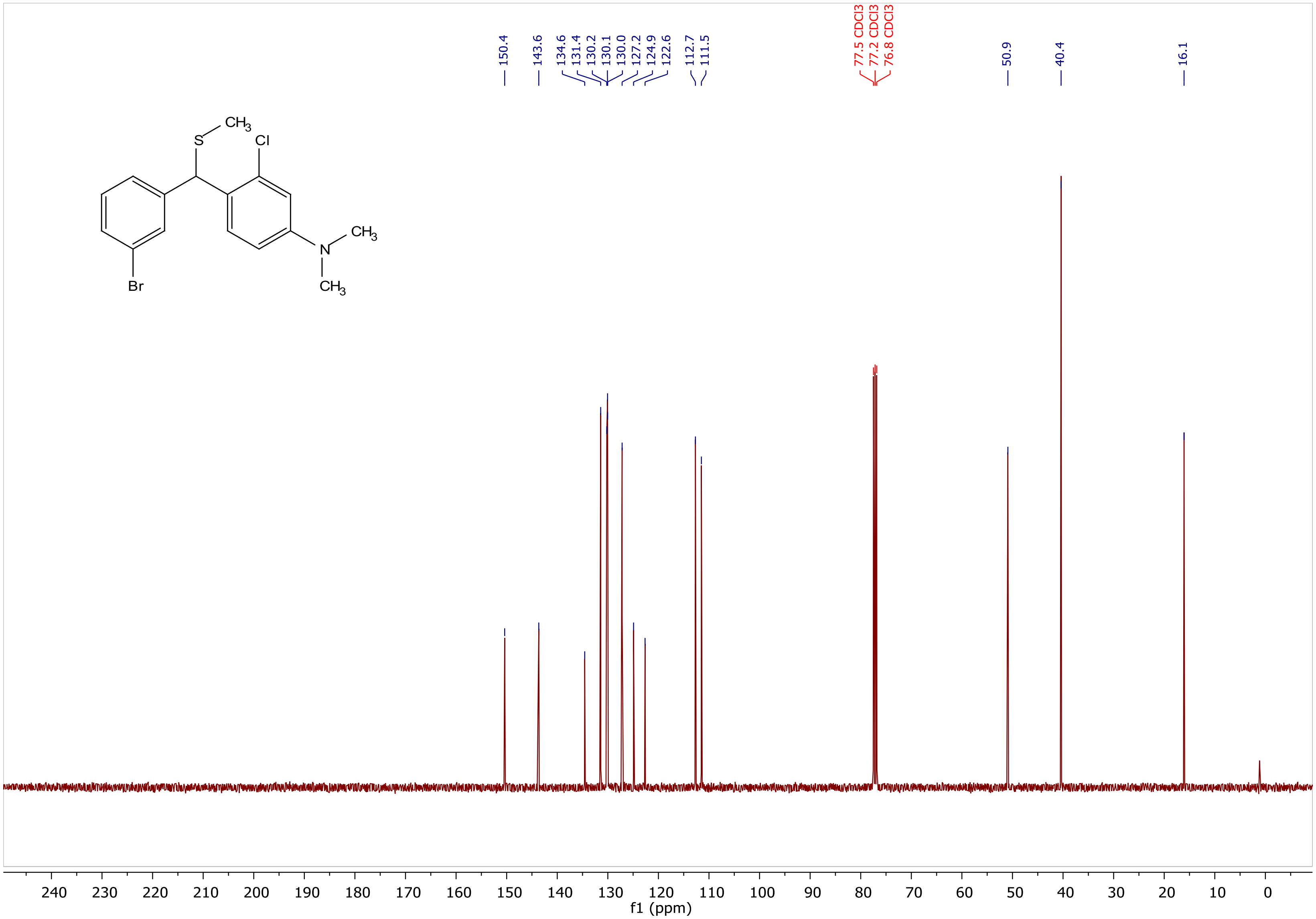

Figure S70. $^{13}C\{^{1}H\}$-NMR (101 MHz, $CDCl_3$) of compound **18d.**

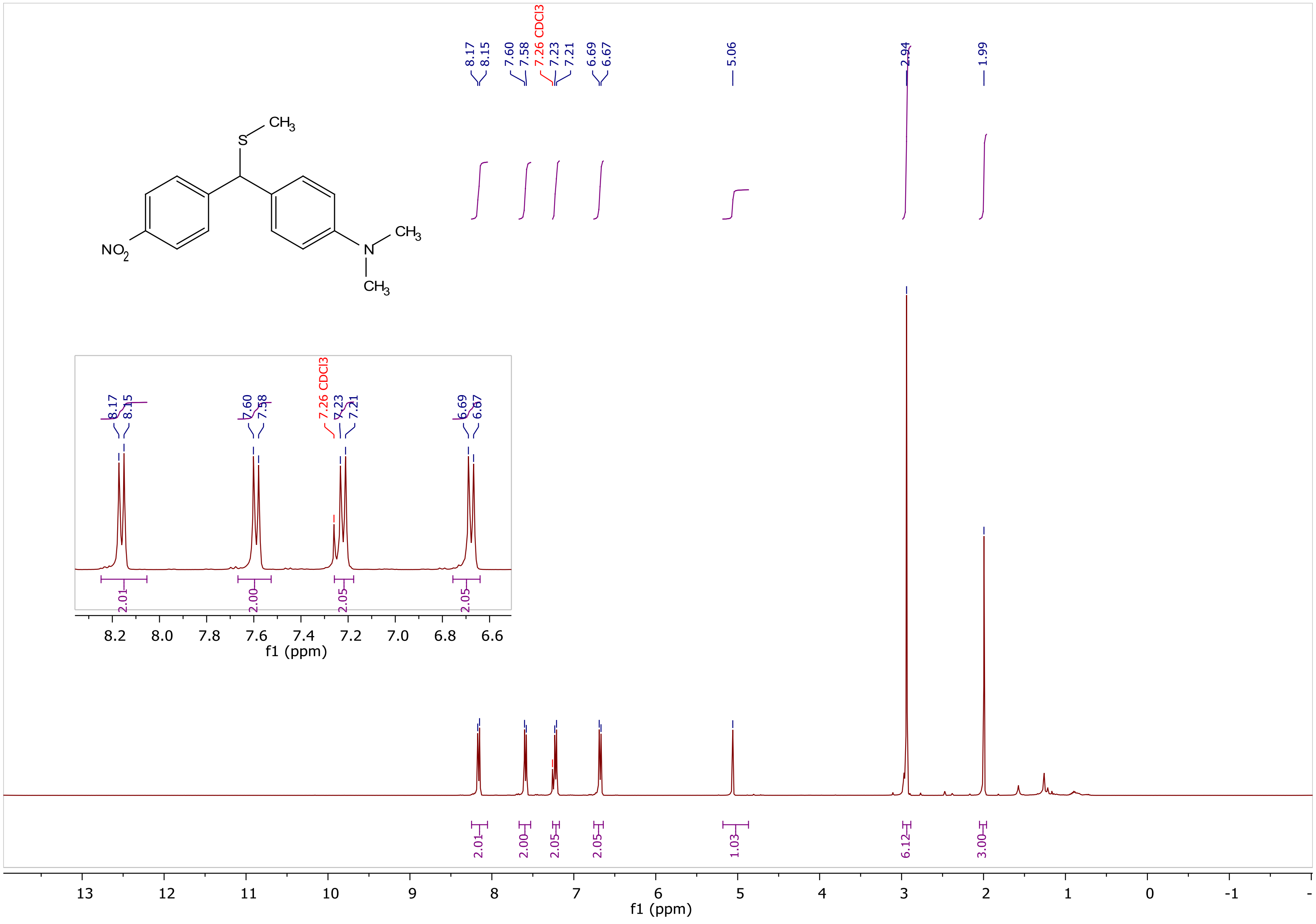

Figure S71. ^{1}H-NMR (400 MHz $CDCl_3$) of compound **19d.**

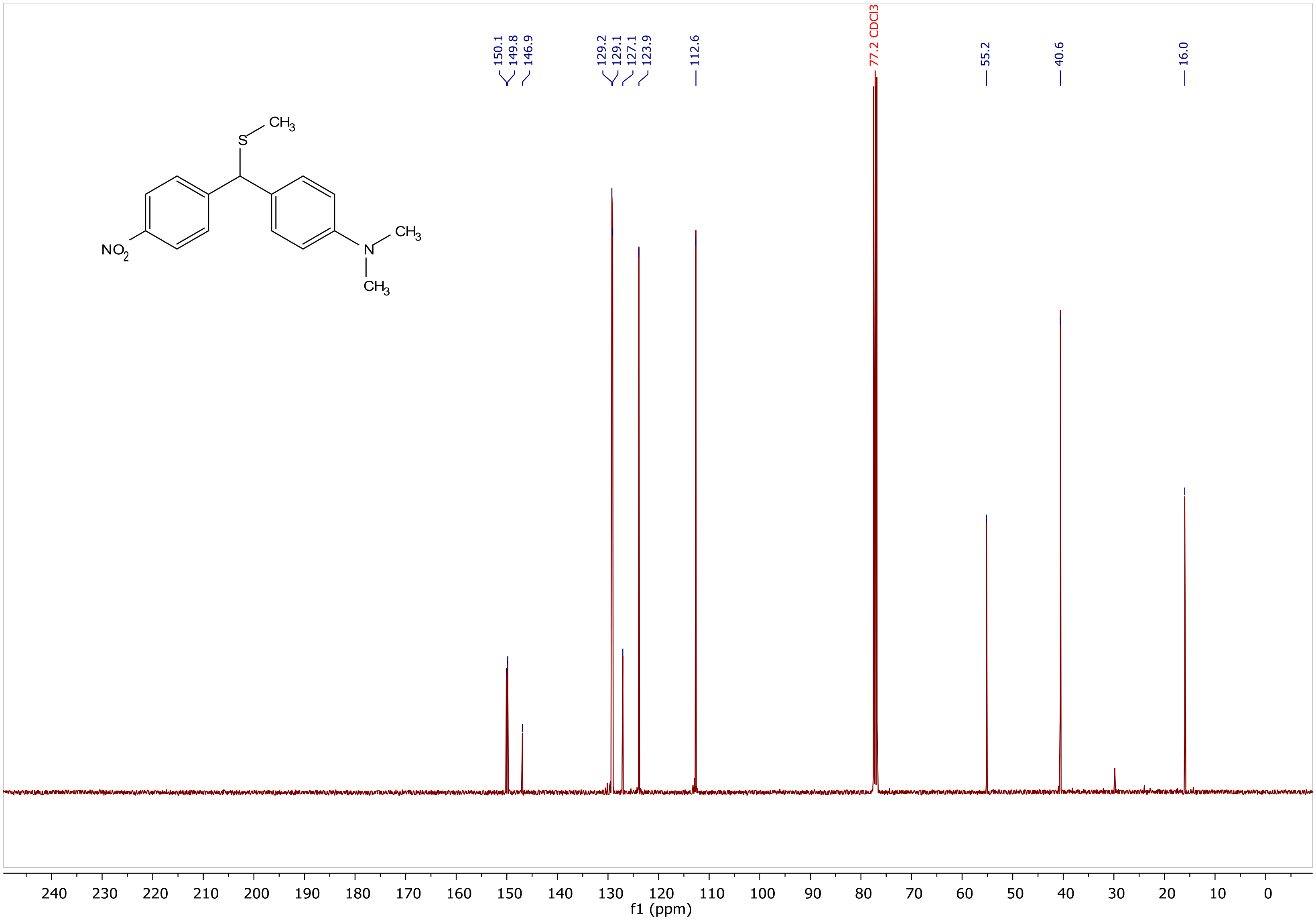

Figure S72. $^{13}C\{^1H\}$-NMR (101 MHz, $CDCl_3$) of compound **19d.**

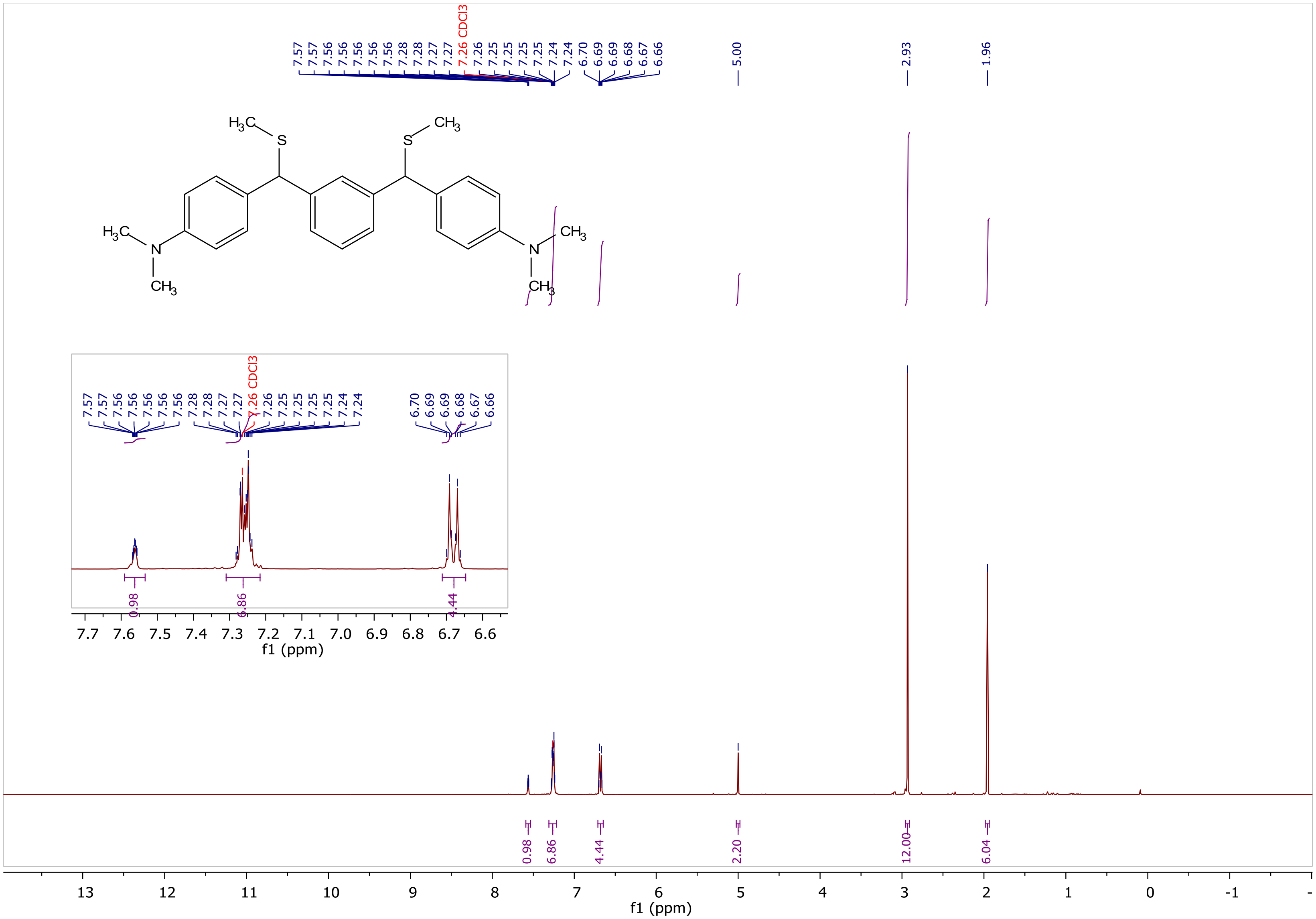

Figure S73. ^{1}H-NMR (400 MHz $CDCl_3$) of compound **20d.**

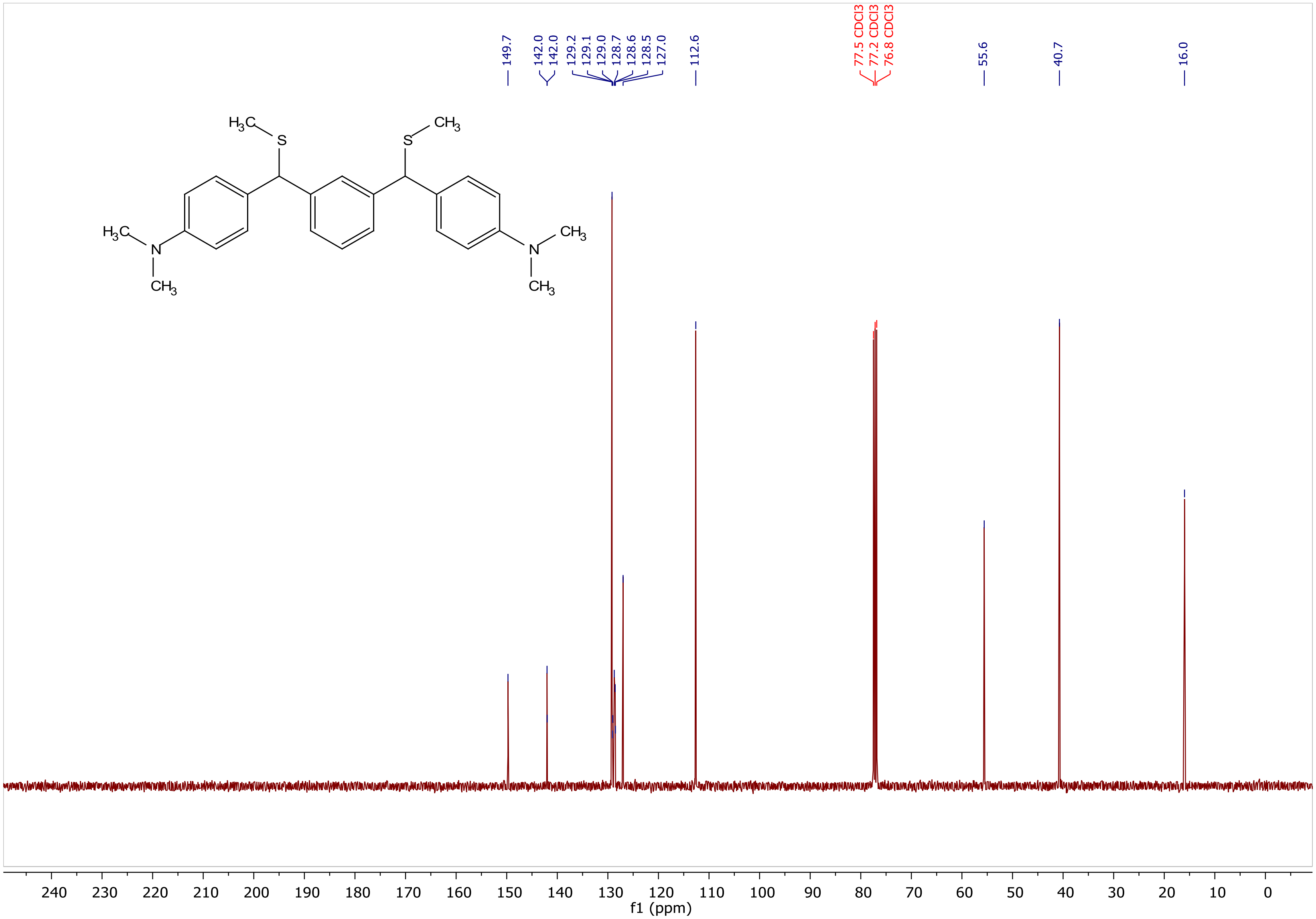

Figure S74. $^{13}C\{^{1}H\}$-NMR (101 MHz, $CDCl_3$) of compound **20d.**

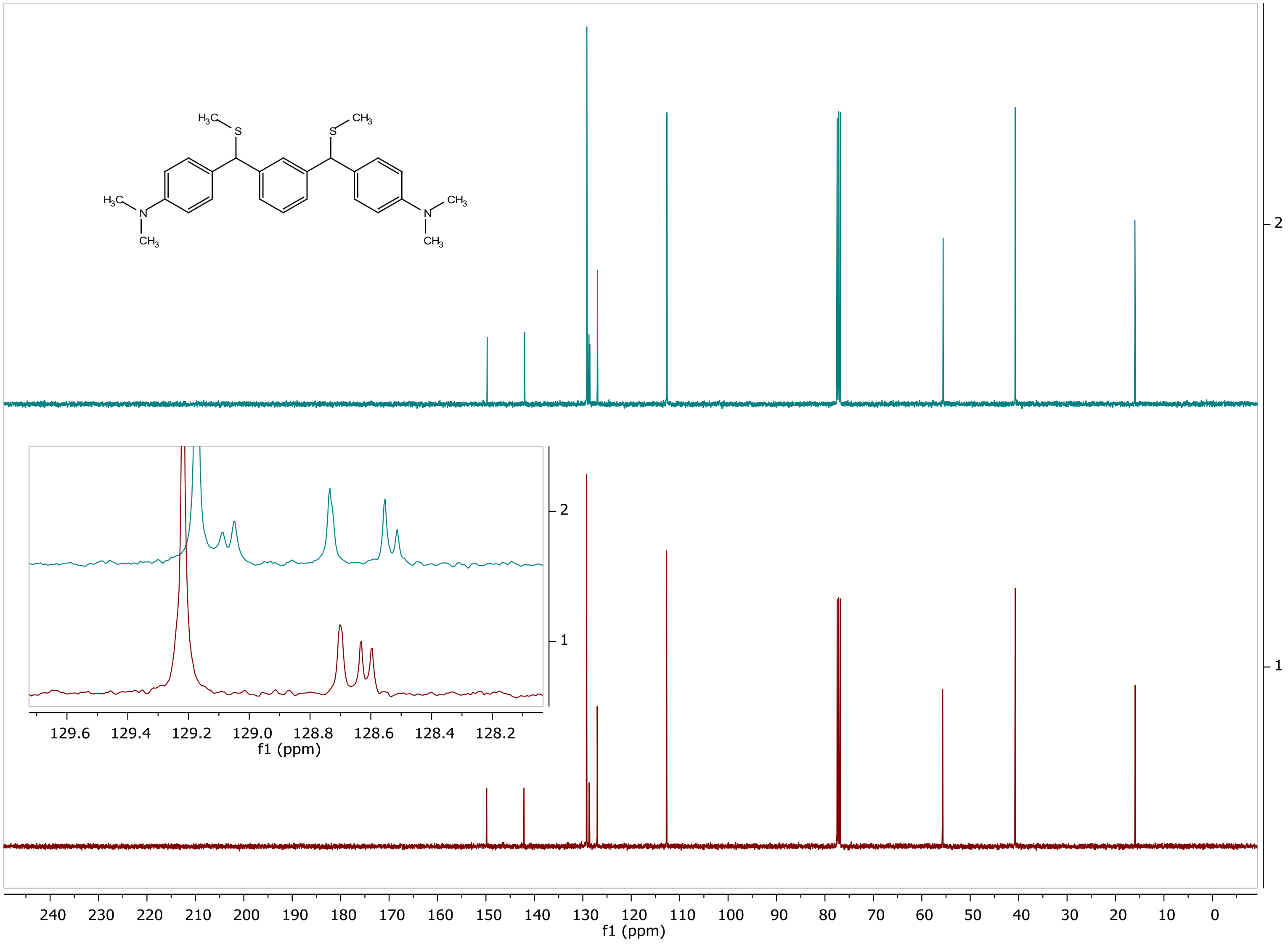

Figure S75. Effect of heating the $^{13}C\{^{1}H\}$-NMR (101 MHz, $CDCl_3$) sample of **20d**.
25ºC (top) and 40ºC (bottom) suggests that the extra peaks observed are from different configurations due to hindered rotation.

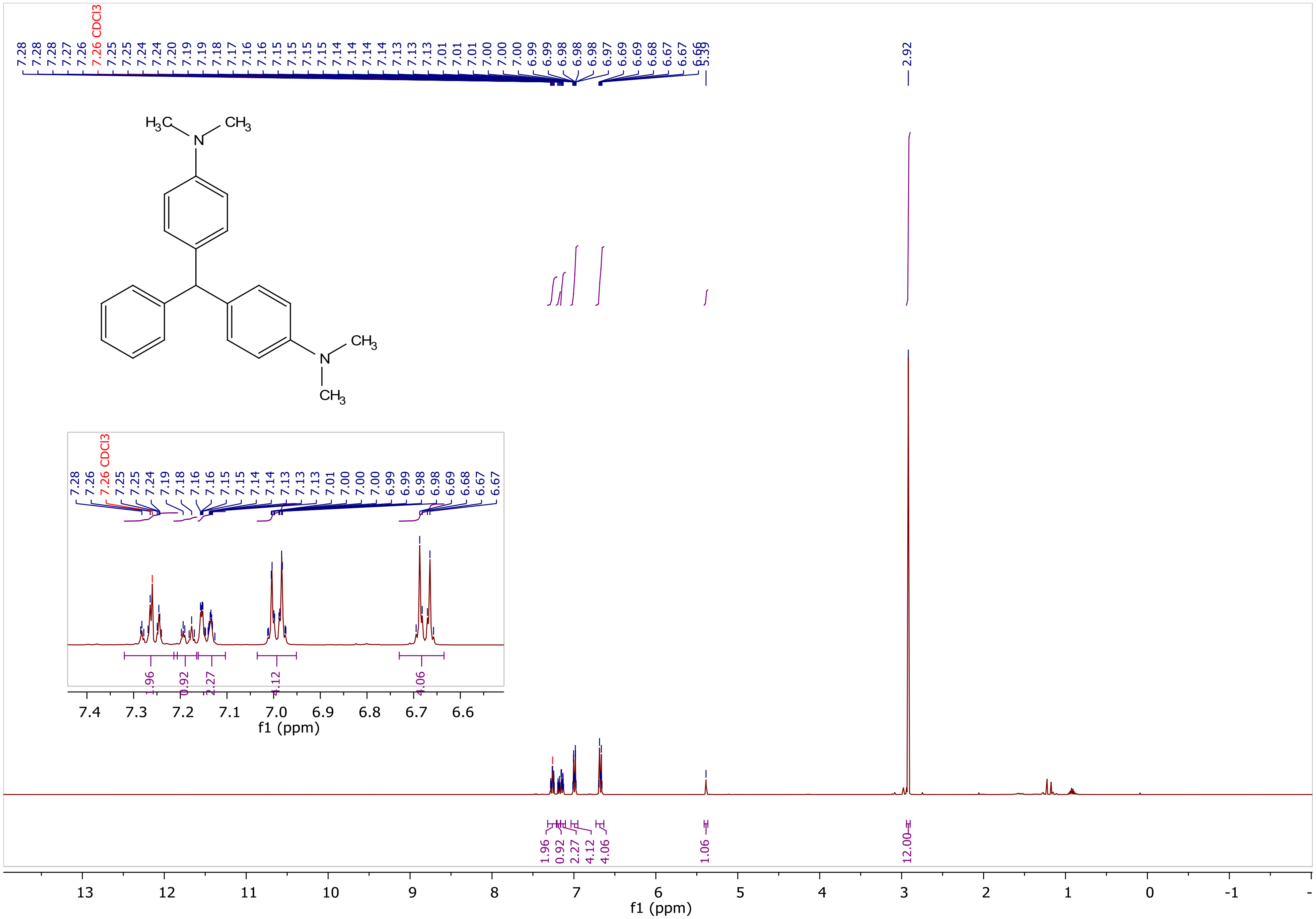

Figure S76. ^{1}H-NMR (400 MHz $CDCl_3$) of compound **1f.**

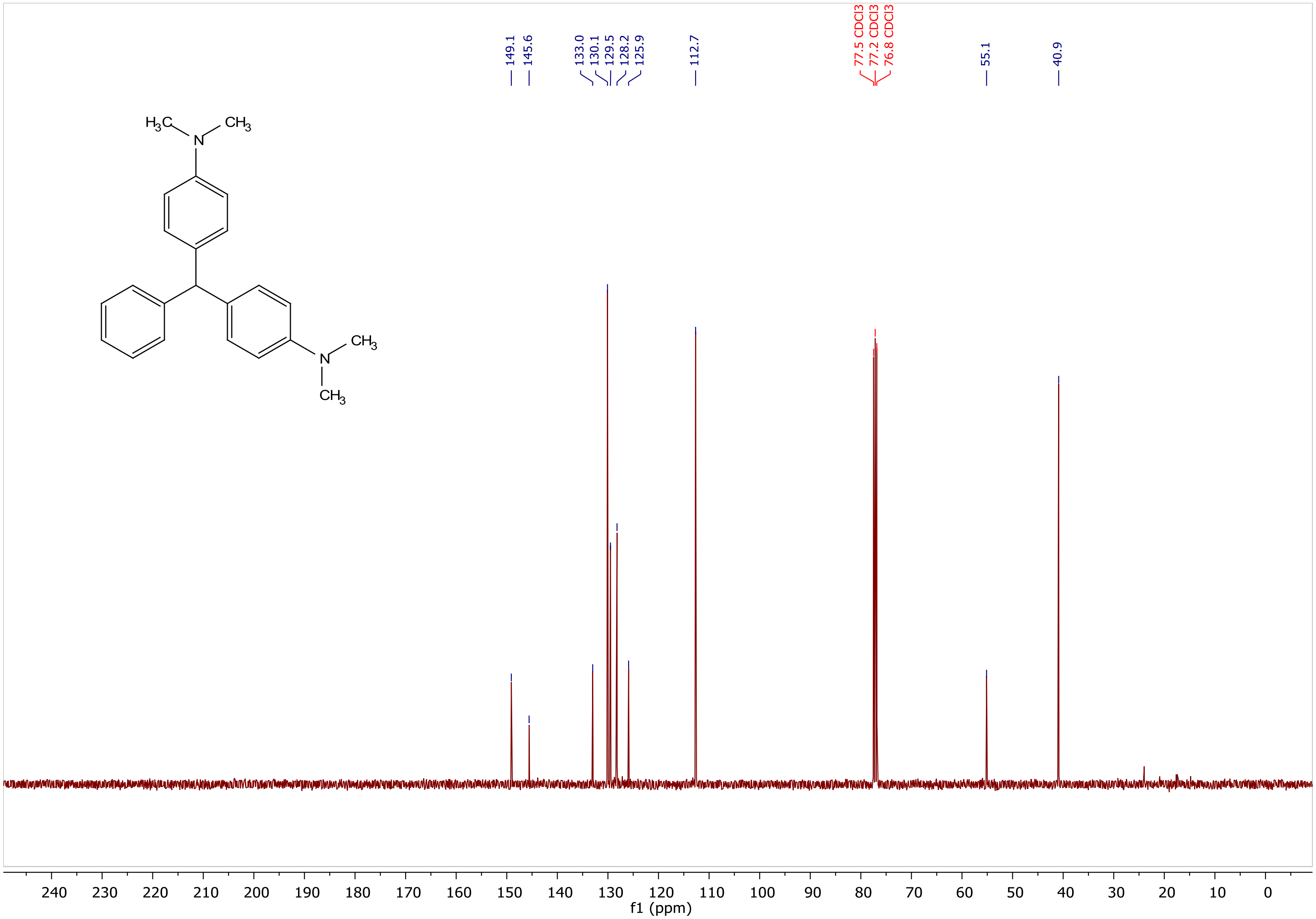

Figure S77. $^{13}C\{^{1}H\}$-NMR (101 MHz, $CDCl_3$) of compound **1f.**

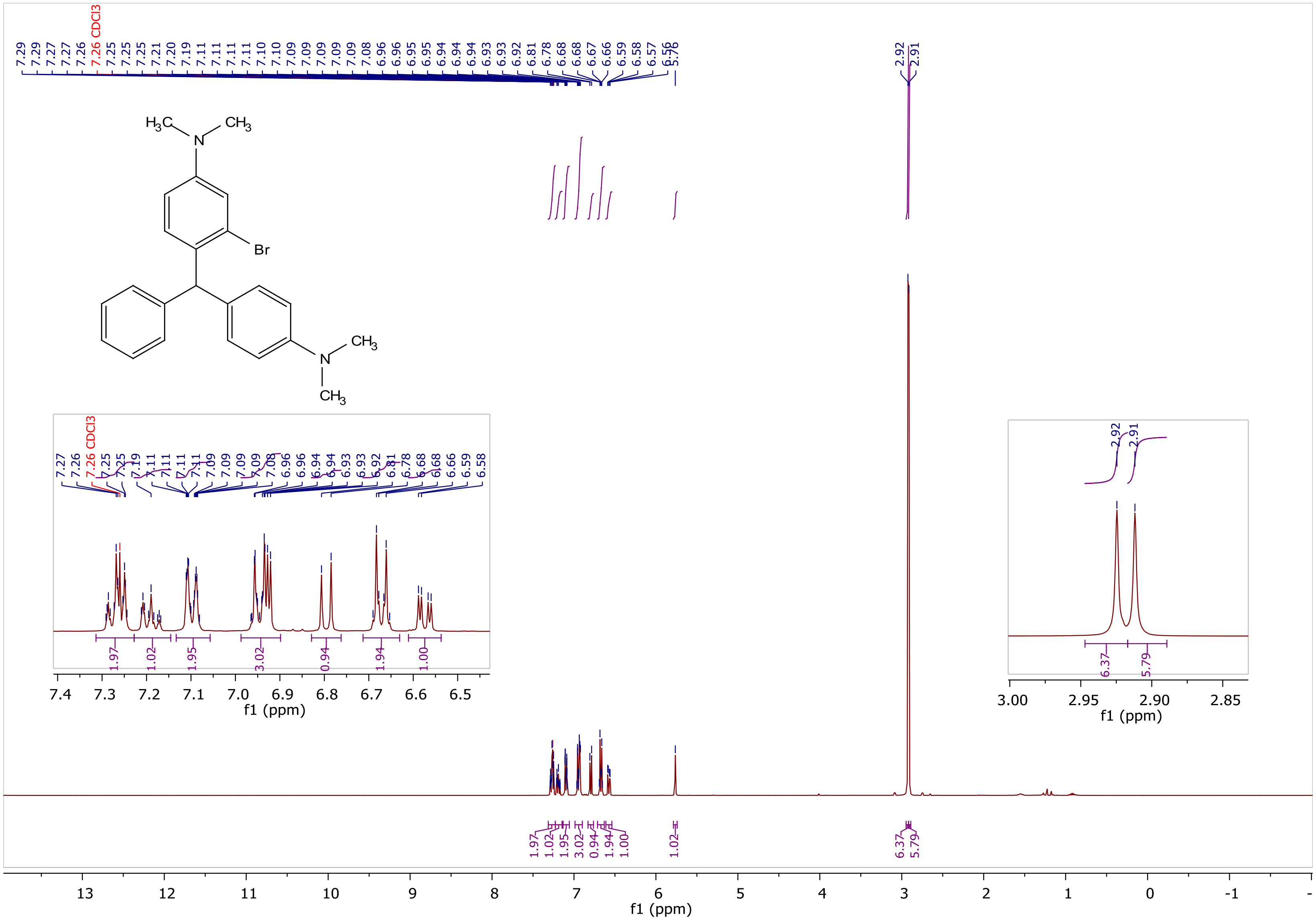

Figure S78. ^{1}H-NMR (400 MHz $CDCl_3$) of compound **1g.**

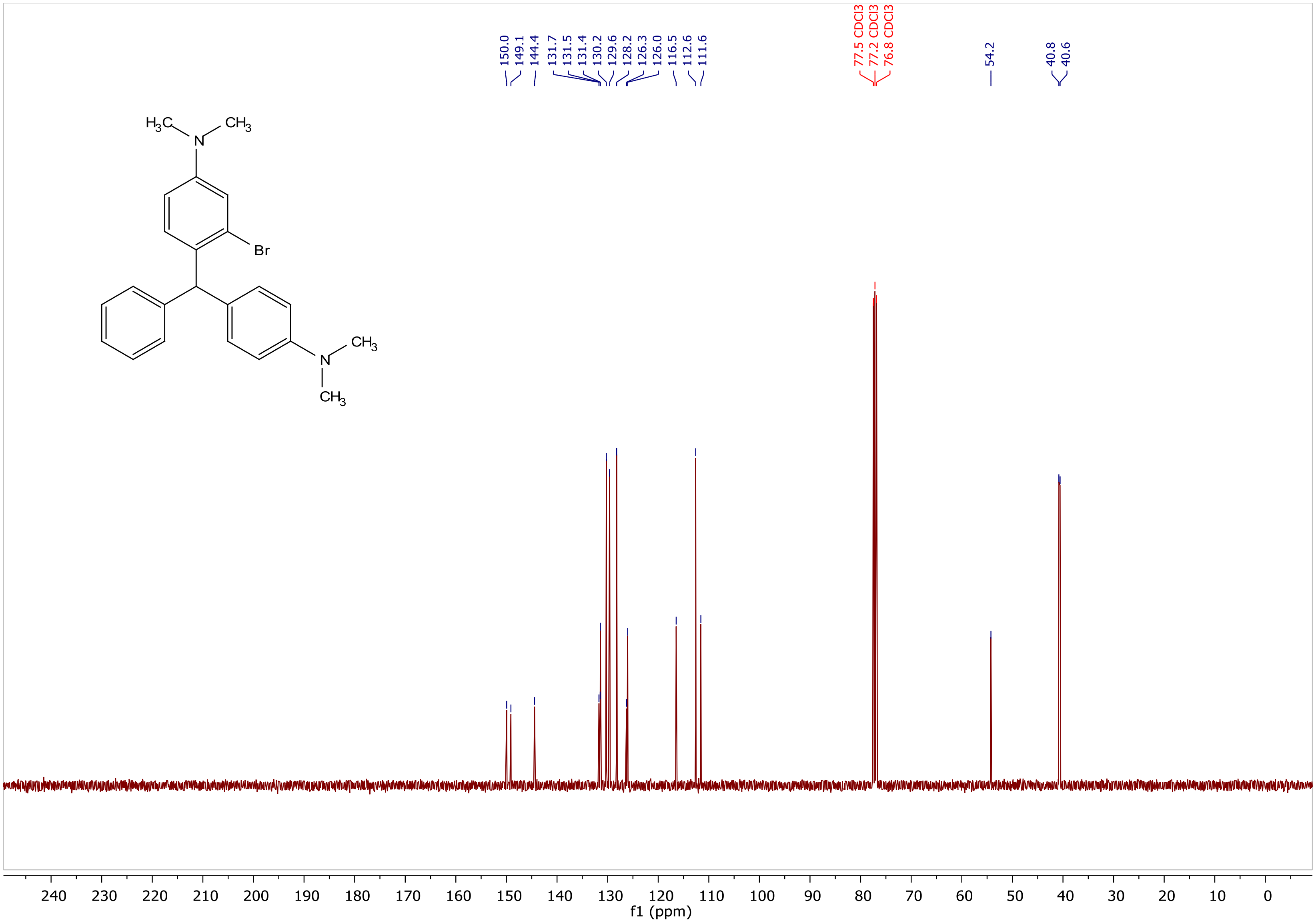

Figure S79. $^{13}C\{^{1}H\}$-NMR (101 MHz, $CDCl_3$) of compound **1g.**

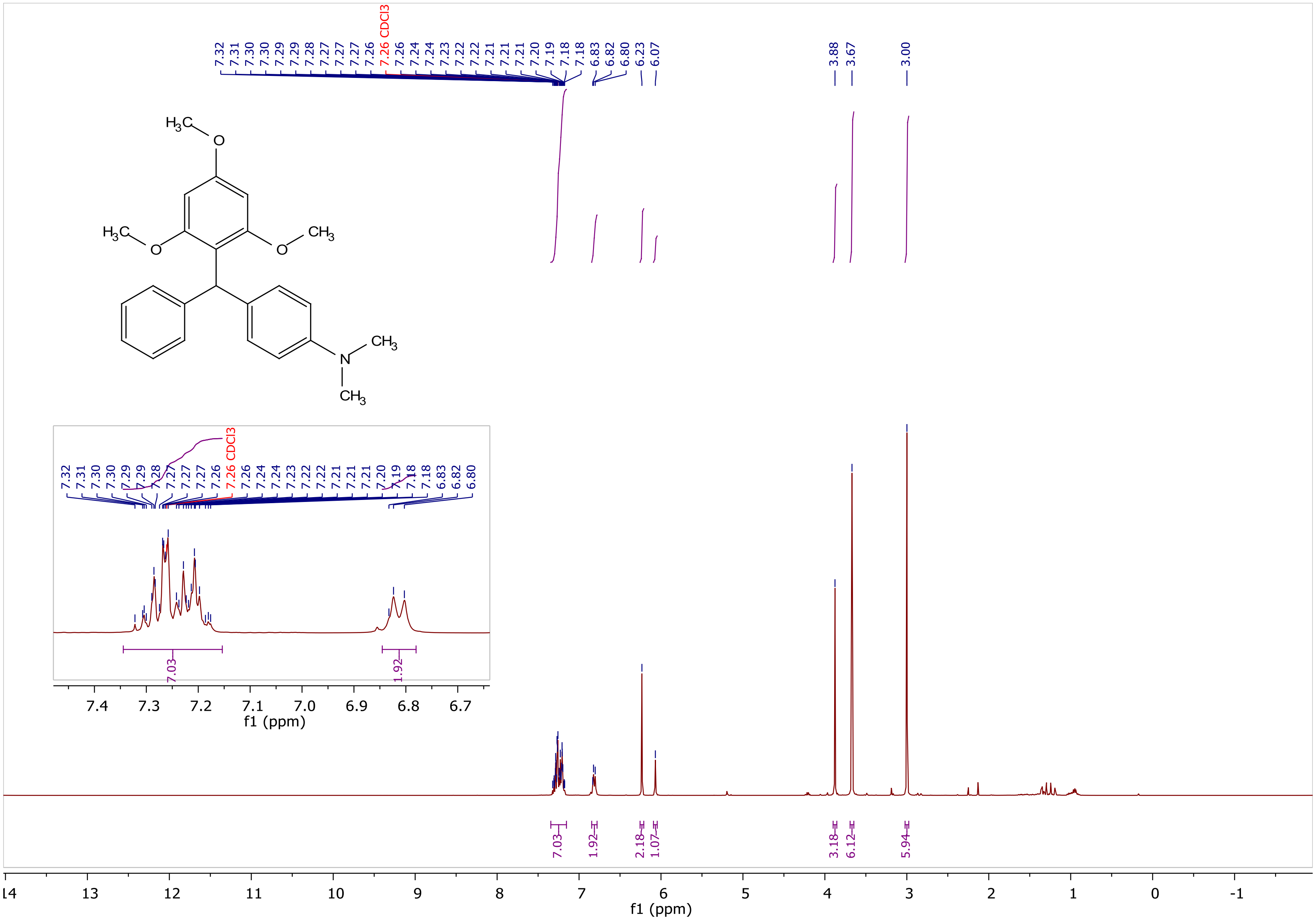

Figure S80. ^{1}H-NMR (400 MHz $CDCl_3$) of compound **1h.**

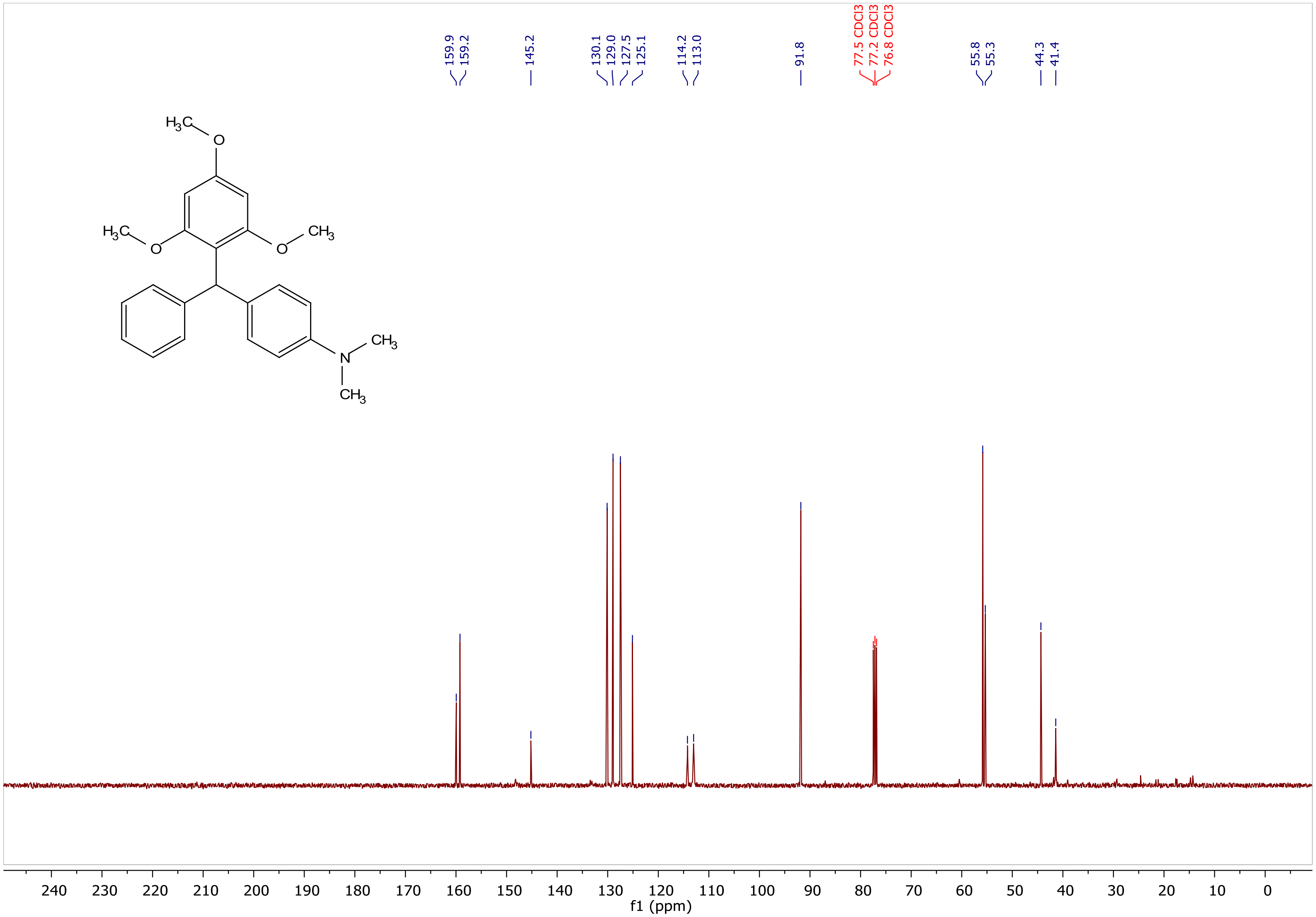

Figure S81. $^{13}C\{^1H\}$-NMR (101 MHz, $CDCl_3$) of compound **1h.**

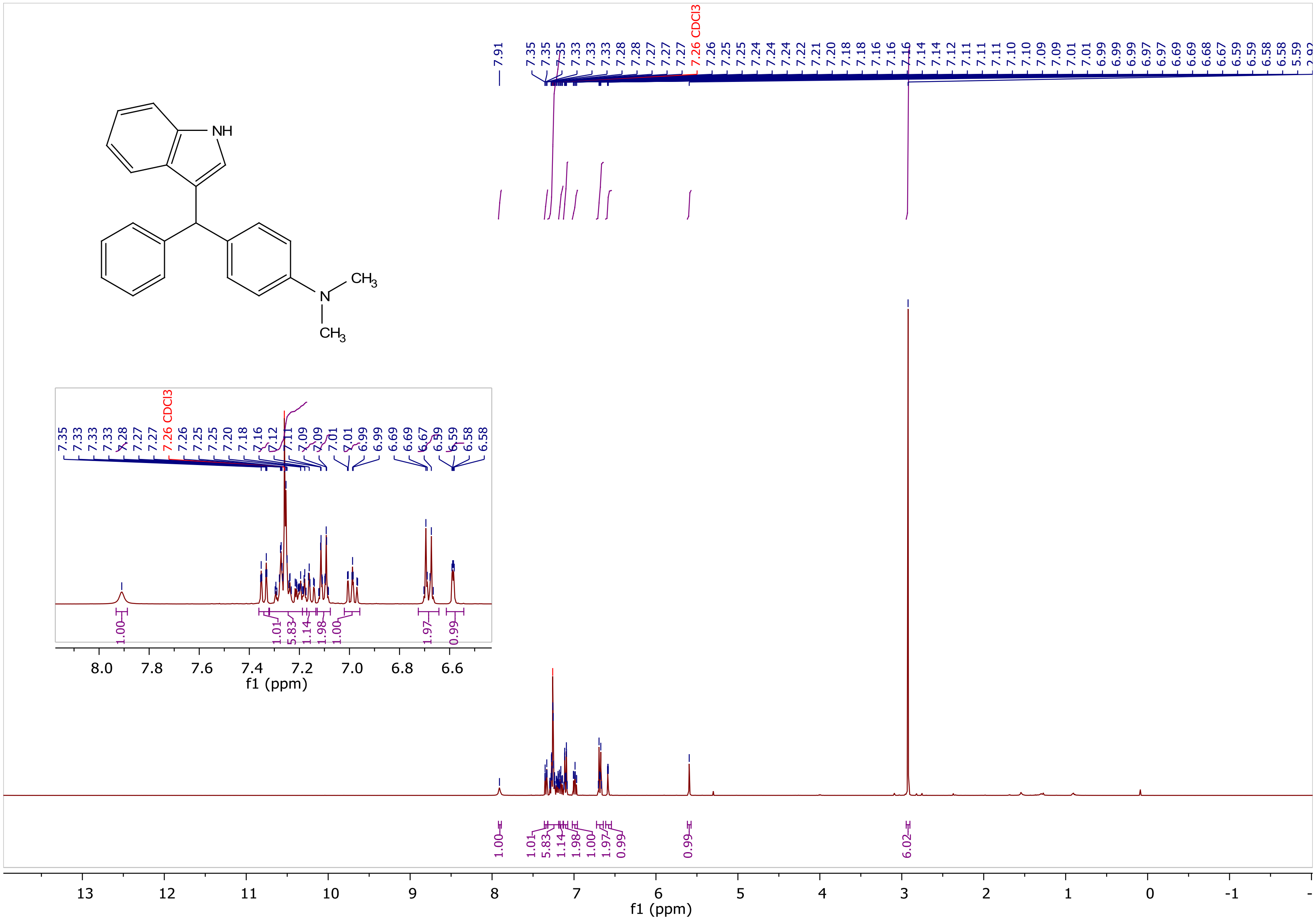

Figure S82. ^{1}H-NMR (400 MHz $CDCl_3$) of compound **1i.**

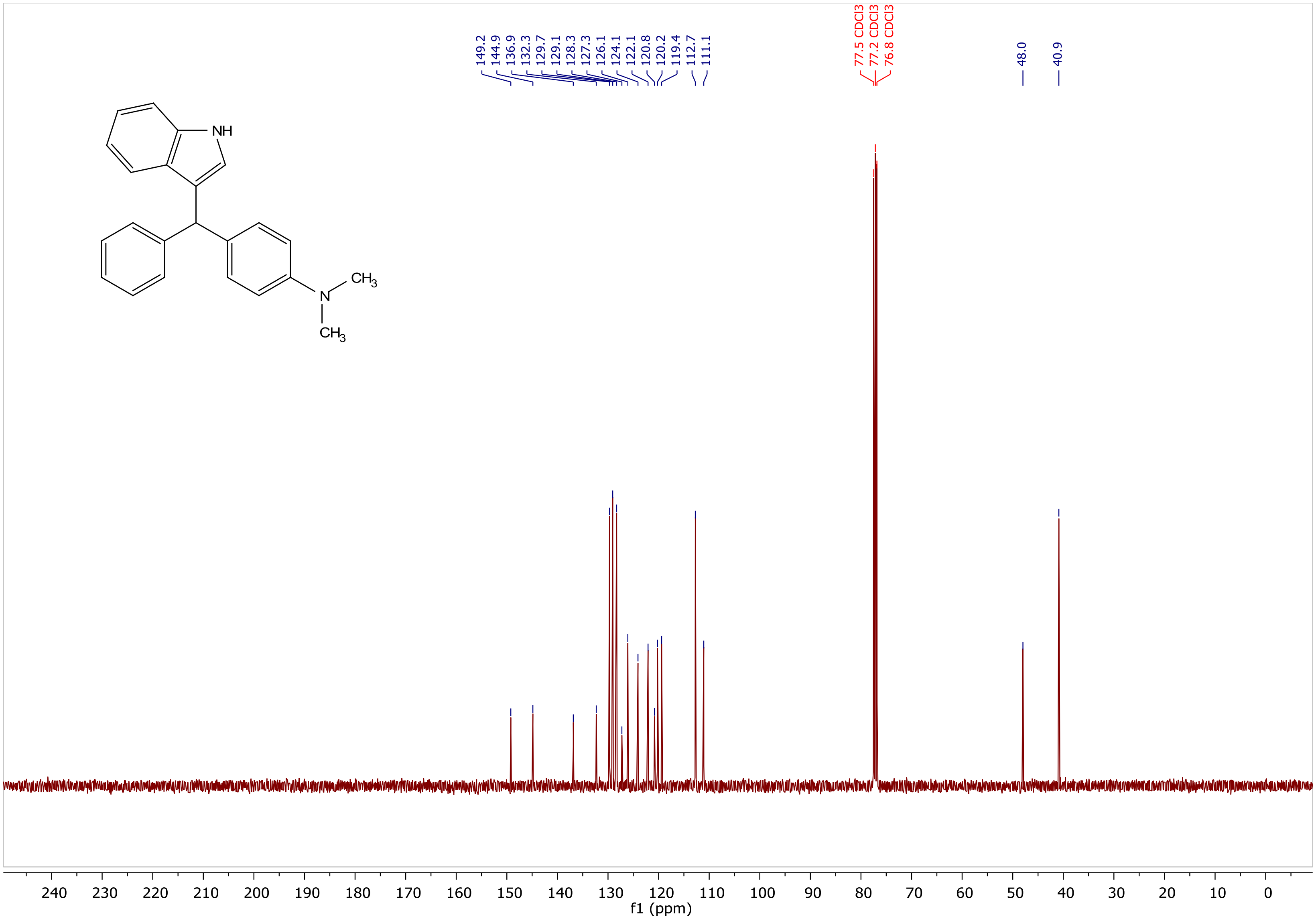

Figure S83. $^{13}C\{^1H\}$-NMR (101 MHz, $CDCl_3$) of compound **1i.**

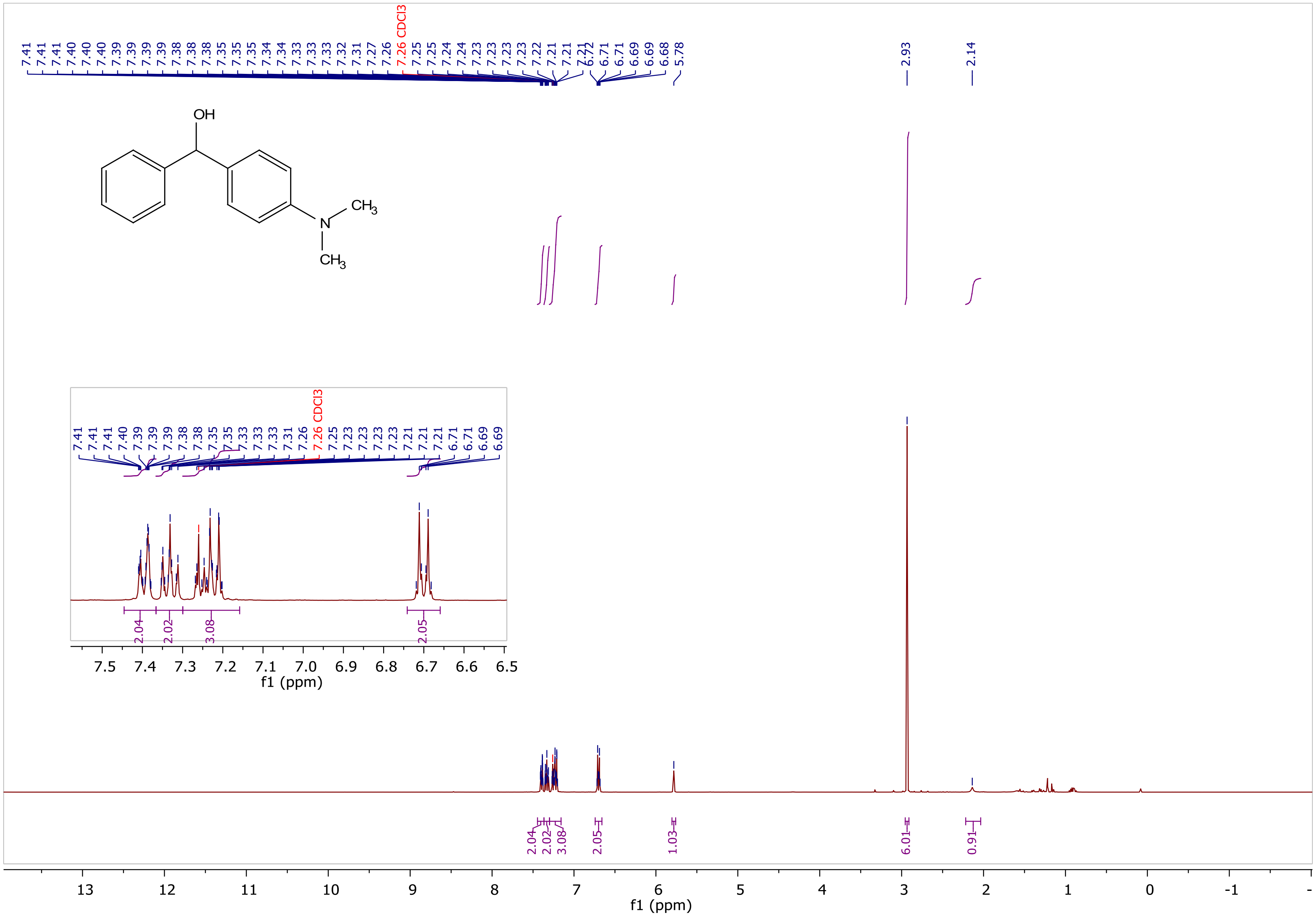

Figure S84. 1H-NMR (400 MHz CDCl3) of compound **1e**.

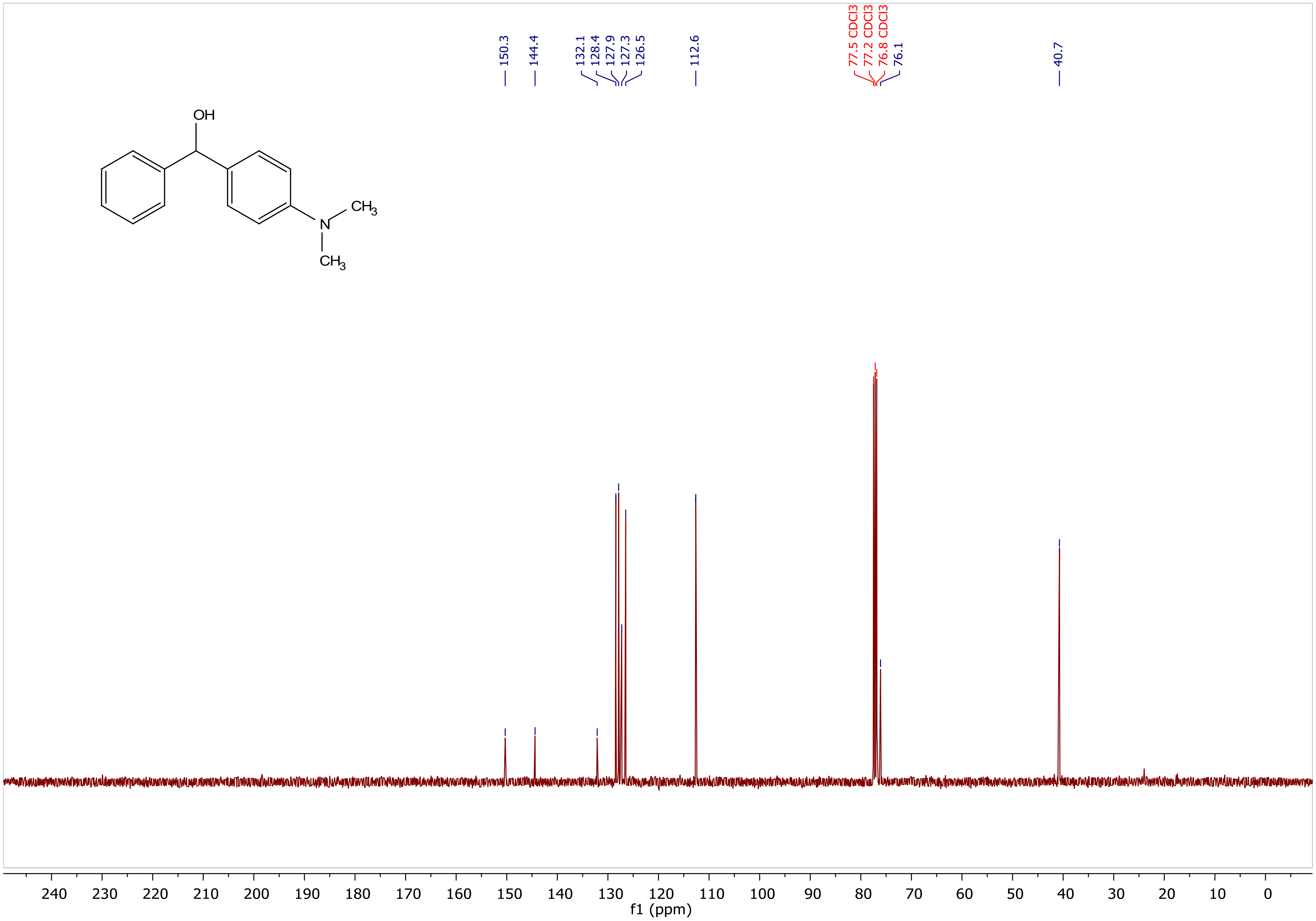

Figure S85. 13C{1H}-NMR (101 MHz, CDCl3) of compound **1e**.

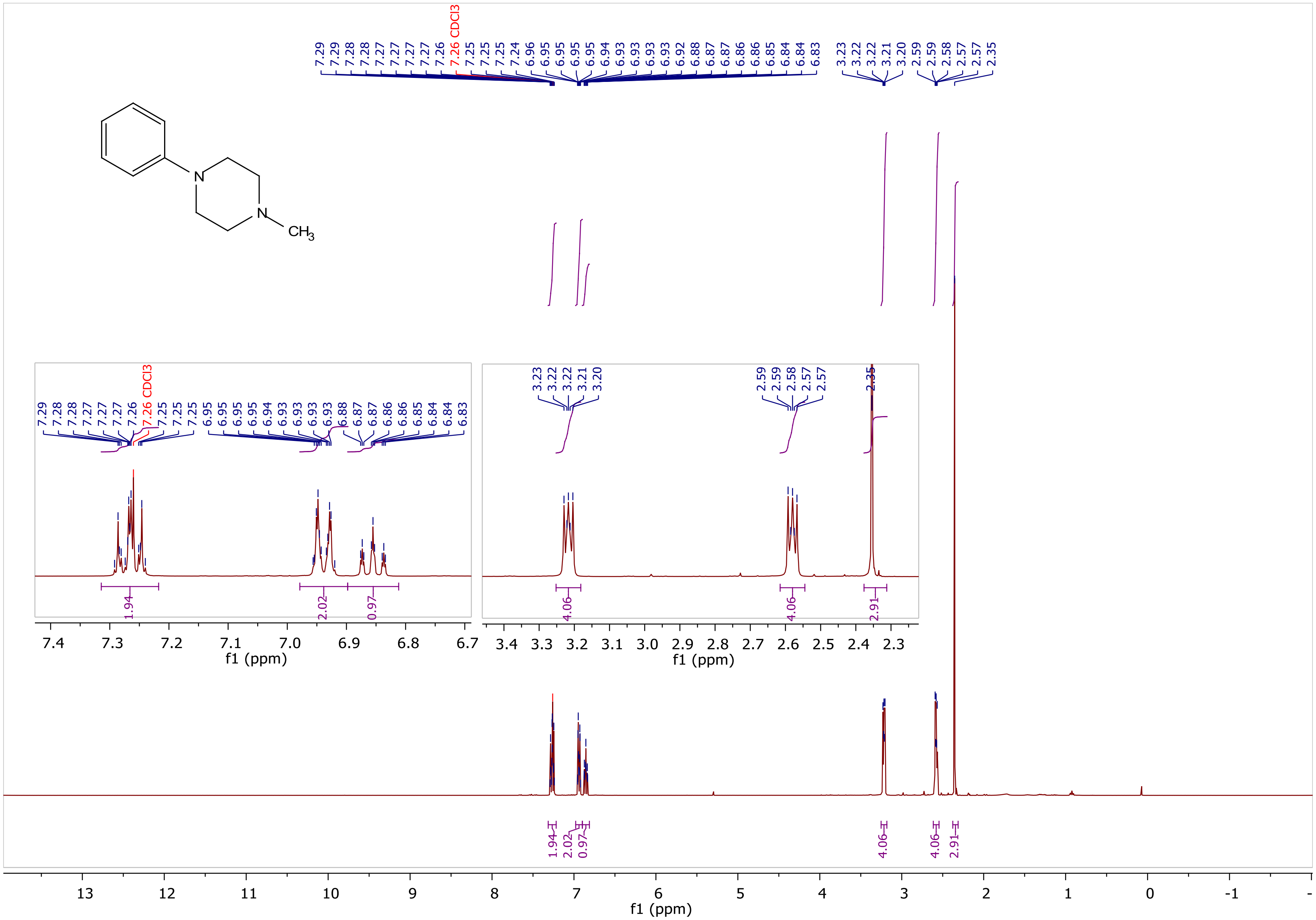

Figure S86. 1H-NMR (400 MHz CDCl3) of compound **3c**.

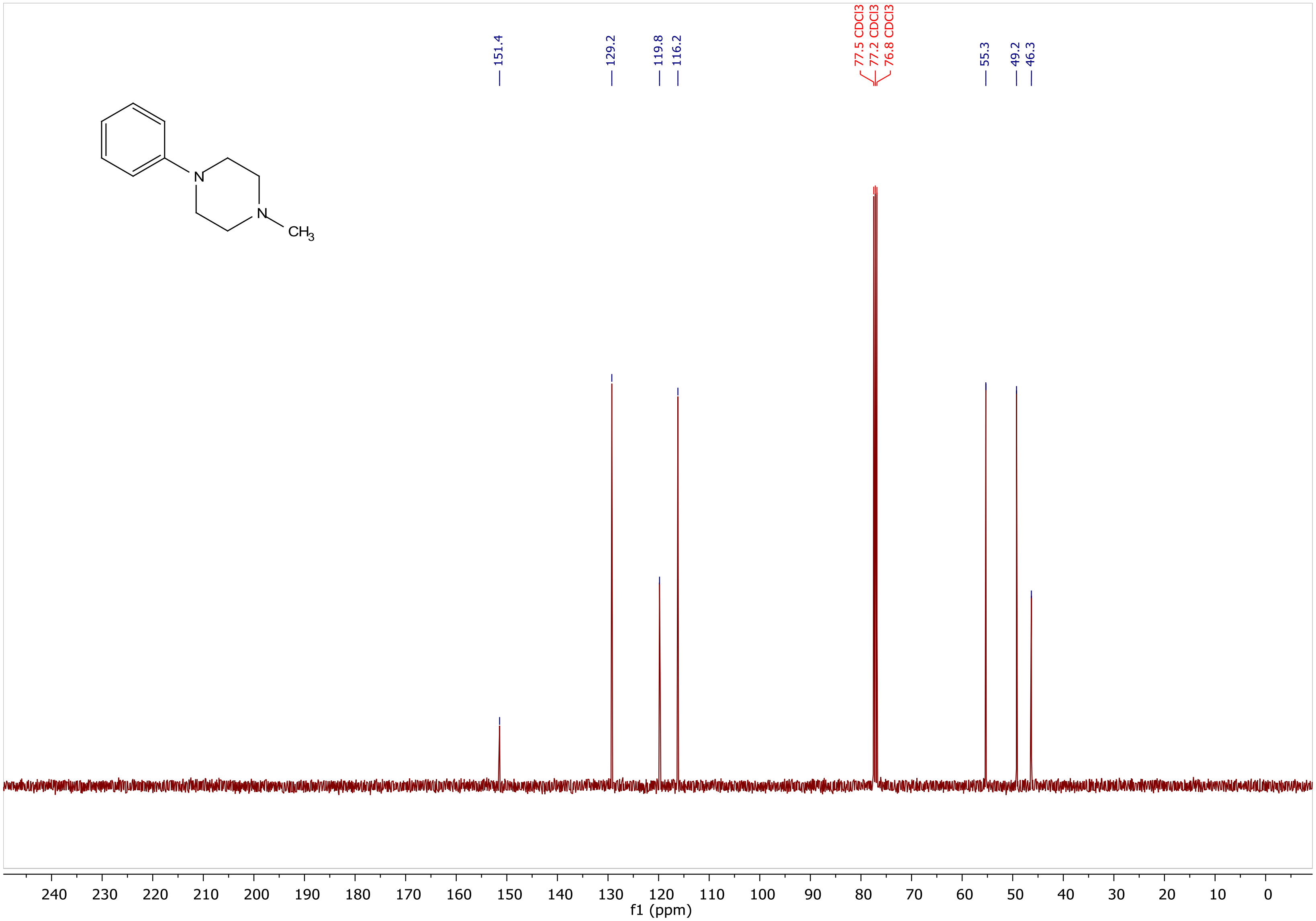

Figure S87. 13C{1H}-NMR (101 MHz, CDCl3) of compound **3c**.
